# Towards photoswitchable quadruple hydrogen bonds *via* a reversible “photolocking” strategy for photocontrolled self-assembly†

**DOI:** 10.1039/d0sc06141g

**Published:** 2020-12-15

**Authors:** Lu Wei, Shi-Tao Han, Ting-Ting Jin, Tian-Guang Zhan, Li-Juan Liu, Jiecheng Cui, Kang-Da Zhang

**Affiliations:** Key Laboratory of the Ministry of Education for Advanced Catalysis Materials, College of Chemistry and Life Science, Zhejiang Normal University 688 Yingbin Road Jinhua 321004 China tgzhan@zjnu.cn Kangda.Zhang@zjnu.cn

## Abstract

Developing new photoswitchable noncovalent interaction motifs with controllable bonding affinity is crucial for the construction of photoresponsive supramolecular systems and materials. Here we describe a unique “photolocking” strategy for realizing photoswitchable control of quadruple hydrogen-bonding interactions on the basis of modifying the ureidopyrimidinone (UPy) module with an *ortho*-ester substituted azobenzene unit as the “photo-lock”. Upon light irradiation, the obtained Azo-UPy motif is capable of unlocking/locking the partial H-bonding sites of the UPy unit, leading to photoswitching between homo- and heteroquadruple hydrogen-bonded dimers, which has been further applied for the fabrication of novel tunable hydrogen bonded supramolecular systems. This “photolocking” strategy appears to be broadly applicable in the rational design and construction of other H-bonding motifs with sufficiently photoswitchable noncovalent interactions.

## Abstract 2

A photolocking strategy is described to achieve the construction of effectively photoswitchable quadruple hydrogen bonds featuring with photoregulable H-bonding affinities, which is further applied in the photocontrollable H-bonded self-assemblies.

## Graphical abstract

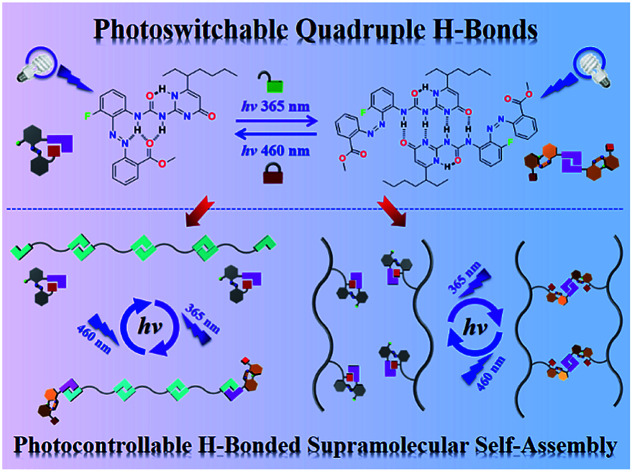

## Introduction

Hydrogen bonding is ubiquitously adopted by nature to form complex structures and implement delicate functions, as exemplified by DNA and proteins. It also plays a pivotal role in the construction of advanced supramolecular architectures^1–11^ as well as tuning their properties and functions.^12–18^ Among various H-bonding motifs, complementary multiple hydrogen bonding systems are particularly attractive due to their high affinity and selectivity, diverse binding modes and predictability for the resulting self-assembled structures.^19–21^ Over the past few decades, great efforts have been devoted to the development of complementary multiple H-bonding motifs.^22–37^ Meanwhile, by taking advantage of the stimuli-responsive nature of H-bonding interactions, considerable attention has been focused on the development of tunable H-bonded self-assemblies and materials with responsiveness to external stimuli such as temperature,^38,39^ pH,^32,40–42^ redox,^43–46^ light,^47–56^ and competitive guests.^57,58^

Compared to other stimuli, light has been recognized to be ideal due to its cleanness and unique spatiotemporal resolution,^59–62^ benefiting from which various sophisticated photoresponsive H-bonded systems have been constructed. For instance, Yang and co-workers have demonstrated a stiff stilbene based monomer with quadruple H-bonding units, which could undergo distinct ring-chain (for *Z*-isomer) or isodesmic growth (for *E*-isomer) polymerization mechanisms to form supramolecular polymers upon irradiation with different UV lights.^50^ The Yagai group has also developed a series of multiple H-bonds directed supramolecular toroidal building blocks to fabricate unique supramolecular polymeric systems with photoregulable topologies.^18,56^ Nevertheless, including these examples, most of the reported photoresponsive multiple H-bonded systems have been designed on the basis of molecular geometric variations of the building blocks which are induced by the photoisomerization of incorporated photochromophores,^48–56^ rather than directly photoswitching the association constants of multiple H-bonding interactions. However, impeded by the diversity in structures and bonding modes of multiple H-bonding motifs, as well as the strong structural matching requirement of multiple H-bonding interactions, it is challengeable to develop a general strategy for the construction of multiple H-bonding motifs with sufficiently photoswitchable H-bonding affinities.

In the past two decades, efforts have been devoted to exploring the potential of photocontrolling the dimerization bonding behavior between multiple H-bonding motifs.^63^ Current strategies include (1) achieving the photo-triggered formation of multiple H-bonds by using a deprotectable “photocage”,^64,65^ (2) changing the H-bonding affinity by photoregulating the geometry of the bonding cavity,^66^ or bonding site arrangement,^67^ (3) tuning the H-bonding ability of the H-bonded motifs through light/heating conversion,^68^ and (4) switching the H-bonding interactions *via* the reversible photoregulation of the stacking interaction,^69^ or the H-bond donating-accepting ability of the multiple H-bonding motifs.^70^ However, these systems are, to a considerable extent, constrained by irreversibility,^64,65^ non-bidirectional photoswitchability,^66,68^ or unsatisfactory photoswitching H-bonding affinities.^67–70^ In addition, when employing these strategies to construct photoswitchable multiple H-bonding motifs, their structures are often limited by strict requirements. Thus, these restrictions greatly discourage the current strategies from more innovative applications.

**Scheme 1:**
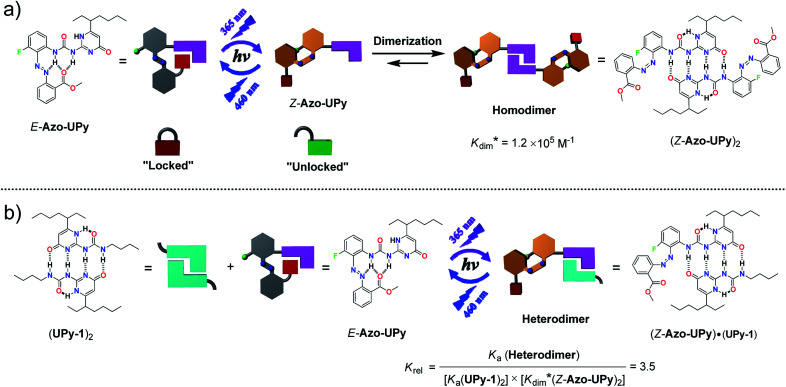
The chemical structures of the azobenzene-modified UPy derivative (Azo-UPy), and the schematic illustration of the “photolocking” strategy in the construction of the photoswitchable quadruple H-bonded (a) homodimer and (b) heterodimer. The 
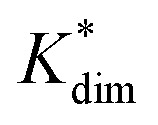
 value was used as an expression for the apparent dimerization constant of *Z*-Azo-Upy, due to the coexistance of tautomers I and II of (*Z*-Azo-UPy)_2_ in CDCl_3_ solution.

To break this logjam, we herein describe a reversible “photolocking” strategy, which allows for the achievement of bidirectional photoswitching of multiple H-bonding interactions by dramatically changing the binding affinity upon photoirradiation (Scheme 1). To implement this strategy, the widely used quadruple H-bonding motif, based on the ureidopyrimidinone (UPy) unit, was used and modified with a photoswitchable *ortho*-ester-substituted azobenzene moiety at the urea side, through which a unique photoresponsive UPy derivative of Azo-UPy was obtained (Scheme 1a). Before light irradiation, the two urea N–H hydrogens in the pristine *E*-Azo-UPy preferred to form intramolecular multiple H-bonds with the azo and carbonyl (C=O) groups, by which the formation of the quadruple H-bonded UPy dimer was immensely suppressed (“locked” state in Scheme 1a). Upon UV light irradiation, the *E* → *Z* photoisomerization of the azobenzene unit was triggered, which led to the breakage of the intramolecular H-bonds (“unlocked” state in Scheme 1a). The resulting *Z*-Azo-UPy molecule tended to form a quadruple H-bonded homodimer of (*Z*-Azo-UPy)_2_ driven by a strengthened binding between the UPy units. Upon blue light irradiation, the reverse *Z* → *E* photoisomerization of the azo units of the (*Z*-Azo-UPy)_2_ dimer was triggered again to dramatically weaken the dimerization, thus leading to the degradation of the (*Z*-Azo-UPy)_2_ dimer and the regeneration of the intramolecular H-bonding “locked” *E*-Azo-UPy. To the best of our knowledge, this is the first example of a quadruple H-bonding motif with bidirectionally and sufficiently photoswitchable H-bonding affinity.

We further demonstrate that the quadruple H-bonding of the non-photoactive UPy homodimer of (UPy-1)_2_ was not interfered when the “locked” *E*-Azo-UPy was introduced (Scheme 1b). However, after the *E*-Azo-UPy was irradiated with UV light in the presence of the (UPy-1)_2_ dimer, the quadruple H-bonded heterodimer of (*Z*-Azo-UPy)·(UPy-1) could be formed (Scheme 1b). Subsequent irradiation of this heterodimer with blue light could regenerate the “locked” *E*-Azo-UPy to cause the degradation of the heterodimer and the reformation of the homodimer of (UPy-1)_2_. Therefore, the homo-/heterodimerization behavior of such Azo-UPy molecules could be facilely photocontrolled through the reversible photoswitching of their dimerization association constant upon irradiation with lights of different wavelengths.

To further test the potential of such unique photoswitchale Azo-UPy motifs in the construction of photocontrollable self-assembly systems, we have also prepared an Azo-UPy-appended polymer and investigated its photocontrollable aggregation behavior by photoregulating the “on/off” of the quadruple H-bonding interactions between the Azo-UPy units on the side chains. In addition, we revealed that the Azo-UPy motif could work as a photoswitchable chain capper to achieve photocontrollable supramolecular polymerization.

## Results and discussion

### Photoswitching behavior of the model compounds

Model compounds 1a–1d (Table 1) were first designed and prepared to test the practicability of the “photolocking” strategy. Their UV-vis absorption spectra were recorded under irradiation with light sources of different wavelengths (Fig. S1†), according to which the optimal irradiation wavelengths could be determined when the contents of their *E*- or *Z*-isomers in the solution reached the maximum at the photostationary stationary state (PSS) (Table 1). To further investigate reversible photoisomerization, their ^1^H NMR spectra at PSS_*Z*_ or PSS_*E*_ were also recorded (Fig. S2–S9†), based on which their *E*/*Z* isomeric ratios were obtained and 1b was revealed to exhibit the best photoswitching behavior (Table 1).

**Table 1:** Photoswitching behavior of the model compounds 1a–1d

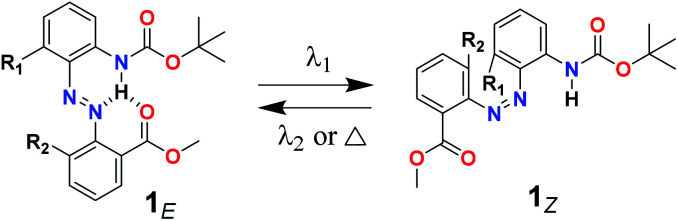			
Compounds	R_1_/R_2_	PSS (*E*/*Z*)	Light sources
1a	H/H	73/27	*λ*_2_ > 525 nm
		44/56	*λ*_1_ = 400 nm
1b	**F/H**	**73/27**	***λ***_**2**_ **= 460 nm**
		**26/74**	***λ***_**1**_ **= 350 nm**
1c	H/F	65/35	*λ*_2_ > 525 nm
		44/56	*λ*_1_ = 400 nm
1d	F/F	64/36	*λ*_2_ = 460 nm
		30/70	*λ*_1_ = 350 nm

The photocontrolled “on/off” of the intramolecular H-bonding of 1b was then disclosed. Before irradiation, the amide N–H hydrogen of 1b_*E*_ exhibited a downfield signal at 11.2 ppm (H_*E*_ in Fig. S10a†), indicative of the formation of strong intramolecular H-bonds. Upon irradiation with UV light (*λ* = 350 nm), the *E* → *Z* photoisomerization was triggered, during which the intramolecular H-bonding was ruptured due to the fact that the geometry of 1b_*Z*_ was unfavorable for the formation of such three-centered intramolecular H-bonding. In this case, the signal of the amide hydrogen of 1b_*Z*_ was found to upshift to 7.3 ppm (H_*Z*_ in Fig. S10b†). The intramolecular H-bonding could be formed again after the UV-irradiated 1b was exposed to the blue light (*λ* = 460 nm), which was supported by the restoration of the downfield signal of the amide hydrogen (Fig. S10c†). These results clearly demonstrated that the design strategy of introducing the *ortho*-ester-substituted azobenzene for photoswitching could achieve photocontrolled breakage/recovery of the intramolecular H-bonding in compound 1 (Table 1).

### Photoswitchable quadruple H-bonding self-dimerization behavior of **Azo-Upy**

**Scheme 2:**
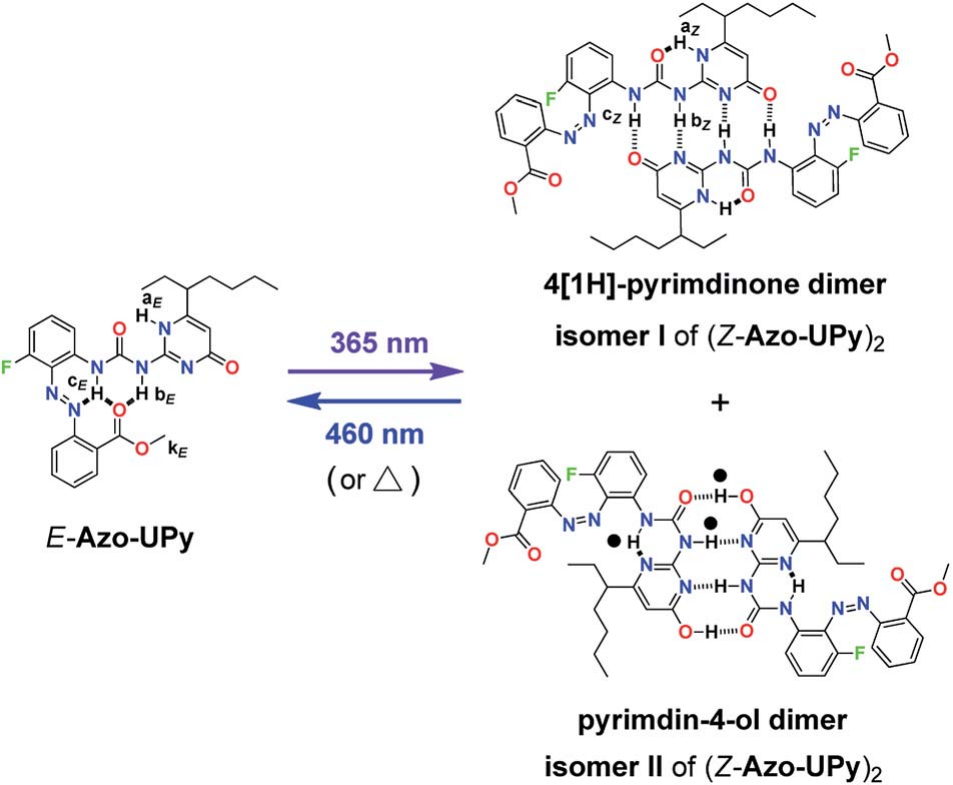
Chemical structure of Azo-UPy and the schematic representation of its photocontrolled quadruple H-bonding self-dimerization behavior.

**Fig. 1:**
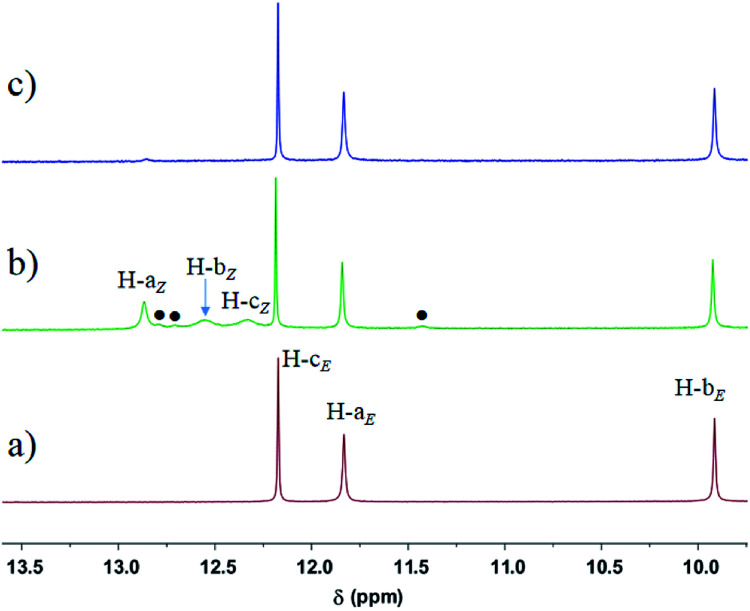
Partial ^1^H NMR spectra (600 MHz, 10 mM, CDCl_3_, 298 K) of the solution of (a) *E*-Azo-UPy, (b) the PSS_*Z*_ (365 nm) mixtures of Azo-UPy, and (c) the PSS_*E*_ (460 nm) mixtures of Azo-UPy. The signals (●) are from N–H protons of the pyrimidin-4-ol dimer (*Z*-Azo-UPy)_2_ (isomer II in Scheme 2).

Based on the above investigation of the model compounds, we then prepared a photoresponsive UPy derivative Azo-UPy (Scheme 2). Prior to light irradiation, the formation of the intramolecular H-bonding between the urea N–H hydrogens and the azo and ester carbonyl groups, in other words, the “locking” state of Azo-UPy, was carefully evaluated through a series of NMR spectroscopic investigations. Firstly, the ^1^H NMR spectrum of pure *E*-Azo-UPy in CDCl_3_ was recorded (Fig. 1a and S19a†), from which the proton signals (H-a_*E*_ and H-b_*E*_) of the UPy unit were found to appear at 11.8 (H-a_*E*_) and 9.9 ppm (H-b_*E*_), respectively. Both signals upshifted as compared to those of the quadruple H-bonded UPy units, which typically appeared at around 13 and 12 ppm, respectively.^71–73^ In particular, the chemical shift of H-a_*E*_ (11.8 ppm) of *E*-Azo-UPy was close to that of the monomeric UPy unit (*δ*(H-a_*E*_) ≈ 11.9 ppm) in CDCl_3_.^74^ These results implied that *E*-Azo-UPy was unable to efficiently form quadruple H-bonded dimers.

Notably, the N–H hydrogen H-c_*E*_ of *E*-Azo-UPy exhibited the maximal downfield shift (*δ* = 12.1 ppm), which indicated that H-c_*E*_ might form three-centered N⋯H⋯O intramolecular H-bonding with the azo and carbonyl groups (Scheme 2). The chemical shift of H-b_*E*_ (9.9 ppm) in the UPy unit of *E*-Azo-UPy shifted significantly downfield as compared to that of free N–H(b) (*δ* < 8.8 ppm) in the monomeric UPy unit in CDCl_3_.^73^ Meanwhile, strong NOEs were observed between H-b_*E*_ and H-k_*E*_ of the methoxyl group (Fig. S12†), which also revealed the formation of intramolecular H-bonding between H-b_*E*_ and the carbonyl group. All these results supported that the two urea N–H hydrogens (H-b_*E*_ and H-c_*E*_) of *E*-Azo-UPy formed intramolecular H-bonding with ester carbonyl groups (Scheme 2). In this way, the quadruple H-bonding sites of the UPy unit of *E*-Azo-UPy were partially locked, which greatly disabled the formation of intermolecular quadruple H-bonded dimers.

To further disclose the efficiency of dimerization inhibition, we recorded the ^1^H NMR spectra of *E*-Azo-UPy of varying concentrations in CDCl_3_ (Fig. S14†), from which only one set of well-defined proton signals was observed in the concentration range of 0.25–40 mM. This might be either attributed to the fact that *E*-Azo-UPy was unable to form the quadruple H-bonded dimer, or the interconversion rate between the dimeric and monomeric *E*-Azo-UPy was faster than the NMR time scale. However, it was noteworthy that the signals of all the N–H hydrogens of *E*-Azo-UPy slightly shifted upfield (−0.09 ppm < Δ*δ* < 0), probably owing to the weak π–π stacking of *E*-Azo-UPy, rather than shifted downfield as the concentration increased. In addition, when competitive DMSO-*d*_6_ (v%, 0–10%) was introduced into the solution, the spectra did not exhibit two sets of signals, which were expected to be generated by the coexisting quadruple H-bonded dimer and the uncomplexed monomer (Fig. S15†), respectively. These observations supported that the quadruple H-bonding dimerization of *E*-Azo-UPy was greatly restricted. This conclusion was further evidenced by the observation that the signals for the UPy unit of *E*-Azo-UPy remained unchanged after introducing a non-photoactive quadruple H-bonded homodimer of (UPy-1)_2_ into the solution of *E*-Azo-UPy (Fig. S16†). In this case, if the *E*-Azo-UPy was capable of forming quadruple H-bonding, the signals should be changed due to the formation of the heterodimer between *E*-Azo-UPy and UPy-1, which was actually not observed.

After the evaluation of the intramolecular H-bonding of *E*-Azo-UPy, we then investigated its phototriggered quadruple H-bonding dimerization. Firstly, the UV-vis absorption spectra of Azo-UPy at PSS of different light sources were recorded (Fig. S17†), from which the optimal irradiation lights were determined as *λ* = 365 nm (for *E* → *Z*) and *λ* = 460 nm (for *Z* → *E*), respectively, since the highest *Z*/*E* (or *E*/*Z*) isomeric ratios of Azo-UPy at PSS were achieved upon irradiation with these lights. Azo-UPy was found to exhibit excellent light fatigue resistance as revealed through repeated irradiation experiments (Fig. S18†). The ^19^F NMR spectra of Azo-UPy at PSS_*Z*_ (365 nm) were then recorded (Fig. S20b and S21a†), based on which the isomeric ratio was calculated as *E*/*Z* = 47/53, and the content ratio of the two isomers of *Z*-Azo-UPy in the solution could be further determined as isomer I/isomer II = 10/1, whereas the thermal stability of the obtained *Z*-Azo-UPy was measured as *t*_1/2_(*Z* → *E*) = 64.8 h (Fig. S22 and S23†).

The phototriggered unlocking of the intramolecular H-bonding and the quadruple H-bonding of Azo-UPy at PSS_*Z*_ (365 nm) were studied using ^1^H NMR (Fig. S1b and S19b†), which exhibited two new sets of signals. One signal (H-a_*Z*_, H-b_*Z*_ and H-c_*Z*_) corresponded to the UPy unit of the 4[1*H*]-pyrimidinone dimer (isomer I in Scheme 2), and the other (black dotted signals in Fig. 1b) could be assigned to the UPy unit of the pyrimidin-4-ol tautomer of (*Z*-Azo-UPy)_2_ (isomer II in Scheme 2). As revealed in Fig. 1b, the chemical shifts of the N–H signals (H-a_*Z*_, H-b_*Z*_ and H-c_*Z*_) for isomer I of (*Z*-Azo-UPy)_2_ appeared at 12.9, 12.5 and 12.3 ppm, respectively. For isomer II, the signals of the protons were at 12.8, 12.7 and 11.4 ppm, respectively. Compared to those of *E*-Azo-UPy (Fig. 1a), the N–H signals of H-a_*Z*_ and H-b_*Z*_ for isomer I of the (*Z*-Azo-UPy)_2_ dimer underwent downfield shifting. Moreover, the chemical shifts of the N–H signals of *Z*-Azo-UPy were close to those of the reported quadruple H-bonded dimers constructed by 2-Aryl UPy derivatives.^71–73^ Based on these observations (Table 2), we proposed that quadruple H-bonding was formed between the UPy units of two *Z*-Azo-UPy molecules at PSS_*Z*_ (365 nm).

**Table 2:** Data for Azo-UPy determined by NMR experiments in CDCl_3_ at 298 K

Compound	Chemical shift/ppm			*D* c/10^−10^ (m^2^ s^−1^)	*K*_dim_ (M^−1^)
	H-a	H-b	H-c		
*E*-Azo-UPy	11.8^a^	9.9^a^	12.1^a^	6.31	nd^d^
*Z*-Azo-UPy	12.9^b^	12.5^b^	12.3^b^	4.57	1.2 × 10^5^^e^

a The concentration was 10 mM.

b The chemical shift of N–H signals for the 4[1*H*]-pyrimidinone dimer (isomer I).

c The diffusion coefficients.

d The value could not be determined as H-bonding dimerization between *E*-Azo-UPy was effectively restricted.

e This value refers to the apparent dimerization constant 
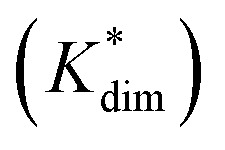
 of *Z*-Azo-UPy.

To get deep insight into the phototriggered quadruple H-bonding dimerization of Azo-UPy, 2D DOSY NMR experiments were further carried out. Before irradiation, *E*-Azo-UPy was found to diffuse as one entity with a single diffusion coefficient of *D* = 6.31 × 10^−10^ m^2^ s^−1^ (Fig. S27†). After irradiation with UV light (365 nm), two individually diffused entities were observed from the resulting PSS_Z_ mixtures of Azo-UPy with the diffusion coefficients of *D*_1_ = 4.57 × 10^−10^ m^2^ s^−1^ and *D*_2_ = 6.31 × 10^−10^ m^2^ s^−1^ (Fig. S28†), which could be assigned to the formed quadruple H-bonded (*Z*-Azo-UPy)_2_ dimer and the *E*-Azo-UPy monomer, respectively. Based on the ratio of *D*_1_/*D*_2_ = 72.9%, the volume for the defusing entity formed by *Z*-Azo-UPy could be estimated as 2.6 times bigger than that of *E*-Azo-UPy, which further supported the formation of the quadruple H-bonded (*Z*-Azo-UPy)_2_ dimer. In addition, the influence of solvent polarity on the dimerization behavior was also revealed by the recorded 2D DOSY-NMR spectra of Azo-UPy at PSS in the strong H-bonding competitive polar solvent of DMSO-*d*_6_. While the pristine *E*-Azo-UPy in DMSO-*d*_6_ solution revealed a single diffusion coefficient of *D* = 1.41 × 10^−10^ m^2^ s^−1^ (Fig. S29†), the two components of *E*-Azo-UPy and *Z*-Azo-UPy in the PSS_*Z*_ (365 nm) mixtures of the DMSO-*d*_6_ solution of Azo-UPy were found to diffuse as species of close volume with an equal diffusion coefficient of *D* = 1.48 × 10^−10^ m^2^ s^−1^ (Fig. S30†). This observation suggested that the quadruple H-bonded (*Z*-Azo-UPy)_2_ dimer could only be formed in weakly polar solvents such as chloroform, and the quadruple H-bonds between the *Z*-Azo-UPy molecules were hardly formed in the solution of DMSO-*d*_6_, in which both the *E*- and *Z*-isomers of Azo-UPy existed as monomers.

The dimerization stability of *Z*-Azo-UPy was further investigated through ^1^H and ^19^F NMR dilution experiments of the PSS_*Z*_ (365 nm) mixtures of Azo-UPy in CDCl_3_. As the concentration decreased from 20 mM to 0.25 mM, the proton (Fig. S31†) and fluorine (Fig. S33†) signals of the monomeric *Z*-Azo-UPy were observed, and the dimerization constant in the formation of the (*Z*-Azo-UPy)_2_ dimer was estimated to be 
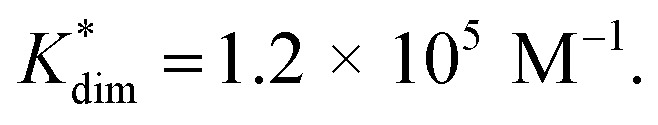
 In addition, the ability of *Z*-Azo-UPy to form the quadruple H-bonded (*Z*-Azo-UPy)_2_ dimer was sensitive to the polarity of the solvent, as proved by the concurrent observation of the proton (Fig. S34†) or fluorine (Fig. S35†) signals referring to the non H-bonded monomeric *Z*-Azo-UPy and the quadruple H-bonded dimer, respectively, when a small amount of DMSO-*d*_6_ was introduced into the CDCl_3_ solution of the PSS_*Z*_ (365 nm) mixtures of Azo-UPy.

Furthermore, when the UV-irradiated PSS_*Z*_ (365 nm) solution of Azo-UPy was exposed to the blue light of *λ* = 460 nm, the reversed *Z* → *E* photoisomerization of the azo group could be triggered, during which the quadruple H-bonded (*Z*-Azo-UPy)_2_ dimer was degenerated and the intramolecular H-bonding locked *E*-Azo-UPy could be regenerated, as evidenced by the observation of dramatically decreased proton signals of the *Z*-isomer and the significantly increased proton signals of the *E*-isomer of Azo-UPy at PSS_*E*_ (460 nm) (Fig. 1c and S19c†). Besides, the isomeric ratio of *E*/*Z* (92/8) could be calculated based on the recorded ^19^F NMR spectrum of Azo-UPy at PSS_*E*_ (460 nm) (Fig. S20†). These results indicated that the quadruple H-bonding behavior of the Azo-UPy was bidirectionally photoswitchable.

### Photoswitchable quadruple H-bonding hetero-dimerization behavior of **Azo-Upy**

**Scheme 3:**
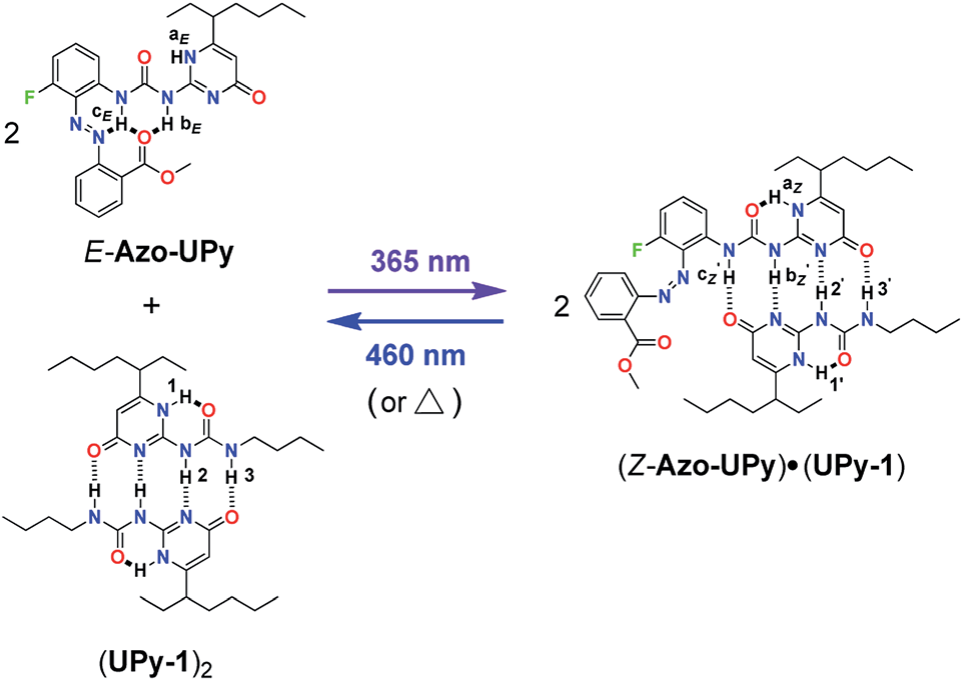
The schematic representation of the photocontrolled quadruple H-bonding hetero-dimerization behavior of Azo-UPy and UPy-1.

**Fig. 2:**
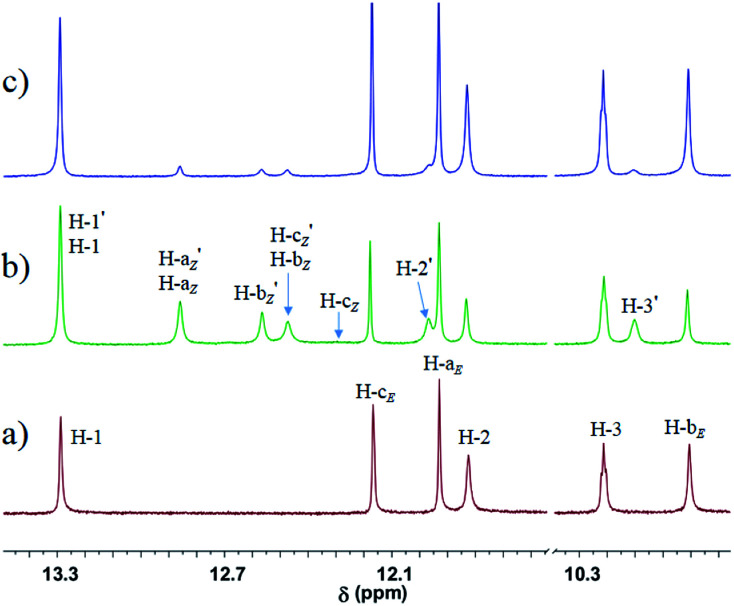
Partial ^1^H NMR spectra (600 MHz, CDCl_3_, 298 K) of (a) the solution of *E*-Azo-UPy and UPy-1 before irradiation, (b) the PSS_*Z*_ (365 nm) mixtures of Azo-UPy and UPy-1, and (c) the PSS_*E*_ (460 nm) mixtures of Azo-UPy and UPy-1. The concentrations of Azo-UPy and UPy-1 were 10 mM, respectively.

After the above investigation of the reversible photocontrolled quadruple H-bonding self-dimerization of Azo-UPy, we further applied the new motif to photoregulate the quadruple H-bonding dimerization of nonphotoactive UPy-1 (Scheme 3). To this end, *E*-Azo-UPy was firstly mixed with UPy-1 in CDCl_3_, where *E*-Azo-UPy existed as the monomer with an intramolecularly H-bonding locked UPy unit, while UPy-1 existed as the quadruple H-bonded dimer of (UPy-1)_2_ (Fig. 2a). Upon irradiation with UV light (365 nm), the *E* → *Z* photoisomerization of Azo-UPy in the solution was triggered to generate *Z*-Azo-UPy, which was found to be capable of decoupling the (UPy-1)_2_ dimer through the formation of the quadruple H-bonded heterodimer of (*Z*-Azo-UPy)·(UPy-1), as proved by the observation of newly appeared proton signals (H-1′ ∼ H-3′ and 
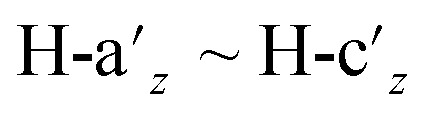
 in Fig. 2b). The proton (Fig. S40†) or fluorine (Fig. S44†) signals of the heterodimer of (*Z*-Azo-UPy)·(UPy-1) were found to be emerged and enhanced significantly at PSS_*Z*_ (365 nm) as the concentration of UPy-1 increased, while the corresponding signals for the homodimers of (UPy-1)_2_ and (*Z*-Azo-UPy)_2_ decreased accordingly. The relative association constant of the heterodimer could be obtained as *K*_rel_ = 3.5, based on the ^1^H NMR spectra (Fig. S45†). Notably, the formation of the heterodimer was found to be capable of improving the *E* → *Z* photoisomerization ratio of Azo-UPy, as suggested by the *E*/*Z* (42/58) value calculated from the ^19^F NMR spectrum of the PSS_*Z*_ (365 nm) mixtures of Azo-UPy and UPy-1 (Fig. S43†).

When the UV-irradiated solution of the PSS_*Z*_ (365 nm) mixtures of Azo-UPy and UPy-1 was further irradiated with blue light (*λ* = 460 nm), the *Z* → *E* photoisomerization of the azo group in the heterodimer of (*Z*-Azo-UPy)·(UPy-1) was induced, leading to the degradation of the heterodimer and the reconstruction of the homodimer, as evidenced by the observation of dramatically decreased proton signals for the (*Z*-Azo-UPy)·(UPy-1), as well as the enhanced proton signals for (UPy-1)_2_ and *E*-Azo-UPy in the PSS_*E*_ (460 nm) mixtures of Azo-UPy and UPy-1 (Fig. 2c). The isomeric ratio (*E*/*Z* = 90/10) of Azo-UPy was obtained based on ^19^F NMR of the PSS_*E*_ (460 nm) mixtures of Azo-UPy and UPy-1 (Fig. S43†). All these investigations supported that Azo-UPy not only exhibited unique photocontrollable quadruple H-bonding dimerization, but also was able to photoregulate the quadruple H-bonding dimerization behavior of the nonphotoactive UPy derivative.

### Application of **Azo-UPy** for photocontrollable macro-/molecular self-assembly

**Fig. 3:**
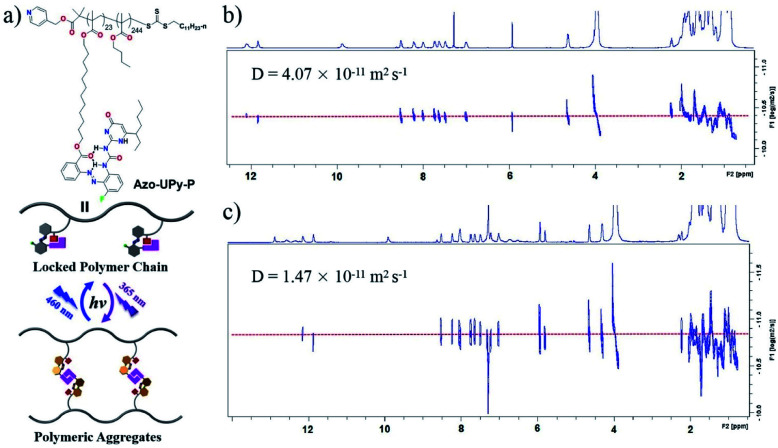
(a) Chemical structure of Azo-UPy modified polymer Azo-UPy-P, and the schematic representation of its photocontrolled macromolecular self-assembly behavior. The 2D DOSY spectra (600 MHz, 40 mg mL^−1^, CDCl_3_, 298 K) of polymer Azo-UPy-P under conditions of (b) before and (c) after irradiation with UV light (365 nm).

Benefiting from the dynamic nature, good directionality, versatility and high stability, multiple H-bonded motifs have been integrated into the polymeric scaffolds to fabricate H-bond cross-linked polymeric materials with tunable microstructures and high-performances.^75–82^ To further apply Azo-UPy for the construction of photocontrollable macromolecular systems, a linear polymer Azo-UPy-P was then synthesized, with the side chains being modified with Azo-UPy (Fig. 3a). The photocontrolled aggregation behavior of Azo-UPy-P was then investigated by 2D DOSY-NMR spectroscopy. Before UV light irradiation, the UPy units in the side chains were locked by intramolecular H-bonding, as indicated by the observation of N–H signals (Fig. S46 and S47a†) with chemical shifts similar to those of *E*-Azo-UPy (Fig. 1a), and the recorded 2D DOSY-NMR spectrum of the pristine state Azo-UPy-P was found to exhibit a single diffusion coefficient of *D* = 4.07 × 10^−11^ m^2^ s^−1^ (Fig. 3b).

Upon irradiation with UV light (365 nm), *E*-Azo-UPy in the side chains of Azo-UPy-P were converted to *Z*-Azo-UPy, leading to the unlocking of Azo-UPy and thus enabling the macromolecular aggregation of Azo-UPy-P through the formation of intermolecular quadruple H-bonds between the *Z*-Azo-UPy units of different polymeric chains (Fig. S47b†), as evidenced by the observation that the PSS_*Z*_ (365 nm) solution of Azo-UPy-P gave rise to a reduced diffusion coefficient of *D* = 1.47 × 10^−11^ m^2^ s^−1^ (Fig. 3c), which was about 36% of that of Azo-UPy-P at the pristine state. Accordingly, the volume of the UV-irradiated Azo-UPy-P was estimated as bigger as 21 times that of the non-irradiated Azo-UPy-P. Further exposing the solution of the UV-irradiated Azo-UPy-P to blue light (460 nm) caused a drastic decrease of the signals of the dimerized *Z*-Azo-UPy motifs (Fig. S47c†), suggesting the disassembly of the polymeric aggregation. However, after the low concentrated solution of Azo-UPy-P was irradiated with UV light, a decrease of the hydrodynamic volume of the polymer chain of Azo-UPy-P was observed (Fig. S48†), which could be attributed to the intramolecular chain collapse of the single polymer chain of Azo-UPy-P driven by the intra chain H-bonding interactions between the *Z*-Azo-UPy units.^83,84^

**Fig. 4:**
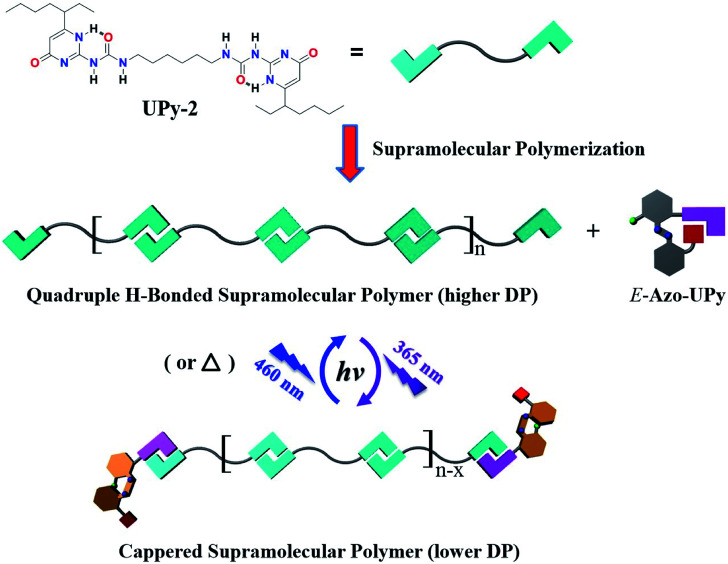
Chemical structure of UPy-2, and the schematic representation for the photocontrolled supramolecular polymerization of UPy-2 in the presence of Azo-UPy.

**Fig. 5:**
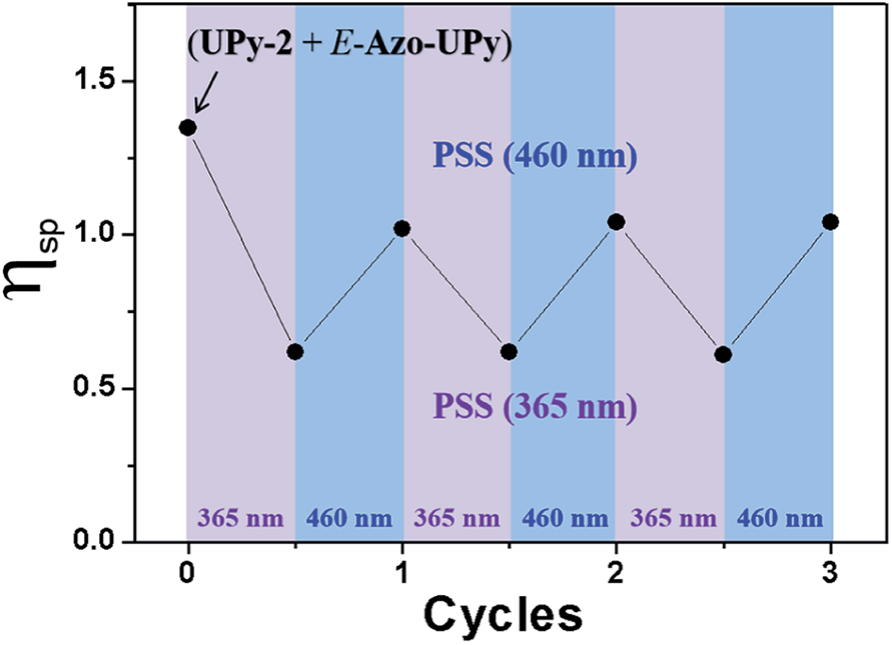
Recorded specific viscosity changes for the chloroform solution of *E*-Azo-UPy (1.0 mM) and UPy-2 (40 mM), upon irradiation with the light sources of 365 nm and 460 nm alternately.

In addition to the above polymeric system, the application of the Azo-UPy motif in photoregulating the self-assembly of small molecules was also explored. To this end, Azo-UPy was employed as a photoswitchable chain capper to realize photoregulable H-bonding supramolecular polymerization. A nonphotoactive AA type monomer bifunctionalized with two terminal UPy units was thus prepared (UPy-2 in Fig. 4). The molecule was dissolved in CDCl_3_ to generate a linear quadruple H-bonded supramolecular polymer (Fig. 4).^22,57^ When 0.025 equivalent of the non-irradiated *E*-Azo-UPy was introduced into the solution, neither chemical shifting changes for these two components nor new proton signals were observed (Fig. S49a–c†), indicating that the introduced *E*-Azo-UPy with a locked UPy unit did not obviously disturb the H-bonded supramolecular polymer based on UPy-2. However, after UV (365 nm) irradiation, 62% of *E*-Azo-UPy in the mixture was converted to *Z*-Azo-UPy with an unlocked UPy unit. As a result, quadruple H-bonding hetero-dimerization occurred between *Z*-Azo-UPy and UPy-2 (Fig. 4), with the result that the degree of polymerization (DP) of the H-bonded supramolecular polymer decreased (Fig. S49d and S50b†). In contrast, when this UV-irradiated solution of the supramolecular polymer with lower DP was further irradiated with blue light (460 nm) to reach PSS_*E*_, 82% of the capped *Z*-Azo-UPy was converted back to *E*-Azo-UPy with a locked UPy unit. Thus, the DP of the supramolecular polymer increased again (Fig. S49e and S50c†). To further visualize the photoregulable supramolecular polymerization of such a quadruple H-bonded system, the viscosity variations of the chloroform solution of the supramolecular polymer during the repeated photoswitching cycles were measured. As displayed in Fig. 5, after introducing *E*-Azo-UPy into the solution of UPy-2, the decrease/increase of the specific viscosity (*η*_sp_) of the solution could be photoswitched through irradiation with the light sources of 365 nm/460 nm alternately.

## Conclusions

In summary, we have demonstrated the rational design and construction of a unique photoswitchable UPy motif (Azo-UPy) with an *ortho*-ester-modified azobenzene unit through the reversible *E*/*Z* photoisomerization of which the two urea N–H hydrogens of Azo-UPy could be unlocked/locked *via* forming intramolecular H-bonds with the carbonyl groups, thereby enabling the photocontrolled quadruple H-bonding self-/hetero-dimerization of Azo-UPy molecules. Notably, the dimerization affinity of Azo-UPy can be dramatically changed upon alternating irradiation with UV and blue lights, and this feature has distinguished Azo-UPy-based self-assembled systems from most of the reported photoresponsive systems. Benefiting from such distinctive photoswitchable quadruple H-bonding, novel photocontrolled supramolecular systems can be facilely constructed by structurally integrating the Azo-UPy motif into polymer chains or employing it as a photoresponsive chain capper to regulate the quadruple H-bonding supramolecular polymerization. In light of the wide application of multiple H-bonding interactions in supramolecular chemistry and materials science, the results presented in this work provided a fundamental design strategy, ultimately giving access to the generation of photoresponsive supramolecular self-assembled systems such as self-assembled supramolecular hosts, as well as advanced materials featuring appealing properties of photocontrolled self-healing, phase transformation, dynamic adhesion and so on. In addition, the implementation of such a “photo-locking” strategy has no special requirement for the structural skeleton of multiple H-bonding motifs, which makes it broadly applicable in the construction of other types of photoswitchable multiple H-bonding motifs.

## Conflicts of interest

There are no conflicts to declare.

## Supplementary Material

SC-012-D0SC06141G-s001
