# Supplementary material for: Towards photoswitchable quadruple hydrogen bonds via a reversible “photolocking” strategy for photocontrolled self-assembly
Source: Chem Sci. 2020 Dec 15;12(5):1762–71. doi: 10.1039/d0sc06141g (PMC8179285; doi:10.1039/d0sc06141g)
Supplement: SC-012-D0SC06141G-s001 [file SC-012-D0SC06141G-s001.pdf]

Electronic Supplementary Material (ESI) for Chemical Science.
This journal is © The Royal Society of Chemistry 2020

Electronic Supplementary Information

# Towards photoswitchable quadruple hydrogen bonds *via* reversible "photolocking" strategy for photocontrolled self-assembly

Lu Wei,‡ Shi-Tao Han,‡ Ting-Ting Jin, Tian-Guang Zhan,* Li-Juan Liu, Jiecheng Cui and Kang-Da Zhang*

Key Laboratory of the Ministry of Education for Advanced Catalysis Materials, College of Chemistry and Life Sciences, Zhejiang Normal University, 688 Yingbin Road, Jinhua 321004, China.
E-mails: tgzhan@zjnu.cn; Kangda.Zhang@zjnu.cn
‡ These authors contributed equally to this work

## Table of Contents

Section 1: General methods and materials ........ S2
Section 2: Synthesis procedures and characterizations ........ S3
Section 3: Photoswitching behavior of model compounds ........ S12
Section 4: Photoswitchable quadruple H-bonded self-dimerization of **Azo-UPy** ........ S17
Section 5: Photoswitchable quadruple H-bonded hetero-dimerization of **Azo-UPy** ........ S33
Section 6: Application of **Azo-UPy** for photocontrollable macro-/molecular self-assembly ........ S41
Section 7: $^{1}H$ and $^{13}C$ NMR spectra for new compounds ........ S47
Section 8: GPC trace of the polymer **Azo-UPy-P**........S65
References ........ S65

## Section 1: Materials and general methods

**Materials.** All reagents were used as received from the commercial suppliers without further purification; the solvents have been purified by standard procedures before use. Compounds **2**[1], **3**[2], **4**[3], **5**[4], **8**[5] and **17**[6] were synthesized according to the reported procedures.

**Nuclear magnetic resonance (NMR).** The solution $^{1}H$ NMR and $^{13}C$ NMR spectra were recorded on a Bruker AVANCE 600 spectrometer with the chemical shifts (δ in ppm) were determined with a residual proton of the solvent as standard, the 2D COSY, NOESY and DOSY NMR spectra were collected on a Bruker AVANCE 600 spectrometer.

**UV-Vis spectroscopy.** The UV-Vis absorption spectra were recorded on an Agilent Technologies Cary 60 UV-Vis spectrometer. The irradiation experiments were performed by using a 50 mW/cm$^2$ LED lamp (365 nm) and a 20 mW/cm$^2$ LED lamp (460 nm), respectively.

**Viscosity measurement.** The viscosity of the sample was measured with a typical Ubbelohde micro-viscometer (with the inner diameter of 1.0 mm) at 25 °C in chloroform. The sample was alternatively irradiated by using a 50 mW/cm$^2$ LED lamp (365 nm) and a 20 mW/cm$^2$ LED lamp (460 nm), respectively.

## Section 2: Synthesis procedures and characterization

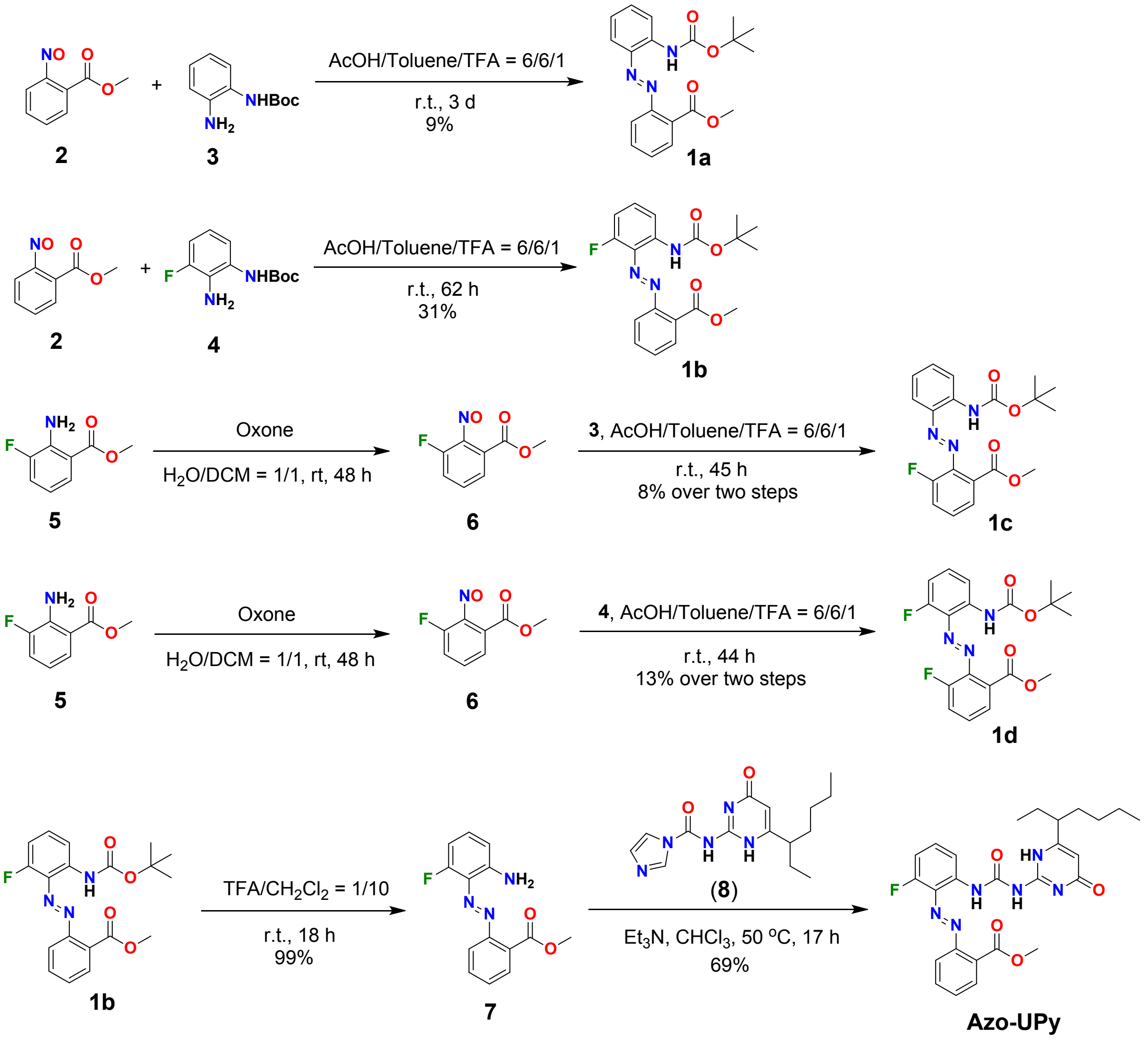


**Scheme S1.** The synthetic routs for the model compounds (**1a** - **1d**) and **Azo-UPy**.

**Compound 1a**: Compound **2**[1] (0.825 g, 5.00 mmol) and **3**[2] (1.04 g, 5.00 mmol) were dissolved in a ternary solvent of AcOH/toluene/TFA (v/v/v = 6/6/1) (21.6 mL), and the resulting mixture was then stirred at room temperature for 3 days. After TLC indicating the reaction was completed, the reaction mixture was adjusted to neutral by using saturated $NaHCO_3$ aqueous solution. Then, $CH_2Cl_2$ (100 mL) was added to the mixture for extraction, and the organic phase was collected and dried by anhydrous $Na_2SO_4$. The desiccant was removed by filtration, and the filtrate was concentrated by evaporation under reduced pressure. The resulting residue was further purified by flash column chromatography using the eluent of PE/EtOAc = 30/1, after which compound **1a** could be isolated as orange solid (0.160 g, 9%). $^1$H NMR (600 MHz, $CDCl_3$, 298 K) δ: 9.44 (s, 1H), 8.44 (d, $J$ = 8.4 Hz, 1H), 7.86-7.32 (m, 2H), 7.64-7.59 (m, 2H), 7.50 (td, $J_1$ = 6.6 Hz, $J_2$ = 1.8 Hz, 1H), 7.45 (t, $J$ = 7.8 Hz, 1H), 7.10 (t, $J$ = 7.8 Hz, 1H), 3.91 (s,

3H), 1.56 (s, 9H). $^{13}$C NMR (150 MHz, $CDCl_3$, 298 K) δ: 167.99, 152.87, 151.68, 138.97, 136.43, 133.37, 132.20, 130.10, 129.99, 127.87, 123.19, 122.19, 119.73, 119.11, 80.89, 52.60, 28.44. MS (ESI) *m/z*: 356.2 $[M+H]^+$. HRMS (ESI) Calcd. for $C_{19}H_{21}N_3NaO_4$ $[M+Na]^+$ 378.1424, Found: 378.1423.

**Compound 1b**: Compound **2**[1] (8.13 g, 49.2 mmol) and **4**[3] (7.48 g, 33.1 mmol) were dissolved in a ternary solvent of AcOH/toluene/TFA (v/v/v = 6/6/1) (195 mL), and the resulting mixture was then stirred at room temperature for 3 days. After TLC suggesting the completion of the reaction, the reaction solution was turned to neutral by using saturated $NaHCO_3$ (a.q.). The resulting mixture was extracted with 400 mL $CH_2Cl_2$, and the organic phase was then isolated and dried over anhydrous $Na_2SO_4$. After removing the desiccant by filtrating, the solvent was further evaporated and the residue was purified with flash column chromatography by using a binary solvent of PE/EtOAc = 30/1 as the eluent. Compound **1b** could be obtained as orange solid (3.80 g, 31%). $^1$H NMR (600 MHz, $CDCl_3$, 298 K) δ: 11.18 (s, 1H), 8.28 (d, $J$ = 9.0 Hz, 1H), 7.97 (dd, $J_1$ = 7.8 Hz, $J_2$ = 1.2 Hz, 1H), 7.68 (dd, $J_1$ = 7.8 Hz, $J_2$ = 1.2 Hz, 1H), 7.62 (td, $J_1$ = 7.8 Hz, $J_2$ = 1.2 Hz, 1H), 7.52 (td, $J_1$ =7.8 Hz, $J_2$ = 1.2 Hz, 1H), 7.40 (td, $J_1$ = 9.6 Hz, $J_2$ = 6.0 Hz, 1H), 6.89 (ddd, $J_1$ = 11.4 Hz , $J_2$ = 7.8 Hz, $J_2$ = 0.6 Hz, 1H), 3.95 (s, 3H), 1.55 (s, 9H). $^{13}$C NMR (150 MHz, $CDCl_3$, 298 K) δ: 167.13, 162.66, 160.95, 153.53, 151.58, 134.24, 134.17, 133.73, 132.83, 130.75, 130.43, 128.05, 128.00, 127.84, 118.34, 114.91, 114.89, 109.13, 109.00, 81.12, 52.65, 28.41. $^{19}$F NMR (564 MHz, $CDCl_3$, 298 K) δ: -118.50. MS (ESI) *m/z*: 374.1 $[M+H]^+$. HRMS (ESI) Calcd. for $C_{19}H_{21}FN_3O_4$ $[M+H]^+$ 374.1511, Found: 374.1514.

**Compound 1c**: Compound **5**[4] (0.996 g, 5.89 mmol) was dissolved in 20 mL $CH_2Cl_2$, to which another 20 mL aqueous solution of Oxone (7.23 g, 11.8 mmol) was further added, and the resulting mixture was then stirred at room temperature for 48 hours. After the reaction was completed, the reaction mixture was consecutively washed by 30 mL of HCl (a.q., 1.0 mol/L), 30 mL of saturated $NaHCO_3$ (a.q.) and 30 mL of brine, respectively. The organic phase was collected and dried over anhydrous $Na_2SO_4$, which was further removed by filtration. After concentrating the filtrate by depressed evaporation, the crude compound **6** could be obtained as brown solid and was further added to a 26 mL of ternary solution of AcOH/toluene/TFA (v/v/v) = 6:6:1 in which compound **3**[2] (1.23g, 5.89 mmol) was dissolved. The resulting mixture was then stirred at room temperature for another 45 hours. When the TLC suggested the complete consumption of starting material, 100 mL of water was added to quench the reaction. The resulting mixture was extracted by $CH_2Cl_2$ (3 × 50 mL), and the organic phase was isolated and washed by water (3 × 50 mL) and brine (30 mL) subsequently, after which it was dried by anhydrous $Na_2SO_4$. The desiccant and solvent were discarded by filtration and evaporation under reduced pressure, respectively. Then, the residue was further purified by flash column chromatography eluted by a binary solvent of PE/EtOAc = 30/1, after which compound **1c** was obtained as orange solid (0.176 g, 8%). $^1$H

NMR (600 MHz, $CDCl_3$, 298 K) δ: 8.98 (s, 1H), 8.46 (d, $J$ = 8.4 Hz, 1H), 7.81 (dd, $J_1$ = 7.8 Hz, $J_2$ = 1.2 Hz, 1H), 7.50 (td, $J_1$ = 7.2 Hz, $J_2$ = 1.8 Hz, 1H), 7.47-7.43 (m, 2H), 7.40-7.36 (m, 1H), 7.10 (td, $J_1$ = 7.8 Hz, $J_2$ = 1.2 Hz, 1H), 3.82 (s, 3H), 1.58 (s, 9H). $^{13}C$ NMR (150 MHz, $CDCl_3$, 298 K) δ: 168.15, 168.13, 157.80, 156.10, 152.61, 140.34, 140.28, 139.07, 137.72, 134.20, 130.63, 130.57, 126.12, 124.97, 124.95, 122.18, 120.44, 119.49, 119.36, 118.99, 80.92, 52.61, 28.40. $^{19}F$ NMR (564 MHz, $CDCl_3$, 298 K) δ: -123.04. MS (ESI) *m/z*: 374.2 $[M+H]^+$. HRMS (ESI) Calcd. for $C_{19}H_{21}FN_3O_4$ $[M+H]^+$ 374.1511, Found: 374.1510.

**Compound 1d**: After dissolving compound **5**[4] (0.507 g, 3.00 mmol) in $CH_2Cl_2$ (15 mL), to which another 15 mL of aqueous solution containing Oxone (3.68 g, 6.00 mmol) was added, and the resulting mixture was stirred at room temperature for 48 hours. When the TLC suggested the reaction was completed, the reaction mixture was then washed continuously by 30 mL of HCl (a.q., 1.0 mol/L), 30 mL of saturated $NaHCO_3$ (a.q.) and 30 mL of brine, respectively. The organic phase was collected and dried over anhydrous $Na_2SO_4$, which was then discarded by filtration. After concentrating the filtrate via depressed evaporation, compound **6** could be obtained as brown solid which was further used without purification. After dissolving compound **4**[3] (0.679 g, 3.00 mmol) in the ternary solvent of AcOH/toluene/TFA (v/v/v) = 6:6:1, the crude compound **6** was further added. The resulting mixture was then stirred for another 44 hours until the reaction was completed. The reaction was then quenched by adding 60 mL of water and then extracted by $CH_2Cl_2$ (3 × 30 mL). The collected organic phase was then washed by water (3 × 30 mL) and brine (30 mL) subsequently and dried by using anhydrous $Na_2SO_4$ as desiccant, which was further discarded by filtration. The filtrated was concentrated by evaporation under reduced pressure, and the residue was further purified by flash column chromatography using a binary solvent of PE/EtOAc = 30/1 as eluent. Compound **1d** could be obtained as orange solid (0.153 g, 13%). $^1H$ NMR (600 MHz, $CDCl_3$, 298 K) δ: 11.21 (s, 1H), 8.29 (d, $J$ = 8.4 Hz, 1H), 7.48-7.39 (m, 3H), 7.38-7.34 (m, 1H), 6.86 (ddd, $J_1$ = 10.2 Hz, $J_2$ = 7.8 Hz, $J_3$ = 0.6 Hz, 1H), 3.84 (s, 1H), 1.53 (s, 9H). $^{13}C$ NMR (150 MHz, $CDCl_3$, 298 K) δ: 167.78, 167.76, 162.37, 160.65, 158.51, 156.80, 153.18, 139.24, 139.18, 135.05, 134.98, 134.37, 131.32, 131.26, 127.59, 127.55, 125.73, 125.12, 125.10, 119.19, 119.06, 114.42, 114.40, 108.97, 108.83, 81.11, 52.62, 52.60, 28.29. $^{19}F$ NMR (564 MHz, $CDCl_3$, 298 K) δ: -118.07, -122.69. MS (ESI) *m/z*: 392.2 $[M+H]^+$. HRMS (ESI) Calcd. for $C_{19}H_{19}F_2KN_3O_4$ $[M+K]^+$ 430.0975, Found: 430.0982.

**Compound 7**: After dissolving compound **1b** (0.322 g, 0.863 mmol) in 2.6 mL of $CH_2Cl_2$ and the solution was cooled in ice bath. Then, 0.26 mL of trifluoroacetic acid (TFA) was added slowly and the resulting mixture was further stirred for another 18 hours under the ice bath. When the reaction was completed, the solvent was removed by evaporation to generate orange-red solid, which was further redissolved in EtOAc

(20 mL), and continuously washed by saturated $NaHCO_3$ solution (a.q. 30 mL), water (30 mL) and brine (30 mL), respectively. The organic phase was collected and dried with anhydrous $Na_2SO_4$, which was further removed by filtration. After concentrating the filtrate by evaporation under reduced pressure, compound **7** could be obtained as orange-red solid (0.233 g, 99%). $^1H$ NMR (600 MHz, $CDCl_3$, 298 K) δ: 7.98 (dd, $J_1$ = 7.2 Hz, $J_2$ = 1.2 Hz, 1H), 7.92 (dd, $J_1$ = 8.4 Hz, $J_2$ = 1.2 Hz, 1H), 7.60 (ddd, $J_1$ = 9.6 Hz, $J_2$ = 7.2 Hz, $J_3$ = 1.2 Hz, 1H), 7.46-7.43 (m, 2H), 6.52-6.46 (m, 2H), 3.92 (s, 3H). $^{13}C$ NMR (150 MHz, $CDCl_3$, 298 K) δ: 167.33, 163.65, 161.96, 151.57, 141.82, 141.81, 133.38, 133.30, 132.89, 130.80, 129.45, 127.37, 126.67, 126.62, 116.55, 112.80, 112.78, 102.58, 102.44, 52.36. $^{19}F$ NMR (564 MHz, $CDCl_3$, 298 K) δ: -120.36. MS (ESI) *m/z*: 274.1 $[M+H]^+$. HRMS (ESI) Calcd. for $C_{14}H_{12}FN_3NaO_3$ $[M+Na]^+$ 296.0806, Found: 296.0813.

**Azo-UPy**: Compound **7** (0.234 g, 0.856 mmol) and compound **8**[5] (0.607 g, 1.71 mmol) were dissolved in chroloform (3.0 mL), 25 μL of $Et_3N$ was further added and the resulting mixture was sitrred at 50 °C for another 17 hours. After the starting material was fully consumed as suggested by TLC, 20 mL of $CH_2Cl_2$ was added to quench the reaction. The mixture was then washed continously 20 mL of HCl (a.q., 1.0 mol/L), saturated $NaHCO_3$ (a.q., 30 mL) and brine (30 mL), respectively. The organic phase was then collected and dried by anhydrous $Na_2SO_4$, which was removed by filtration. After removing the solvent by evaporation under reduced pressure, the residue was further purified by flash column chromatography using a binary solvent of DCM/MeOH = 30/1 as eluent. Compound **Azo-UPy** could be isolated as orange solid (0.301 g, 69%). $^1H$ NMR (600 MHz, $CDCl_3$, 298 K) δ: 12.15 (s, 1H), 11.81 (s, 1H), 9.89 (s, 1H), 8.51 (d, $J$ = 8.4 Hz, 1H), 8.22 (dd, $J_1$ = 7.8 Hz, $J_2$ = 1.2 Hz, 1H), 8.01 (dd, $J_1$ = 8.4 Hz, $J_2$ = 1.2 Hz, 1H), 7.74 (td, $J_1$ = 7.2 Hz, $J_2$ = 1.2 Hz, 1H), 7.62 (td, $J_1$ = 7.8 Hz, $J_2$ = 1.2 Hz, 1H), 7.49 (td, $J_1$ = 8.4 Hz, $J_2$ = 6.0 Hz ,1H), 7.00 (t, $J$ = 9.0 Hz, 1H), 5.91 (s, 1H), 4.21 (s, 3H), 2.21-2.16 (m, 1H), 1.55-1.42 (m, 4H), 1.33-1.12 (m, 4H), 0.85 (t, $J$ = 7.2 Hz, 3H), 0.81 (t, $J$ = 7.2 Hz, 3H). $^{13}C$ NMR (150 MHz, $CDCl_3$, 298 K) δ: 171.83, 167.45, 162.81, 161.94, 161.09, 153.59, 151.12, 150.83, 134.70, 134.63, 134.27, 131.67, 131.60, 131.27, 128.38, 128.34, 125.33, 116.20, 115.50, 115.48, 110.19, 110.05, 107.58, 53.89, 49.12, 33.56, 29.68, 26.99, 22.81, 14.06, 12.05. $^{19}F$ NMR (564 MHz, $CDCl_3$, 298 K) δ: -117.35. MS (ESI) *m/z*: 509.2 $[M+H]^+$. HRMS (ESI) Calcd. for $C_{26}H_{30}FN_6O_4$ $[M+H]^+$ 509.2307, Found: 509.2308.

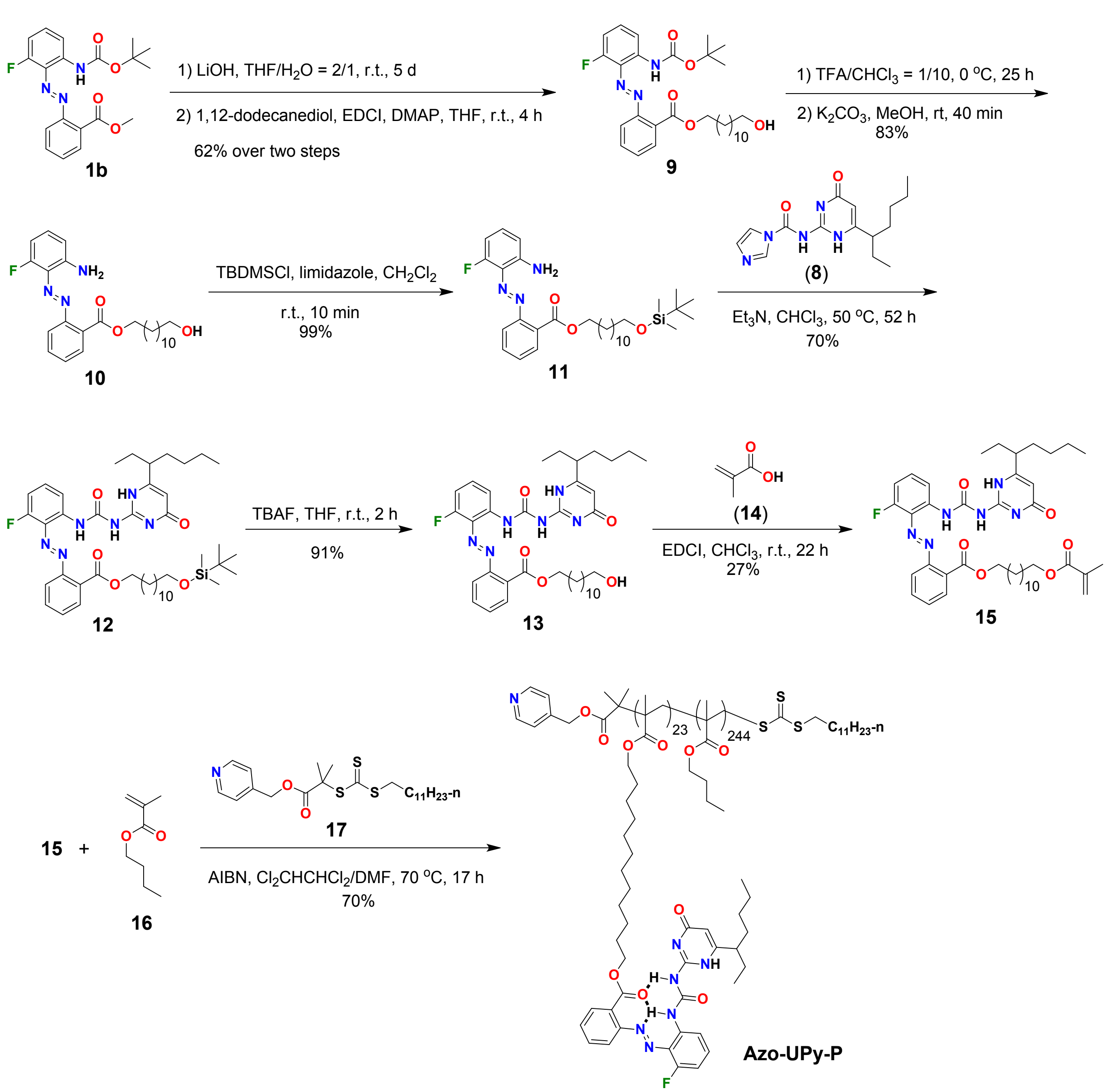

**Scheme S2.** The synthetic rout for the **Azo-UPy** modified polymer of **Azo-UPy-P**.

**Compound 9**: Compound **1b** (4.48 g, 12.0 mmol) was dissolved in 200 mL of THF, to which another 100 mL of aqueous solution containing LiOH (11.2 g, 468 mmol) was added. The resulting mixture was then stirred at room temperature for 5 days, until TLC suggested the reaction was completed. The pH value of the reaction solution was tuned to pH = 2 ~ 3 by using HCl (a.q., 1.0 mol/L), and then 200 mL of ethyl acetate was added. The organic phase of the resulting mixture was continuously washed by water (200 mL) and brine (150 mL), and further dried with anhydrous $Na_2SO_4$. The desiccant was then removed by filtration, and the solvent was discarded by evaporation under reduced pressure to give the crude (carboxylic acid) intermediate compound as orange solid. Without purification, this crude intermediate compound was directly added to the anhydrous THF solution (30 mL) containing 1,12-dodecanediol (12.1 g, 59.9 mmol), to which the EDCI (2.52 g, 13.2 mmol) and DMAP (0.249 g, 2.04 mmol) were further

added, and the resulting mixture was then stirred at room temperature for 4 hours. After the reaction was completed as indicated by the TLC, the solvent was removed by evaporation under reduced pressure. The obtained yellow solid was redissolved in 30 mL of $CH_2Cl_2$ and the remaining unreacted 1,12-dodecanediol was removed by filtration. The organic filtrate was then continuously washed by 30 mL of HCl (a.q., 1.0 mM), 30 mL of saturated $NaHCO_3$ (a.q.) and 30 mL of brine, respectively, and then dried by anhydrous $Na_2SO_4$. After removing the desiccant and solvent by filtration and evaporation, respectively, the residue was further purified by flash column chromatography with a binary eluent of DCM/EtOAc = 30/1. Compound **9** was finally isolated as orange solid (4.04 g, 62%). $^1$H NMR (600 MHz, $CD_3COCD_3$, 298 K) δ: 11.13 (s, 1H), 8.36 (d, $J$ = 8.4 Hz, 1H), 7.99 (dd, $J_1$ = 7.8 Hz, $J_2$ = 1.2 Hz, 1H), 7.88-7.75 (m, 1H), 7.69-7.66 (m, 2H), 7.57 (td, $J_1$ = 8.4 Hz, $J_2$ = 6.0 Hz, 1H), 7.05 (ddd, $J_1$ = 9.6 Hz, $J_2$ = 8.4 Hz, $J_3$ = 1.2 Hz, 1H), 4.39 (t, $J$ = 6.6 Hz, 2H), 3.53 (t, $J$ = 6.0 Hz, 2H), 3.40 (br, 1H), 1.72 (quintet, $J$ = 7.8 Hz, 2H), 1.51 (quintet, $J$ = 7.8 Hz, 2H), 1.37-1.32 (m, 4H), 1.30-1.16 (m, 12H). $^{13}$C NMR (150 MHz, $CDCl_3$, 298 K) δ: 166.81, 162.54, 160.83, 153.48, 151.65, 134.20, 134.13, 133.80, 132.61, 130.53, 130.36, 128.30, 127.95, 127.90, 118.43, 114.80, 114.77, 109.10, 108.96, 81.08, 65.90, 63.12, 32.89, 29.66, 29.61, 29.60, 29.55, 29.52, 29.37, 28.69, VB28.40, 26.18, 25.84. $^{19}$F NMR (564 MHz, $CD_3COCD_3$, 298 K) δ: -119.70. MS (ESI) *m/z*: 544.3 $[M+H]^+$. HRMS (ESI) Calcd. for $C_{30}H_{43}FN_3O_5$ $[M+H]^+$ 544.3181, Found: 544.3183.

**Compound 10**: Compound **9** (4.00 g, 7.36 mmol) was dissolved in 42 mL of $CH_2Cl_2$ and cooled in an ice bath, to the solution of which 4.2 mL of $CF_3COOH$ was then added slowly, and the resulting mixture was further stirred for another 25 hours while keeping in the ice both. After TLC suggested the totally consuming of the starting materials, the reaction mixture was concentrated under reduced pressure to remove the solvent. The obtained orange-red solid was redissolved in 40 mL of methanol, to which anhydrous $K_2CO_3$ (1.76 g, 12.8 mmol) was further added. The resulting mixture was then stirred at room temperature for another 45 min, after which 100 mL of ethyl acetate was added and the insoluble solid was removed by filtration. The obtained filtrate was then washed by water (60 mL × 3) and brine (30 mL), respectively. The organic phase was collected and dried by anhydrous $Na_2SO_4$, which was further removed by filtration. After the solvent was evaporated under reduced pressure, the residue was further purified by flash column chromatography with a binary solvent of PE/EtOAc = 6/1 as the eluent. Compound **10** could be isolated as orange solid (2.71 g, 83%). $^1$H NMR (600 MHz, $CDCl_3$, 298 K) δ: 7.96 (d, $J$ = 7.8 Hz, 1H), 7.90 (d, $J$ = 8.4 Hz, 1H), 7.59 (t, $J$ = 7.8 Hz, 1H), 7.45 (t, $J$ = 7.2 Hz, 1H), 7.12 (td, $J_1$ = 8.4 Hz, $J_2$ = 6.0 Hz, 1H), 6.50-6.45 (m, 2H), 4.31 (t, $J$ = 7.2 Hz, 2H), 3.61 (t, $J$ = 6.6 Hz, 2H), 1.75 (quintet, $J$ = 7.2 Hz, 2H), 1.54 (quintet, $J$ = 7.2 Hz, 2H), 1.40 (quintet, $J$ = 7.2 Hz, 2H), 1.34-1.23 (m, 14H). $^{13}$C NMR (150 MHz, $CDCl_3$, 298 K) δ: 167.00, 163.70, 162.00, 151.69, 141.90, 141.90, 133.36, 133.28, 132.77, 130.67, 129.44, 127.84, 126.74, 126.69, 116.60, 112.85, 112.82, 102.58, 102.44, 65.61,

63.09, 32.86, 29.66, 29.62, 29.58, 29.50, 29.36, 28.75, 26.13, 25.83. $^{19}$F NMR (564 MHz, $CDCl_3$, 298 K) δ: -120.41. MS (ESI) *m/z*: 444.3 $[M+H]^+$. HRMS (ESI) Calcd. for $C_{25}H_{34}FN_3NaO_3$ $[M+Na]^+$ 466.2476, Found: 466.2474.

**Compound 11**: Compound **10** (2.69 g, 6.07 mmol), TBSCl (1.08 g, 7.19 mmol) and imidazole (0.586 g, 8.62 mmol) were dissolved in 25 mL of $CH_2Cl_2$, the resulting mixture was then stirred at room temperature for 10 min. After the starting material was fully consumed as detected by TLC, additional 75 mL of $CH_2Cl_2$ was then add to the reaction mixture, which was then washed by water (100 mL × 3) and brine (30 mL), respectively. The organic phase was collected and dried by anhydrous $Na_2SO_4$. After removing the desiccant and solvent by filtration and evaporation, respectively. The residue was further purified by flash column chromatography with a binary solvent of PE/EtOAc = 15/1 as the eluent. Compound **11** could be isolated as orange oil (3.35 g, 99%). $^1$H NMR (600 MHz, $CDCl_3$, 298 K) δ: 8.00 (dd, $J_1$ = 7.8 Hz, $J_2$ = 1.2 Hz, 1H), 7.94 (dd, $J_1$ = 8.4 Hz, $J_2$ = 1.2 Hz, 1H), 7.63 (ddd, $J_1$ = 9.6 Hz, $J_2$ = 7.2 Hz, $J_3$ = 1.2 Hz, 1H), 7.48 (td, $J_1$ = 7.2 Hz, $J_2$ = 1.2 Hz, 1H), 7.16 (td, $J_1$ = 7.8 Hz, $J_2$ = 6.0 Hz, 1H), 6.54-6.49 (m, 2H), 4.35 (t, $J$ = 6.6 Hz, 2H), 3.62 (t, $J$ = 6.6 Hz, 2H), 1.78 (quintet, $J$ = 7.2 Hz, 2H), 1.53 (quintet, $J$ = 7.2 Hz, 2H), 1.44 (quintet, $J$ = 7.2 Hz, 2H), 1.37-1.27 (m, 14H), 0.92 (s, 9H), 0.08 (s, 6H). $^{13}$C NMR (150 MHz, $CDCl_3$, 298 K) δ: 166.99, 163.75, 162.06, 151.74, 141.89, 133.39, 133.31, 132.81, 130.72, 129.49, 127.89, 126.81, 126.76, 116.61, 112.86, 112.84, 102.65, 102.51, 65.64, 63.47, 33.03, 29.76, 29.71, 29.70, 29.65, 29.58, 29.43, 28.81, 26.19, 26.13, 25.94, 18.52, -5.11. $^{19}$F NMR (564 MHz, $CDCl_3$, 298 K) δ: -120.39. MS (ESI) *m/z*: 558.4 $[M+H]^+$. HRMS (ESI) Calcd. for $C_{31}H_{48}FN_3NaO_3Si$ $[M+Na]^+$ 580.3341, Found: 580.3345.

**Compound 12**: Compound **11** (3.29 g, 5.90 mmol) and compound **8**[5] (3.56 g, 10.0 mmol) were dissolved in 10 mL of chloroform, to which 0.32 mL of $Et_3N$ was added, and the resulting mixture was heated at 50 °C with stirring for 52 hours. After the reaction was completed as suggested by TLC, 100 mL of $CH_2Cl_2$ was further added to quench the reaction, and the mixture was then continuously washed by saturated $NaHCO_3$ (a.q., 60 mL), diluted HCl (a.q., 1.0 mol/L, 60 mL) and brine (60 mL), respectively. The organic phased was collected and dried by anhydrous $Na_2SO_4$. After removing the desiccant and solvent by filtration and evaporation, respectively, the residue was further purified by flash column chromatography with a binary eluent of PE/EtOAc = 6/1. Compound **12** was isolated as orange solid (3.28 g, 70%). $^1$H NMR (600 MHz, $CDCl_3$, 298 K) δ: 11.97 (s, 1H), 11.79 (s, 1H), 9.80 (s, 1H), 8.46 (d, $J$ = 8.4 Hz, 1H), 8.16 (dd, $J_1$ = 7.8 Hz, $J_2$ = 1.2 Hz, 1H), 7.93 (d, $J$ = 8.4 Hz, 1H), 7.67 (ddd, $J_1$ = 8.4 Hz, $J_2$ = 7.8 Hz, $J_3$ = 1.2 Hz, 1H), 7.58 (td, $J_1$ = 7.8 Hz, $J_2$ = 1.2 Hz, 1H), 7.41 (td, $J_1$ = 8.4 Hz, $J_2$ = 6.0 Hz, 1H), 6.92 (t, $J$ = 9.0 Hz, 1H), 5.93 (s, 1H), 4.59 (t, $J$ = 6.0 Hz, 2H), 3.60 (t, $J$ = 6.6 Hz, 2H), 2.25-2.19 (m, 1H), 1.89-1.83 (m, 2H), 1.62-1.49 (m, 8H), 1.44-1.39 (m, 2H), 1.36-1.26 (m, 14H), 1.23-1.19 (m, 2H), 0.90-

0.87 (m, 12H), 0.85 (t, $J$ = 7.8 Hz, 3H), 0.05 (s, 6H). $^{13}C$ NMR (150 MHz, $CDCl_3$, 298 K) δ: 171.92, 167.06, 162.86, 162.05, 161.15, 153.81, 151.19, 150.98, 134.67, 134.60, 134.17, 131.75, 131.64, 131.35, 128.63, 128.59, 125.95, 116.30, 115.74, 115.72, 110.28, 110.15, 107.57, 67.25, 63.39, 49.22, 33.61, 32.98, 29.77, 29.74, 29.71, 29.66, 29.55, 29.50, 28.73, 27.05, 26.55, 26.08, 25.91, 22.91, 18.46, 14.16, 12.10, -5.16. $^{19}F$ NMR (564 MHz, $CDCl_3$, 298 K) δ: -117.22. MS (ESI) *m/z*: 793.5 $[M+H]^+$. HRMS (ESI) Calcd. for $C_{43}H_{66}FN_6O_5Si$ $[M+H]^+$ 793.4843, Found: 793.4850.

**Compound 13**: Compound **12** (0.801 g, 1.01 mmol) was dissolved in THF containing TBAF (1.0 mol/L), and the resulting mixture was then stirred at room temperature for 2 hours until the total consumption of starting material as detected by TLC. The solvent of the reaction mixture was then removed by evaporation under reduced pressure, and the obtained viscous oil residue was reprecipitated by adding 5 mL of water. The generated orange precipitate was then collected by filtration and further purified by flash column chromatography with a binary eluent of DCM/EtOAc = 1/1. Compound **13** was isolated as orange solid (0.624 g, 91%). $^1H$ NMR (600 MHz, $CDCl_3$, 298 K) δ: 12.00 (s, 1H), 11.81 (s, 1H), 9.83 (s, 1H), 8.46 (d, $J$ = 9.0 Hz, 1H), 8.17 (dd, $J_1$ = 7.8 Hz, $J_2$ = 1.2 Hz, 1H), 7.94 (d, $J$ = 7.8 Hz, 1H), 7.68 (ddd, $J_1$ = 8.4 Hz, $J_2$ = 7.8 Hz, $J_3$ = 1.2 Hz, 1H), 7.59 (td, $J_1$ = 7.8 Hz, $J_2$ = 1.2 Hz, 1H), 7.43 (td, $J_1$ = 8.4 Hz, $J_2$ = 6.0 Hz, 1H), 6.94 (t, $J$ = 9.0 Hz, 1H), 5.93 (s, 1H), 4.60 (t, $J$ = 6.0 Hz, 2H), 3.64 (t, $J$ = 6.6 Hz, 2H), 2.25-2.19 (m, 1H), 1.88-1.83 (m, 2H), 1.61-1.47 (m, 8H), 1.42-1.38 (m, 2H), 1.36-1.25 (m, 14H), 1.22-1.17 (m, 2H), 0.88 (t, $J$ = 7.2 Hz, 3H), 0.84 (t, $J$ = 7.2 Hz, 3H). $^{13}C$ NMR (150 MHz, $CDCl_3$, 298 K) δ: 172.06, 167.11, 162.86, 162.15, 161.15, 153.83, 151.20, 150.97, 134.68, 134.62, 134.19, 131.74, 131.63, 131.37, 128.67, 128.62, 126.00, 116.31, 115.77, 115.74, 110.32, 110.18, 107.52, 67.21, 63.01, 49.22, 33.60, 32.88, 29.76, 29.70, 29.66, 29.62, 29.53, 29.46, 28.73, 27.04, 26.50, 25.86, 22.90, 14.15, 12.09. $^{19}F$ NMR (564 MHz, $CDCl_3$, 298 K) δ: -117.27. MS (ESI) *m/z*: 701.4 $[M+Na]^+$. HRMS (ESI) Calcd. for $C_{37}H_{52}FN_6O_5$ $[M+H]^+$ 679.3978, Found: 679.3991.

**Compound 15**: Compound **13** (0.781 g, 1.15 mmol) was dissolved in 3.0 mL of chloroform, to the solution of which the methacrylic acid (**14**) (0.0989 g, 1.15 mmol), DMAP (0.070 g, 0.573 mmol) and EDCI (0.242 g, 1.26 mmol) were then added, and the resulting mixture was stirred at room temperature for 22 hours. 60 mL of $CH_2Cl_2$ was then added to quench the reaction, and the mixture was continuously washed by saturated $NaHCO_3$ (a.q., 60 mL), diluted HCl (a.q., 1.0 mol/L, 60 mL) and brine (60 mL), respectively. The organic phased was collected and dried by anhydrous $Na_2SO_4$. After removing the desiccant and solvent by filtration and evaporation, respectively, the residue was further purified by flash column chromatography with a binary eluent of DCM/EtOAc = 30/1. Compound **15** was obtained as yellow solid (0.232 g, 27%). $^1H$ NMR (600 MHz, $CDCl_3$, 298 K) δ: 12.02 (s, 1H), 11.81 (s, 1H), 9.83 (s, 1H), 8.48 (d, $J$ = 9.0 Hz, 1H), 8.18 (dd, $J_1$ = 7.8 Hz, $J_2$ = 1.2 Hz, 1H), 7.95 (d, $J$ = 7.8 Hz, 1H), 7.69 (ddd,

$J_1$ = 7.8 Hz, $J_2$ = 7.2 Hz, $J_3$ = 1.2 Hz, 1H), 7.60 (td, $J_1$ = 8.4 Hz, $J_2$ = 1.2 Hz, 1H), 7.43 (td, $J_1$ = 8.4 Hz, $J_2$ = 6.0 Hz, 1H), 6.95 (t, $J$ = 9.0 Hz, 1H), 6.10-6.09 (m, 1H), 5.93 (s, 1H), 5.55 (quintet, $J$ = 1.2 Hz, 1H), 4.61 (t, $J$ = 6.0 Hz, 1H), 4.14 (t, $J$ = 7.2 Hz, 1H), 2.25-2.19 (m, 1H), 1.95 (t, $J$ = 1.2 Hz, 2H), 1.89-1.84 (m, 2H), 1.69-1.65 (m, 2H), 1.61-1.49 (m, 6H), 1.43-1.27 (m, 16H), 1.23-1.17 (m, 2H), 0.88 (t, $J$ = 7.2 Hz, 3H), 0.85 (t, $J$ = 7.2 Hz, 3H). $^{13}$C NMR (150 MHz, $CDCl_3$, 298 K) δ: 171.94, 167.61, 167.12, 162.88, 162.08, 161.17, 153.84, 151.22, 150.99, 136.63, 134.70, 134.63, 134.21, 131.76, 131.64, 131.37, 128.68, 128.64, 126.01, 125.21, 116.34, 115.78, 115.76, 110.32, 110.19, 107.58, 67.23, 64.88, 49.22, 33.62, 29.76, 29.68, 29.65, 29.61, 29.49, 29.34, 28.75, 28.69, 27.06, 26.54, 26.06, 22.91, 18.42, 14.15, 12.09. $^{19}$F NMR (564 MHz, $CDCl_3$, 298 K) δ: -117.28. MS (ESI) *m/z*: 747.4 $[M+H]^+$. HRMS (ESI) Calcd. for $C_{41}H_{56}FN_6O_6$ $[M+H]^+$ 747.4240, Found: 747.4246.

**Azo-UPy-P.** Compound **15** (0.161 g, 0.216 mmol), butyl methacrylate (**16**) (0.307 g, 2.16 mmol) and compound **17**[6] (0.0100 g, 0.0220 mmol) were mixed with 0.36 mL of 1,1,1,2-tetrachloroethane in a glass tube, to which 40 μL of DMF containing AIBN (0.0020 g, 0.0120 mmol) was further added, and then the tube was sealed by a rubber cover with aluminum foil. After three freeze-pump-thaw cycles in liquid $N_2$ through a long needle, the pinhole on the rubber cover was sealed with paraffin. The sealed tube was then heated to 70 $^o$C with stirring for 17 hours, after which the viscous reaction mixture was then cooled down and transferred to 100 mL of acetone drop by drop. The generated viscous precipitates were collected and redissolved in 3.0 mL of chloroform, the solution of which was added to another 100 mL of acetone by droplets to generate precipitates again. After collecting the precipitates (as viscous liquid) and further dried under vacuum, the polymeric product of **Azo-UPy-P** could be obtained as orange-red viscous elastomer (0.328 g, 70%, $M_{n, NMR}$ = 51747 Da, $M_{n, GPC}$ = 58994 Da, $PDI_{GPC}$ = 1.40).

## Section 3: Photoswitching behavior of model compounds.

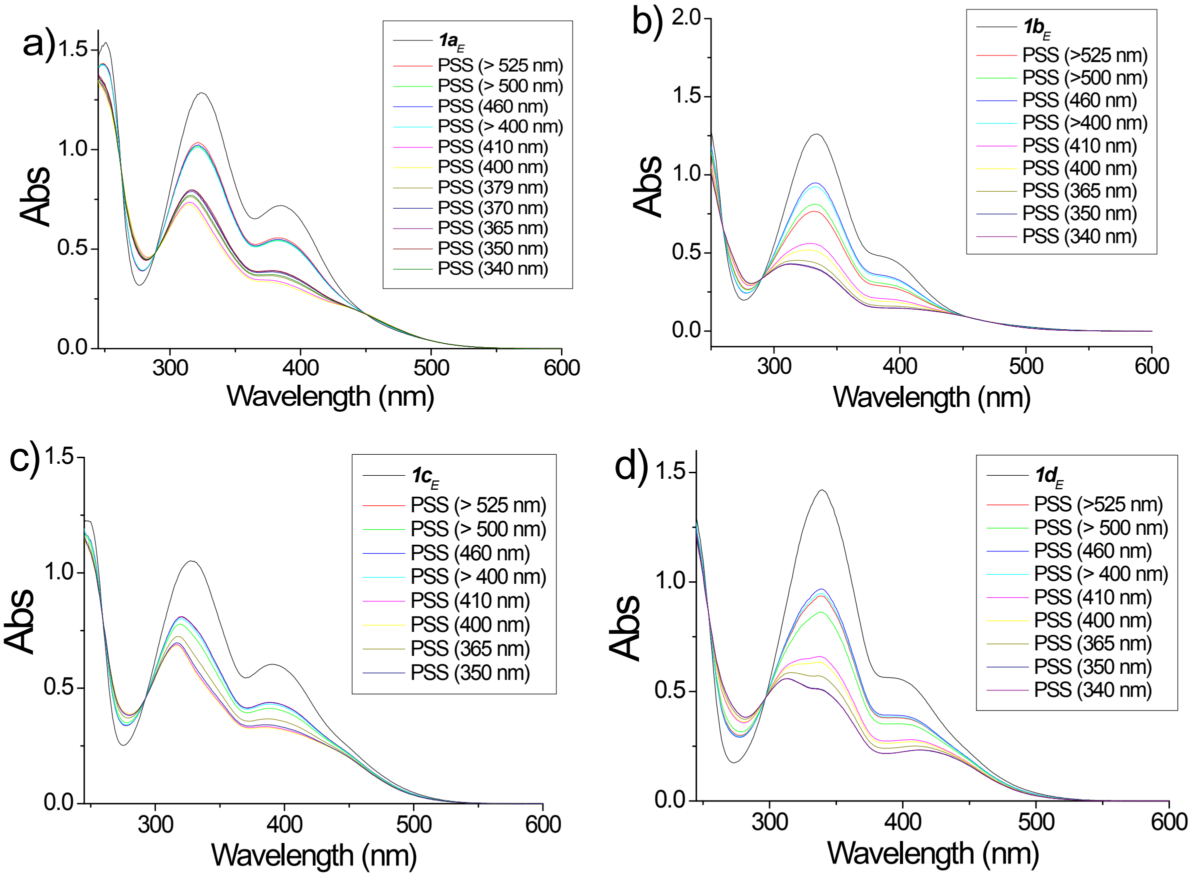


**Fig. S1.** UV-Vis absorption spectra of model compounds of a) **1a**, b) **1b**, c) **1c** and d) **1d** in their pristine states and at the PSSs after irradiation with various light sources recorded in $CHCl_3$ at 25 $^{o}C$. The concentration of the sample is 0.10 mM.

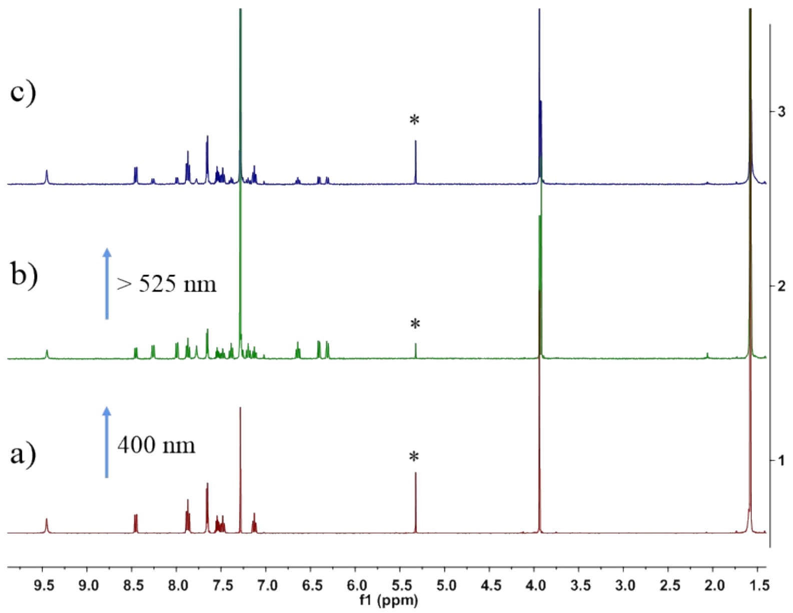


**Fig. S2.** $^{1}H$ NMR spectra (600 MHz, $CDCl_3$, 298K) of the solution of a) $\mathbf{1a}_E$ (10. mM), b) the $PSS_Z$ (400 nm) mixtures of **1a** (5.0 mM), and c) the $PSS_E$ (> 525 nm) mixtures of **1a** (5.0 mM). The "*" marked signals refer to $CH_2Cl_2$.

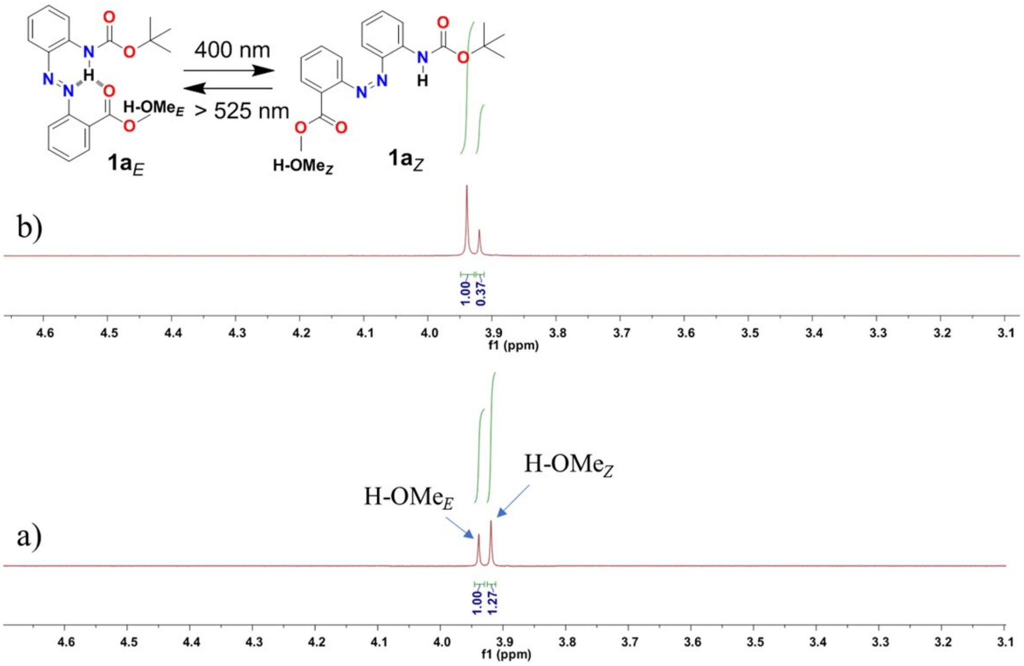


**Fig. S3.** Partial $^{1}$H NMR spectra (600 MHz, $CDCl_3$, 298K) of the solution of d) the PSS$_Z$ (400 nm) mixtures of **1a** (5.0 mM), and e) the PSS$_E$ (> 525 nm) mixtures of **1a** (5.0 mM).

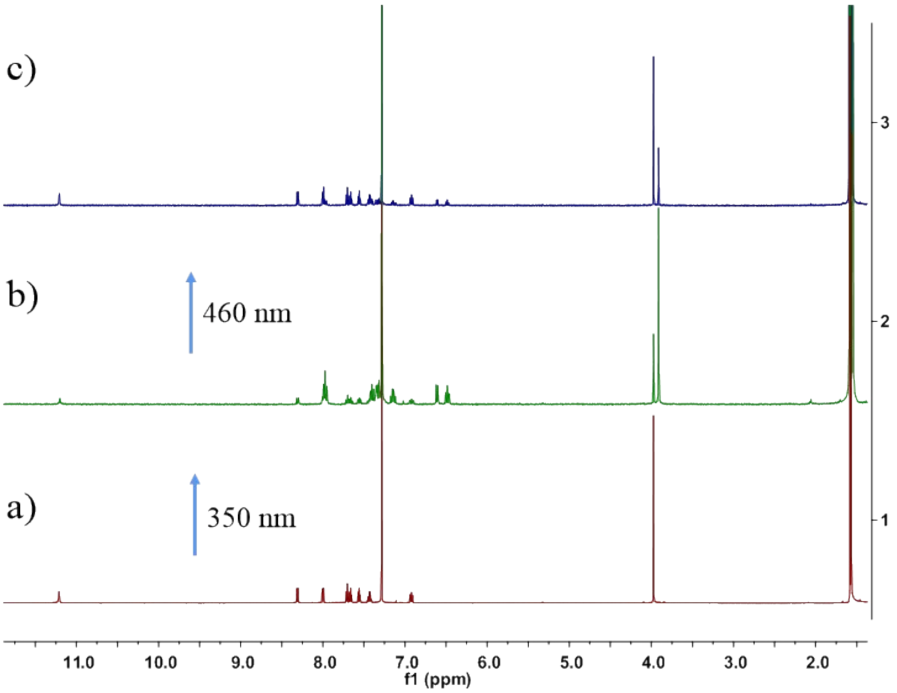


**Fig. S4.** $^{1}$H NMR spectra (600 MHz, $CDCl_3$, 5.0 mM, 298K) of the solution of a) **1b**$_E$, b) the PSS$_Z$ (350 nm) mixtures of **1b**, and c) the PSS$_E$ (460 nm) mixtures of **1b**.

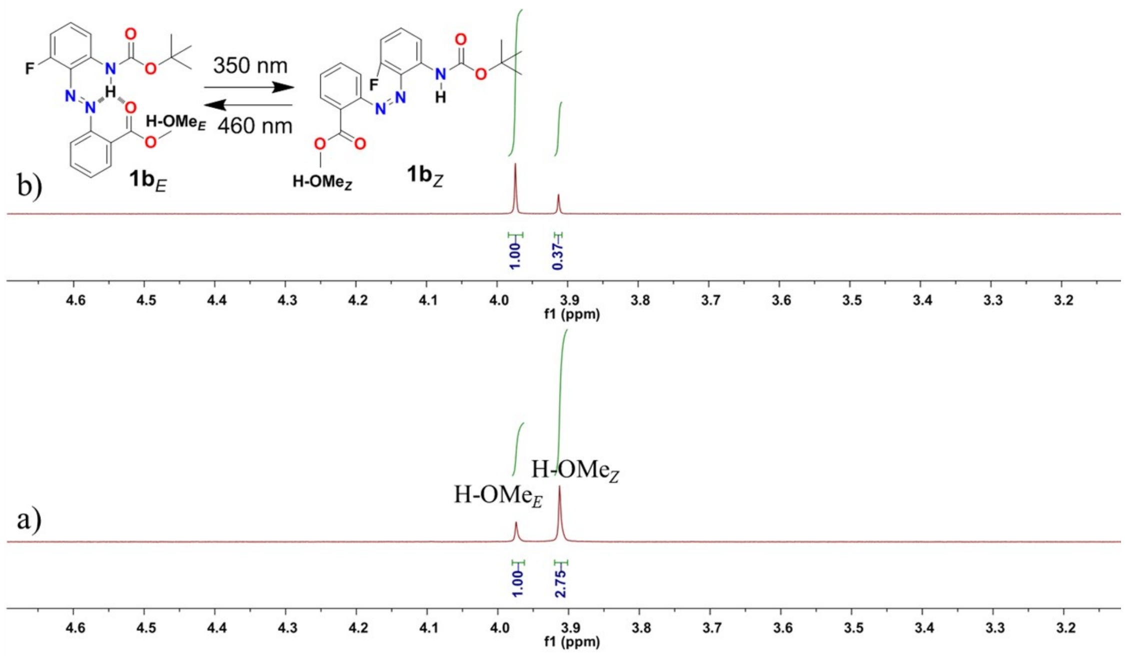


**Fig. S5.** Partial $^{1}$H NMR spectra (600 MHz, $CDCl_3$, 5.0 mM, 298K) of the solution of a) the PSS$_Z$ (350 nm) mixtures of **1b**, and b) the PSS$_E$ (460 nm) mixtures of **1b**.

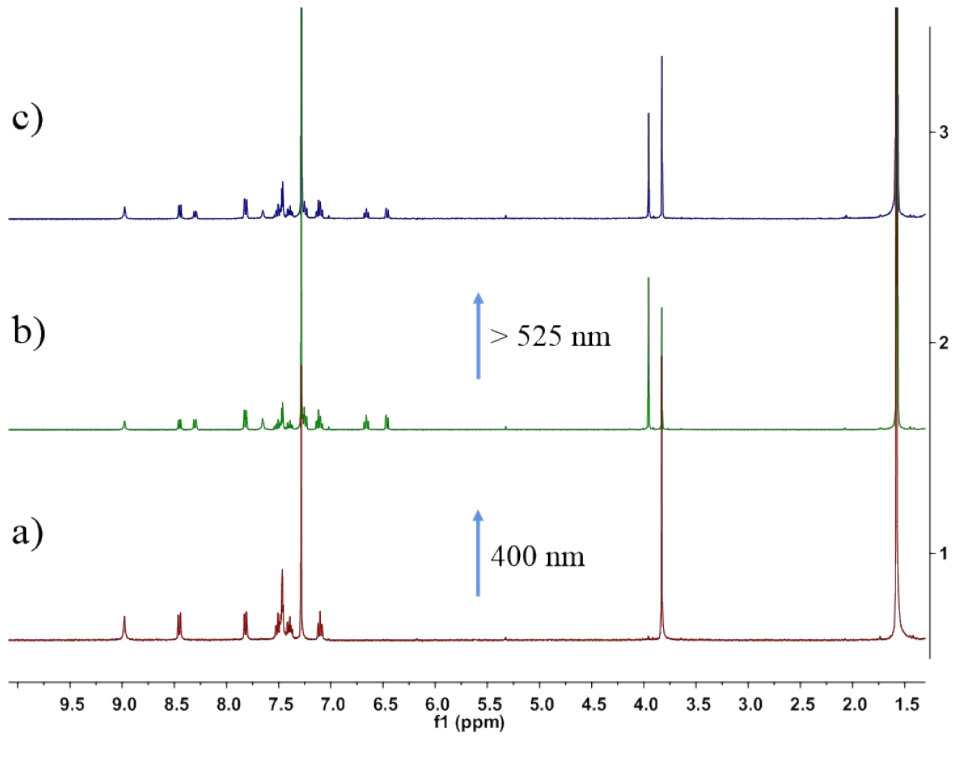


**Fig. S6.** $^{1}$H NMR spectra (600 MHz, $CDCl_3$, 5.0 mM, 298K) of the solution of a) **1c**$_E$, b) the PSS$_Z$ (400 nm) mixtures of **1c**, and c) the PSS$_E$ (> 525 nm) mixtures of **1c**.

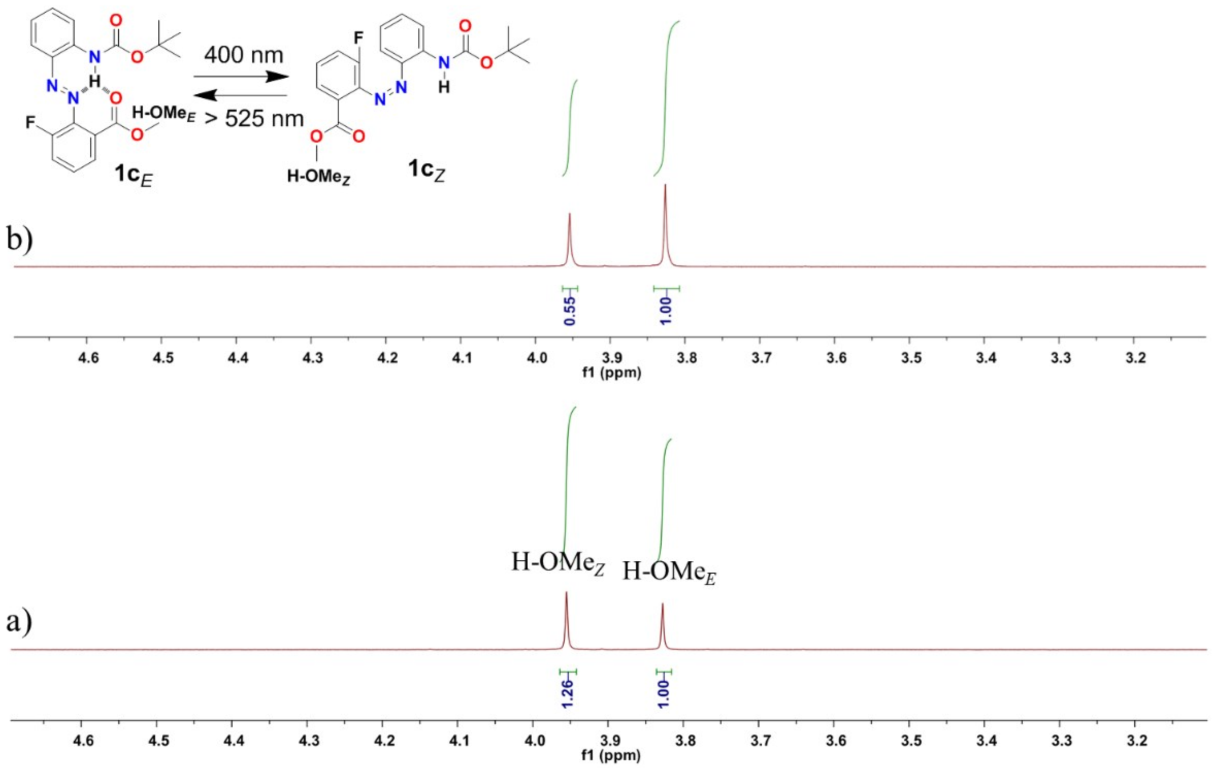


**Fig. S7.** Partial $^1$H NMR spectra (600 MHz, $CDCl_3$, 5.0 mM, 298K) of the solution of a) the PSS$_Z$ (400 nm) mixtures of **1c**, and b) the PSS$_E$ (> 525 nm) mixtures of **1c**.

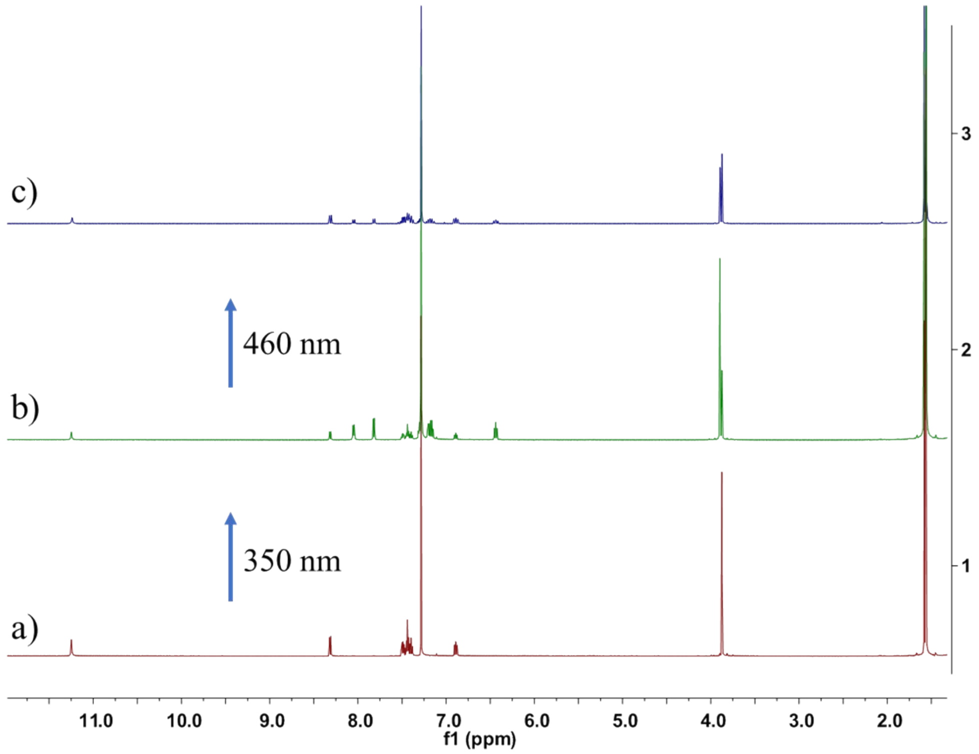


**Fig. S8.** $^1$H NMR spectra (600 MHz, $CDCl_3$, 5.0 mM, 298K) of the solution of a) **1d**$_E$, b) the PSS$_Z$ (350 nm) mixtures of **1d**, and c) the PSS$_E$ (460 nm) mixtures of **1d**.

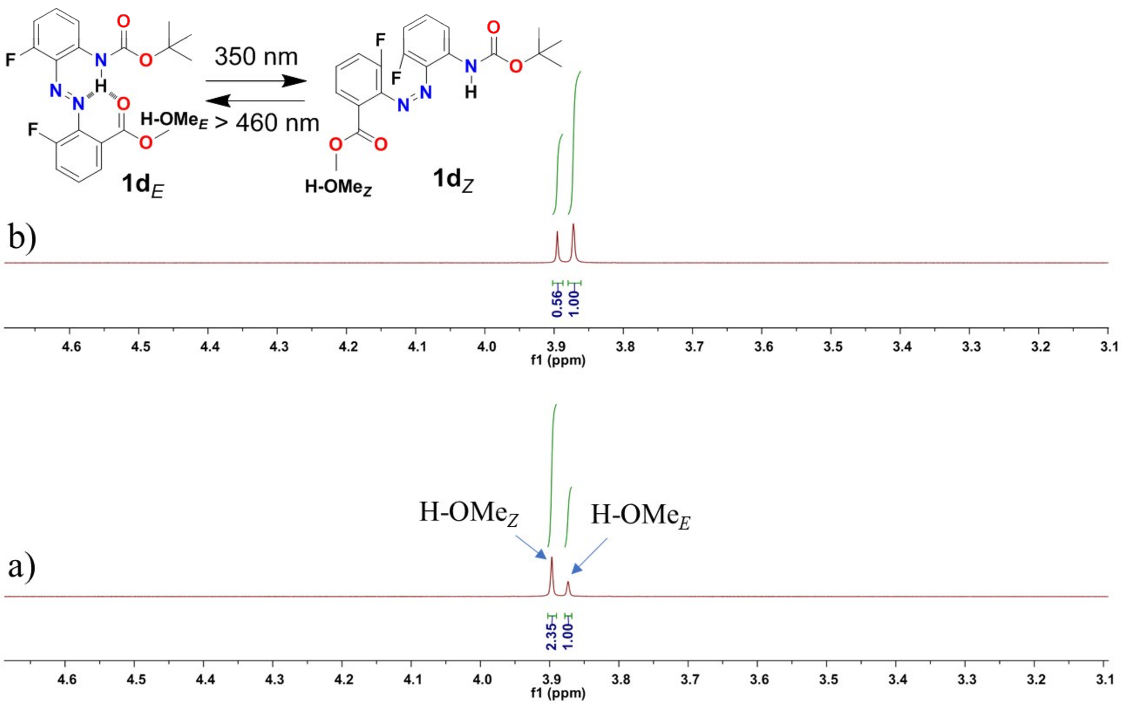


**Fig. S9.** Partial $^1$H NMR spectra (600 MHz, $CDCl_3$, 5.0 mM, 298K) of the solution of a) the PSS$_Z$ (350 nm) mixtures of **1d**, and b) the PSS$_E$ (460 nm) mixtures of **1d**.

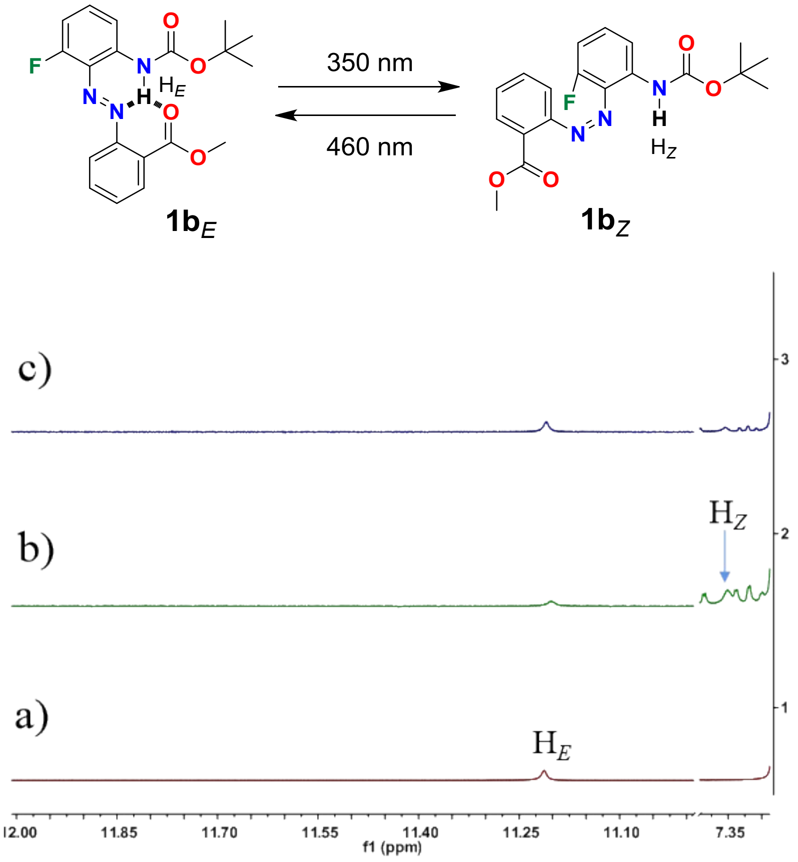


**Fig. S10.** The schematic representation of the photoisomerization behavior of **1b**, and the partial $^1$H NMR spectra (600 MHz, $CDCl_3$, 5.0 mM, 298K) of the solution of a) **1b**$_E$, b) the PSS$_Z$ (350 nm) mixtures of **1b**, and c) the PSS$_E$ (460 nm) mixtures of **1b**.

**Section 4: Photoswitchable quadruple H-bonded self-dimerization of Azo-UPy.**

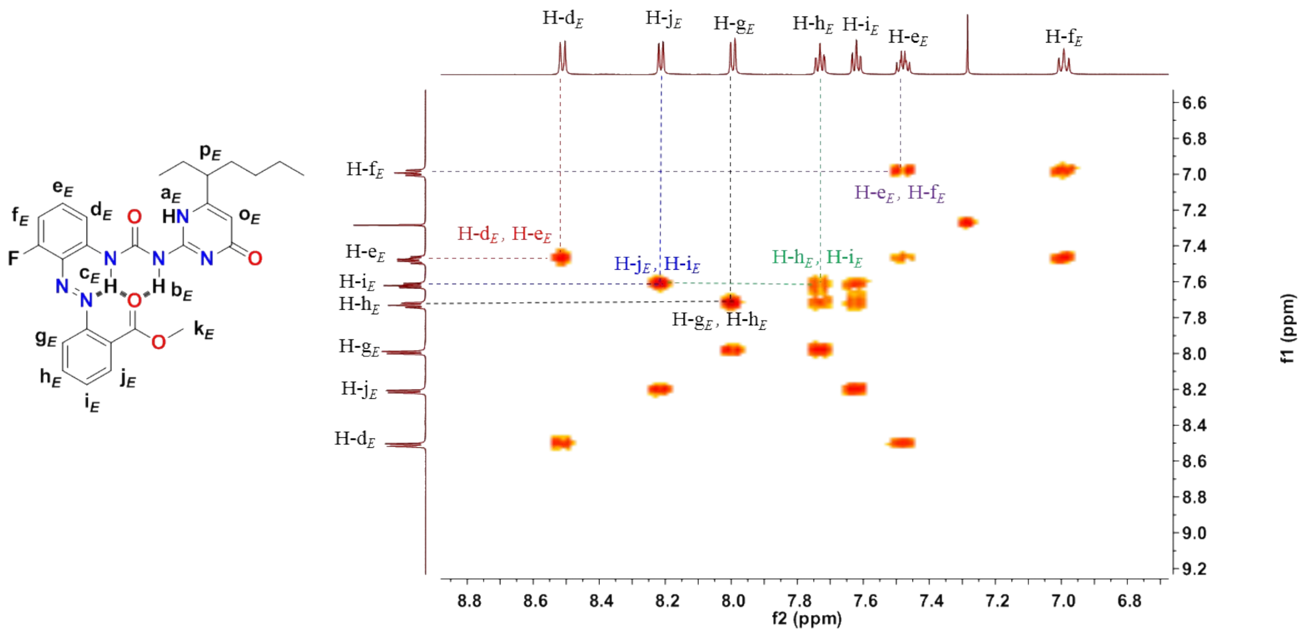


**Fig. S11.** COSY-NMR spectrum (600 MHz, 40 mM, $CDCl_3$, 298K) of the solution of *E*-**Azo-UPy**.

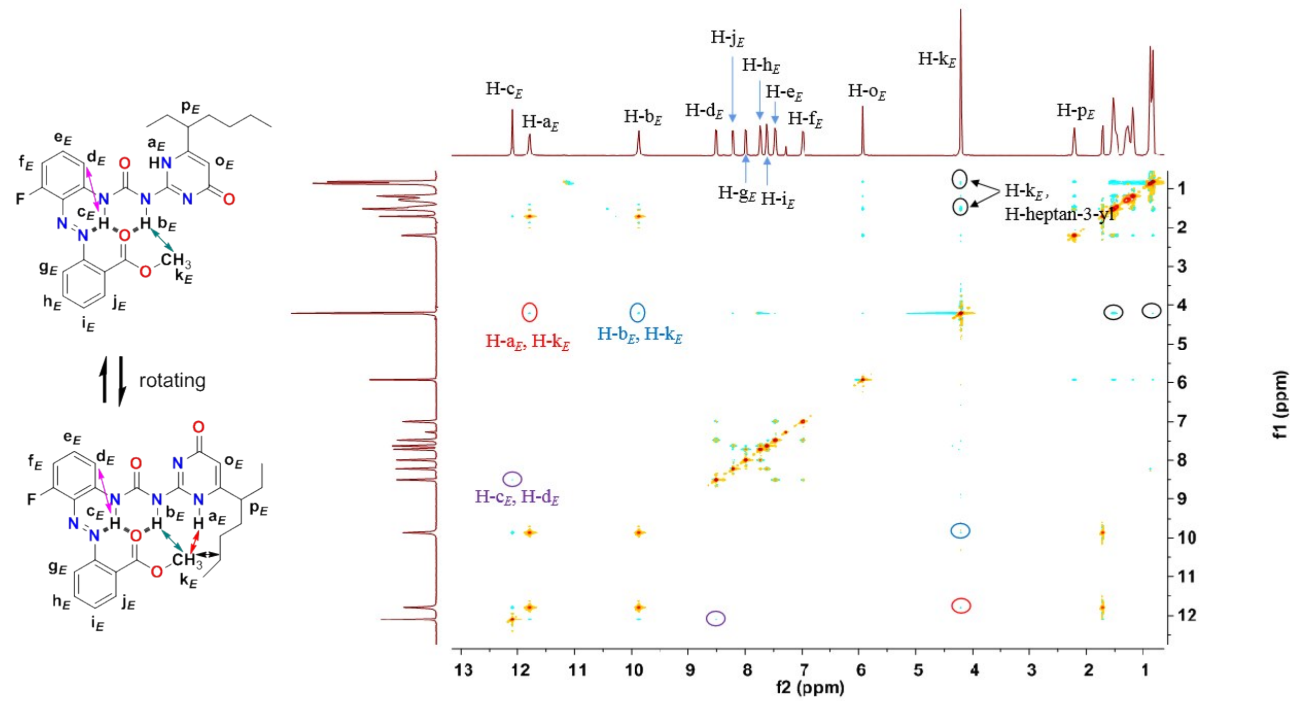


**Fig. S12.** NOESY-NMR spectrum (600 MHz, 40 mM, $CDCl_3$, 298K) of the solution of *E*-**Azo-UPy**.

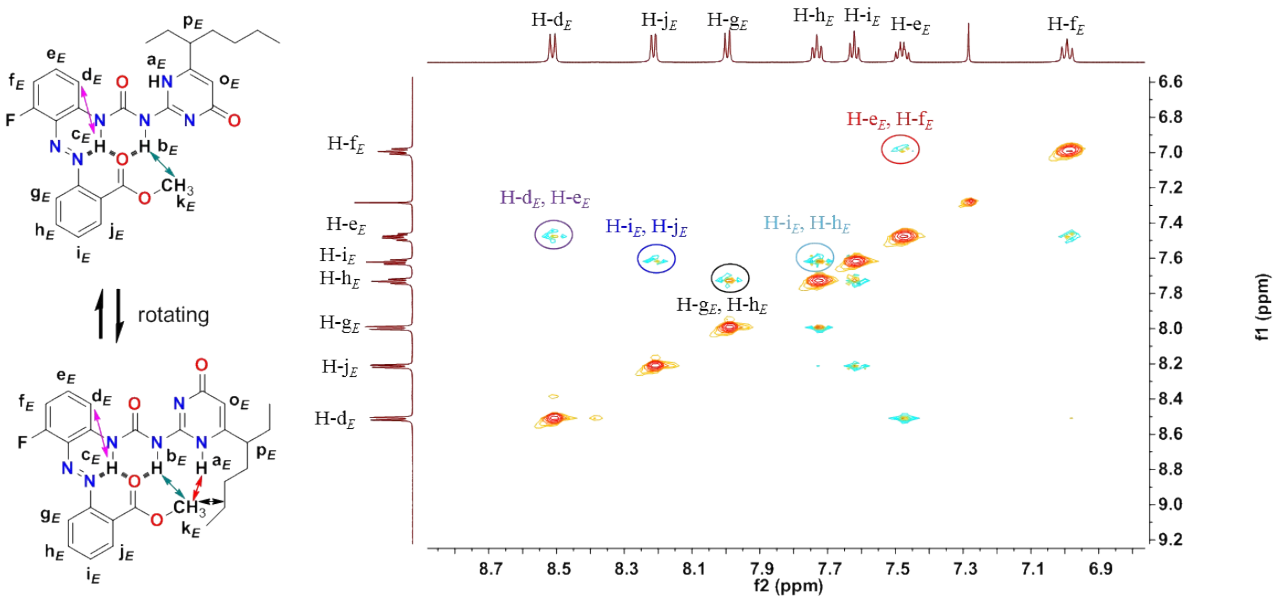


**Fig. S13.** Partial NOESY-NMR spectrum (600 MHz, 40 mM, $CDCl_3$, 298K) of the solution of *E*-**Azo-UPy**.

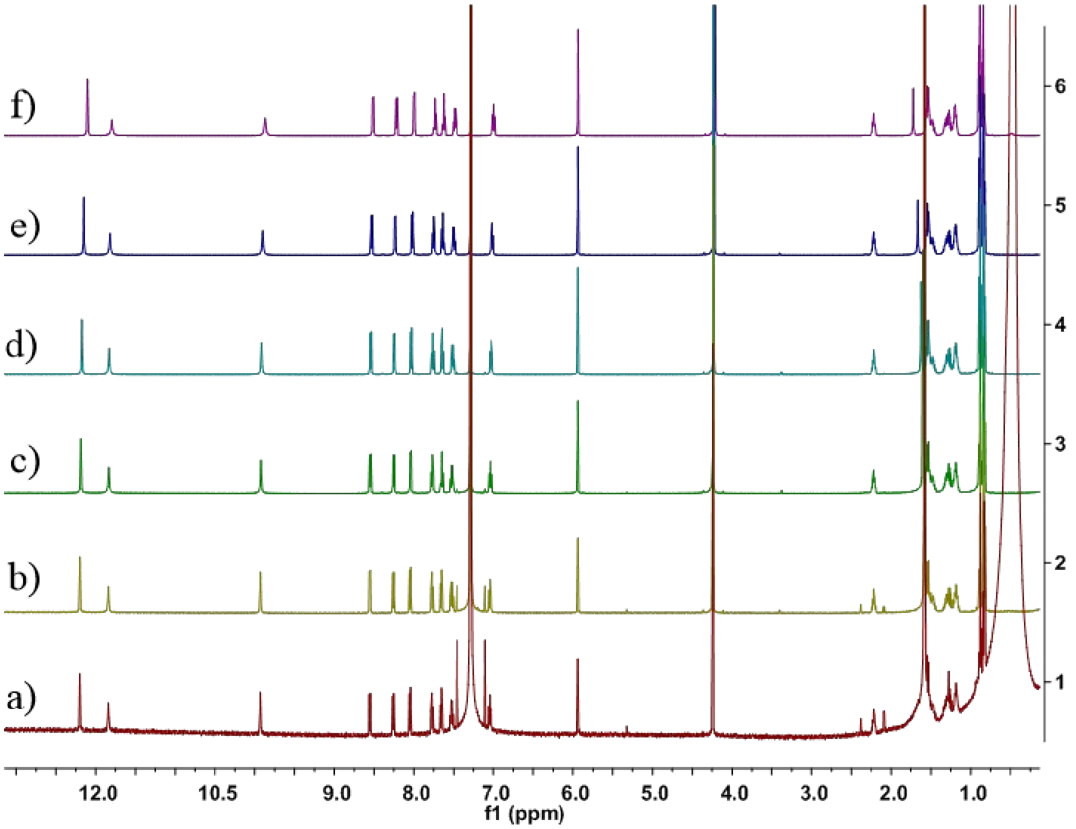


**Fig. S14.** $^{1}$H NMR spectra (600 MHz, $CDCl_3$, 298K) of the solution of *E*-**Azo-UPy** at different concentrations of a) 0.25 mM, b) 1.0 mM, c) 5.0 mM, d) 10 mM, e) 20 mM, and f) 40 mM.

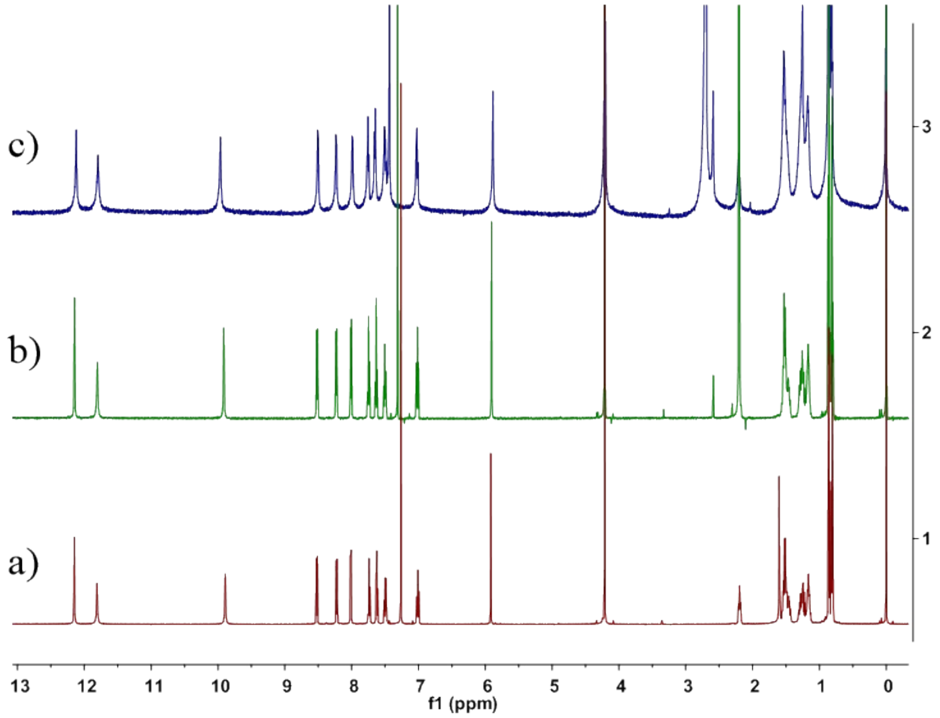


**Fig. S15.** $^{1}$H NMR spectra (600 MHz, 10 mM, 298K) of the solution of *E*-**Azo-UPy** in a) $CDCl_3$, b) $CDCl_3$ / DMSO-$d_6$ (v/v, 39/1), and c) $CDCl_3$ / DMSO-$d_6$ (v/v, 9/1).

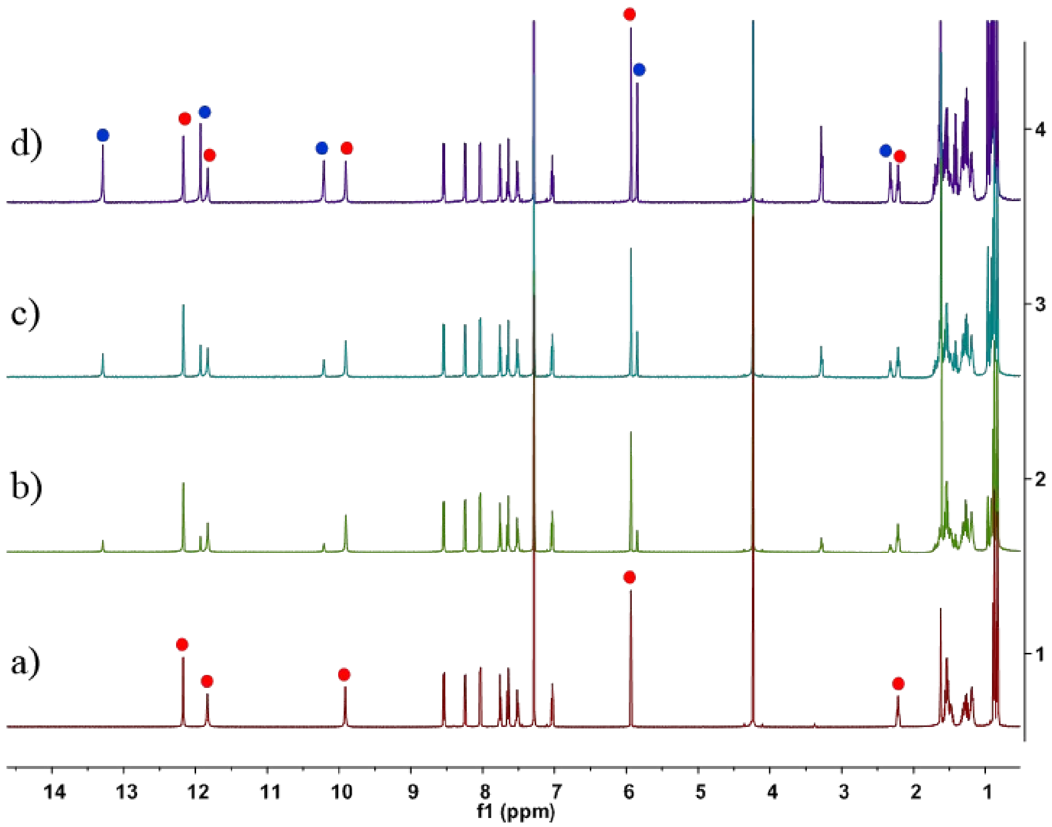


**Fig. S16.** $^{1}$H NMR spectra (600 MHz, 10 mM, 298K) of the solution of *E*-**Azo-UPy** (red dots marked signals) in the presence of a) 0, b) 0.25, c) 0.50, and d) 1.0 equiv. of **UPy-1** (blue dots marked signals).

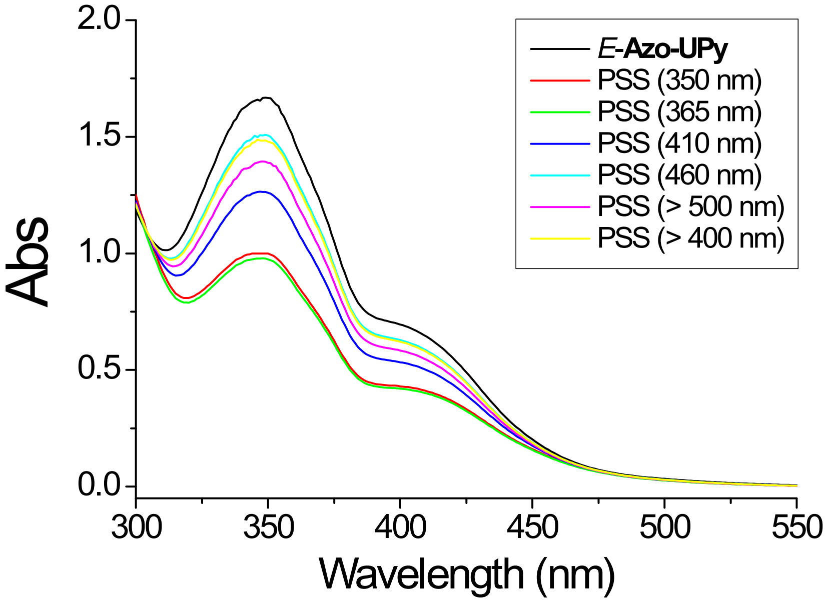


**Fig. S17.** UV-Vis absorption spectra of **Azo-UPy** (0.10 mM) in the pristine state and at the PSS after irradiation with various light sources recorded in $CHCl_3$ at 25 °C.

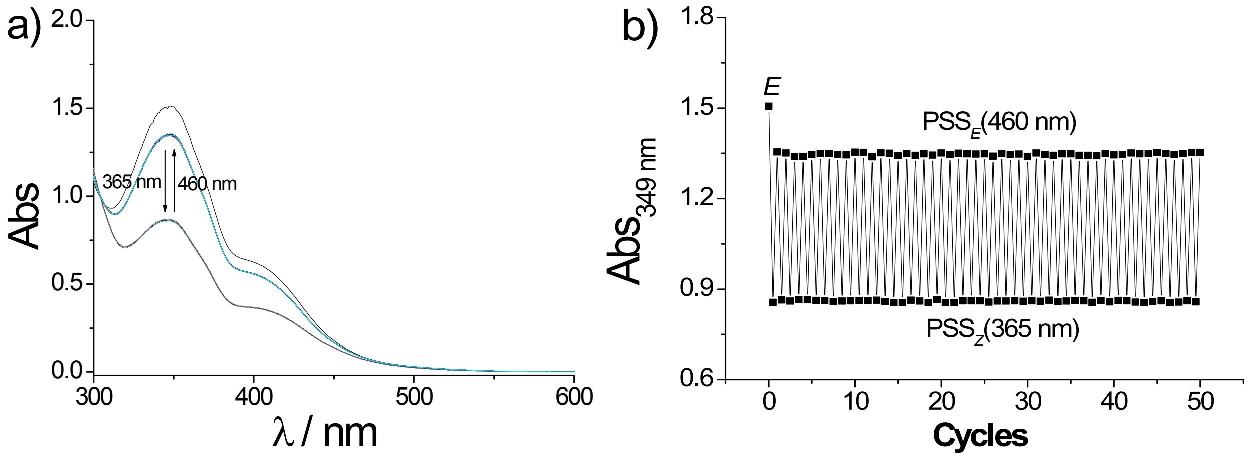


**Fig. S18.** (a) UV-Vis absorption spectra of **Azo-UPy** (0.10 mM), and (b) plot of corresponding absorption λ at 349 nm after UV irradiation (λ = 365 nm, 50 mW / cm$^2$) in $CHCl_3$ for 1.5 min, and UV-irradiated **Azo-UPy** solution after blue light (λ = 460 nm, 20 mW / cm$^2$) irradiation for 2 min before next UV irradiation. The absorption spectra were record at 25 °C.

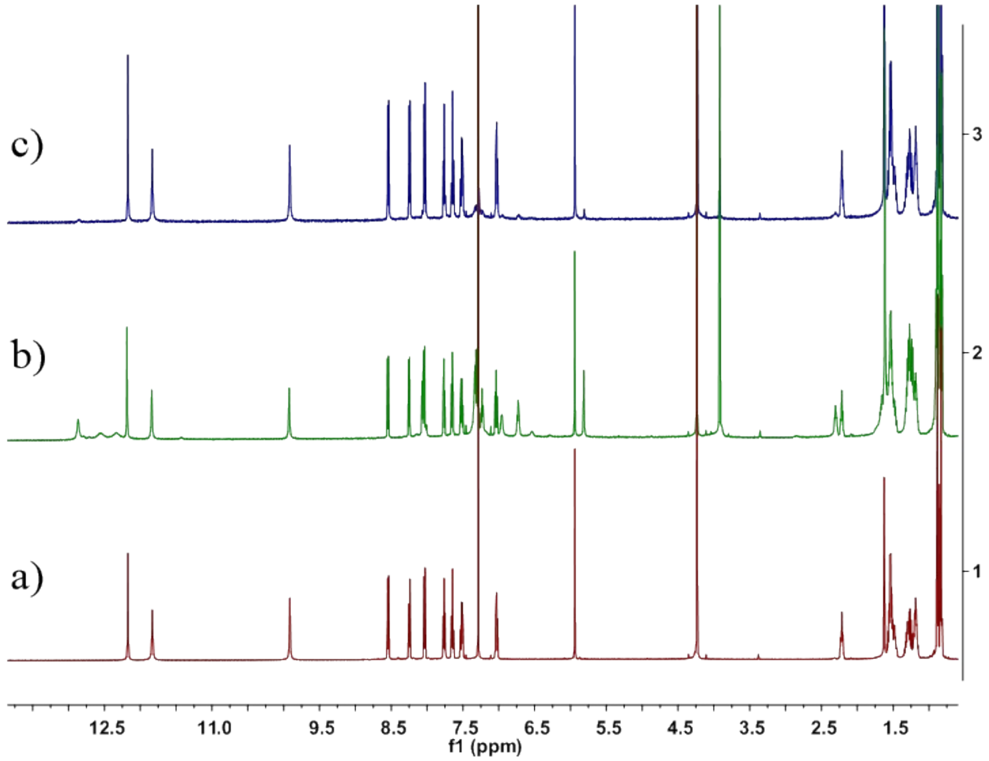


**Fig. S19.** Full $^{1}$H NMR spectra (600 MHz, 10 mM, $CDCl_3$, 298K) of the solution of a) *E*-**Azo-UPy**, b) the $PSS_Z$ (365 nm) mixtures of **Azo-UPy**, and the $PSS_E$ (460 nm) mixtures of **Azo-UPy**.

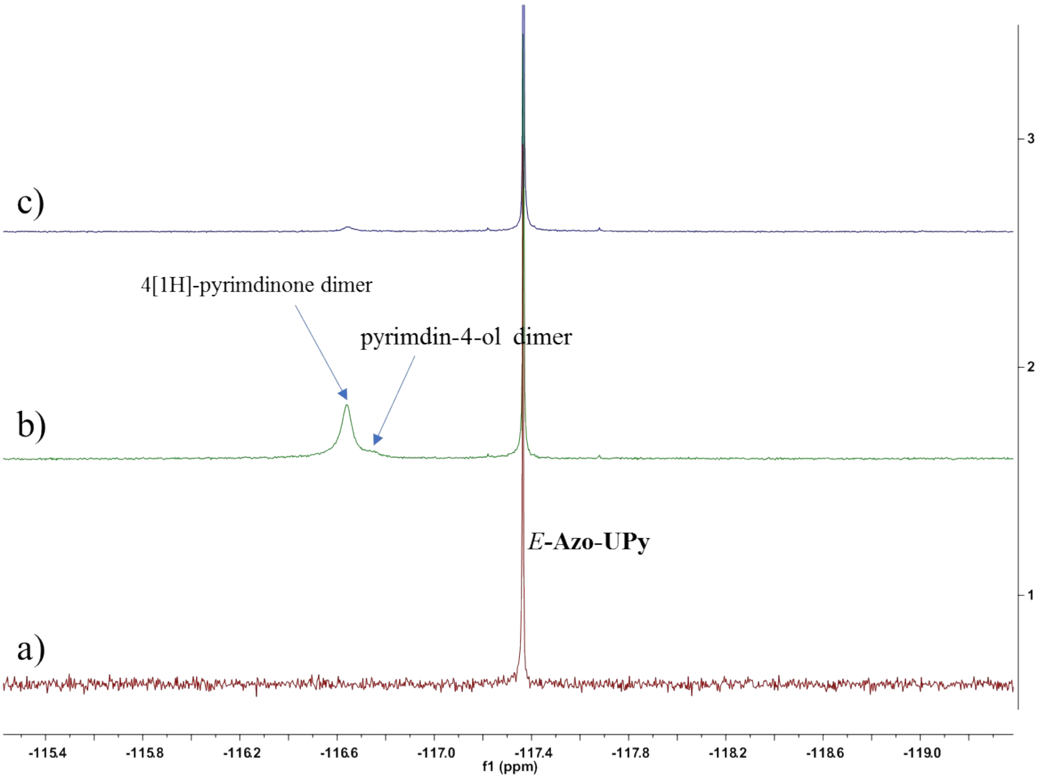


**Fig. S20.** $^{19}$F NMR spectra (564 MHz, 10 mM, $CDCl_3$, 298K) of the solution of a) *E*-**Azo-UPy**, b) the $PSS_Z$ (365 nm) mixtures of **Azo-UPy**, and the $PSS_E$ (460 nm) mixtures of **Azo-UPy**.

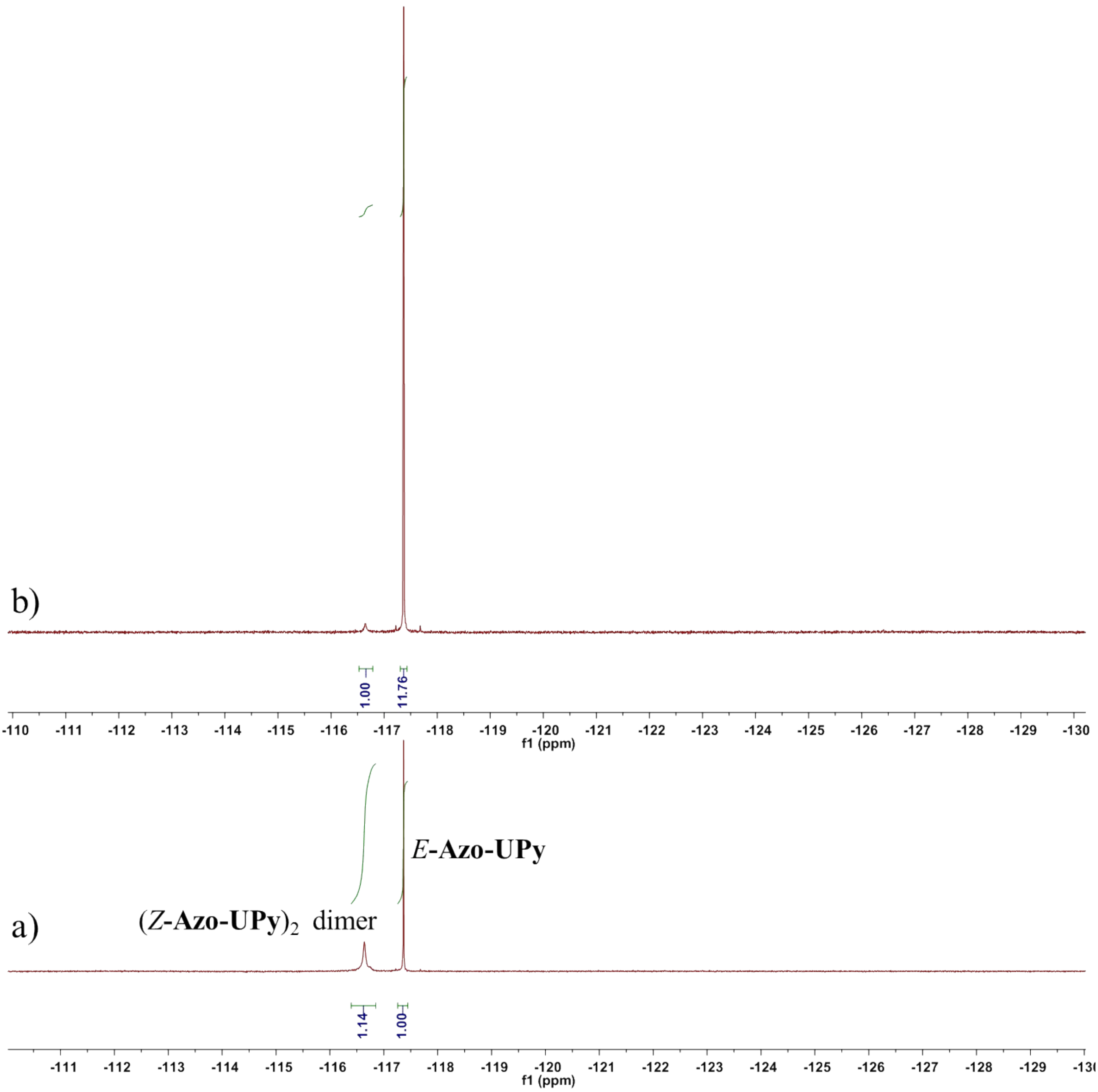


**Fig. S21.** $^{19}$F NMR spectra (564 MHz, $CDCl_3$, 10 mM, 298K) of the solution of a) the $PSS_Z$ (365 nm) mixtures of **Azo-UPy**, and b) the $PSS_E$ (460 nm) mixtures of **Azo-UPy**.

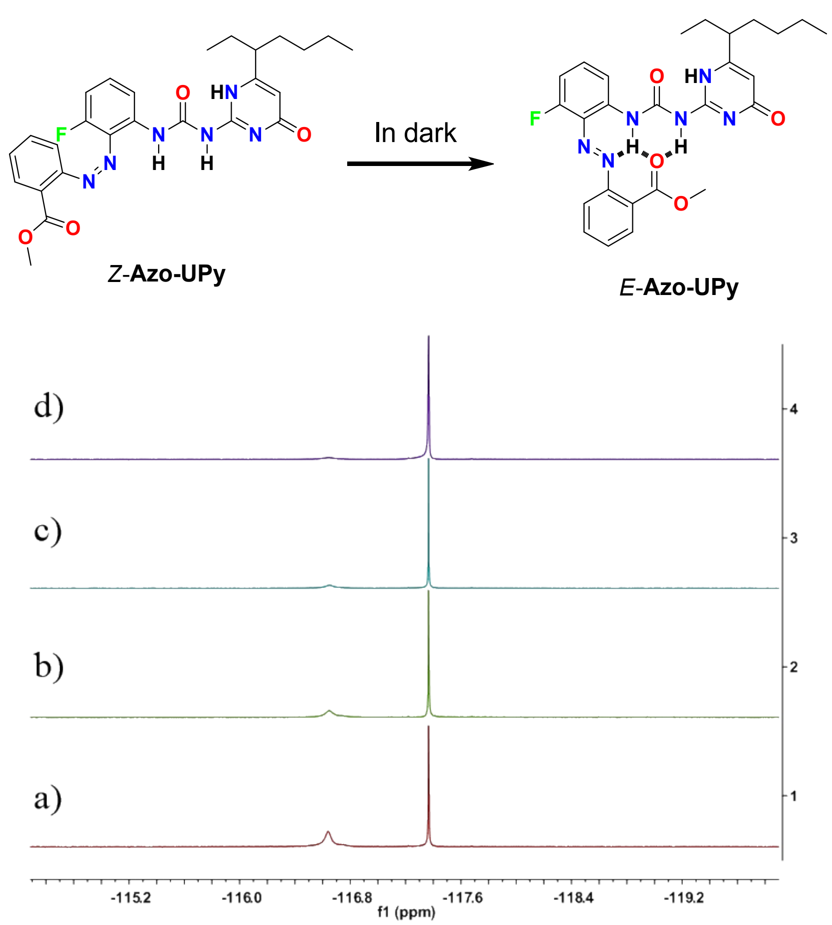


**Fig. S22.** Schematic representation of the *Z*-to-*E* thermal relaxation behavior of **Azo-UPy**, and the $^{19}F$ NMR spectra (564 MHz, 10 mM, $CDCl_3$, 298K) for the solution of the $PSS_Z$ (365 nm) mixtures of **Azo-UPy** a) as prepared, and after the rest of b) 38 h, c) 59 h, d) 131 h at 25 °C in dark.

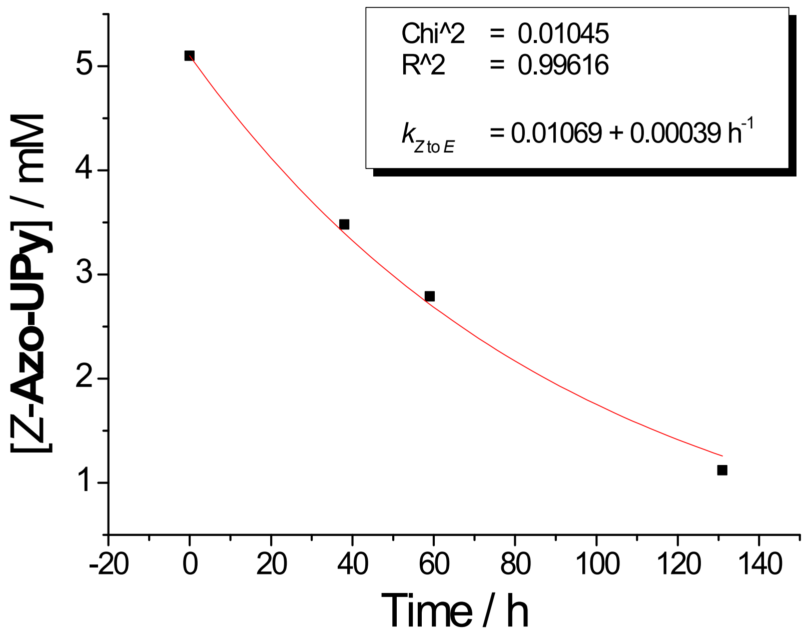


**Fig. S23.** Time dependent concentration change plots and the fitting curve of *Z*-**Azo-UPy** at 25 °C in dark.

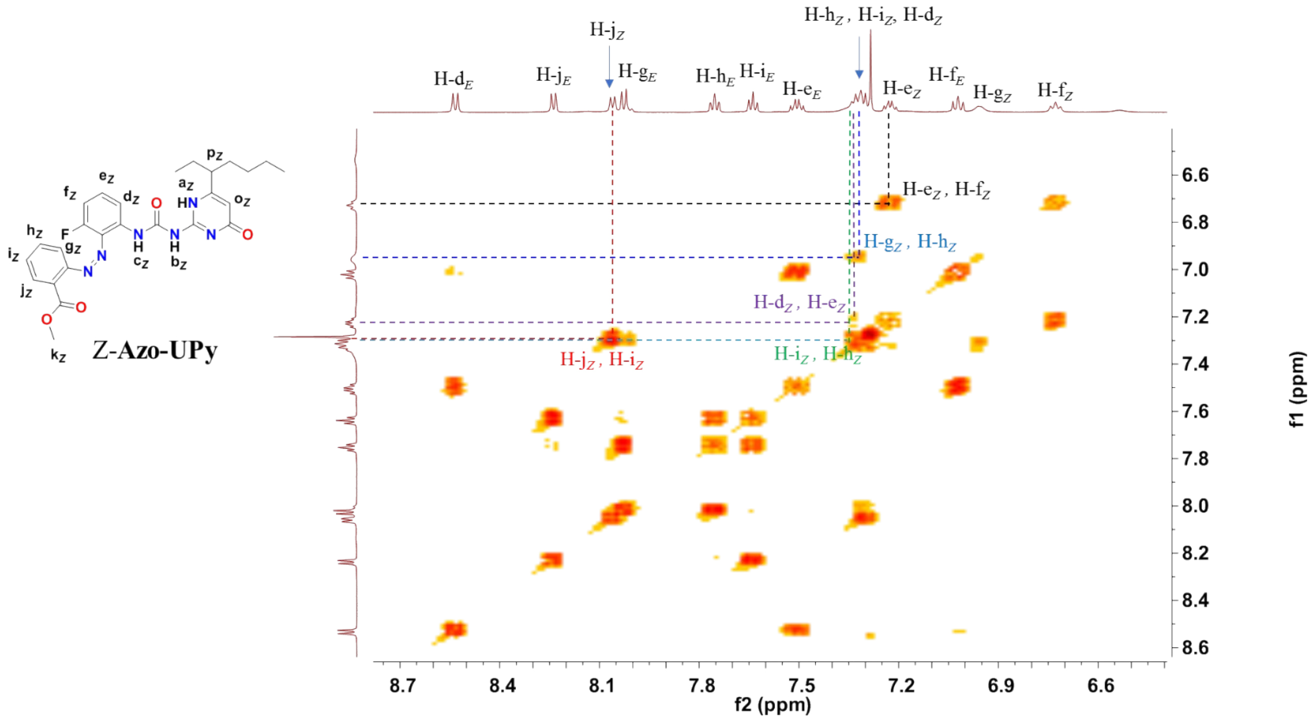


**Fig. S24.** COSY-NMR spectrum (600 MHz, 40 mM, $CDCl_3$, 298K) of the solution of the $PSS_Z$ (365 nm) mixtures of **Azo-UPy**.

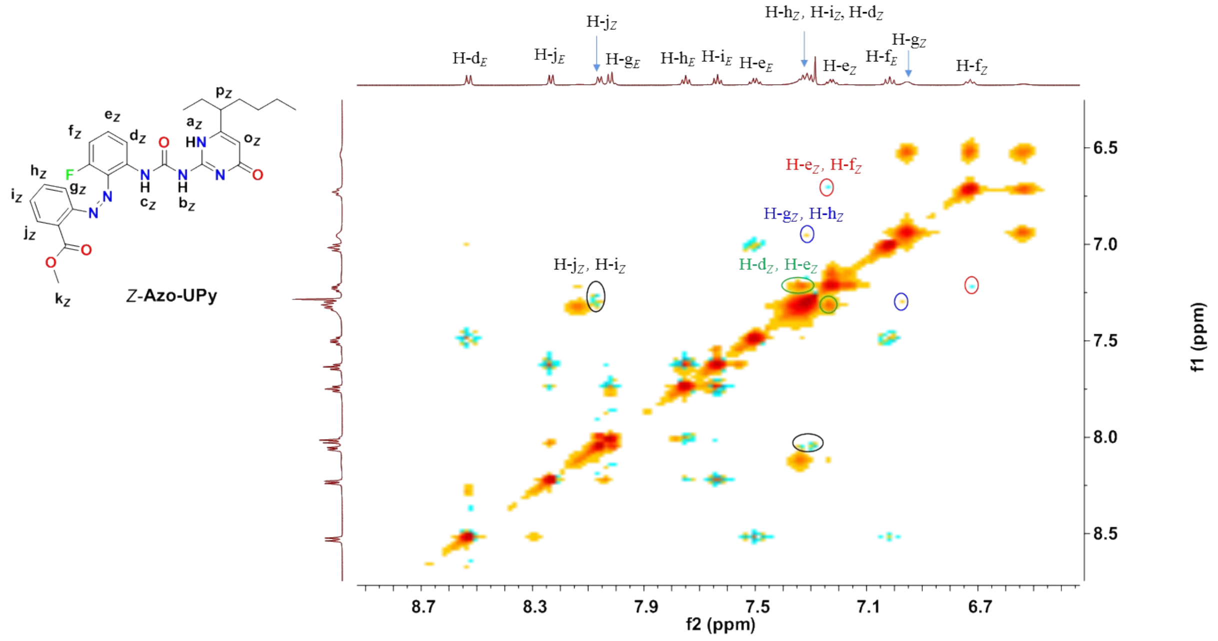


**Fig. S25.** NOSY-NMR spectrum (600 MHz, 40 mM, $CDCl_3$, 298K) of the solution of the $PSS_Z$ (365 nm) mixtures of **Azo-UPy**.

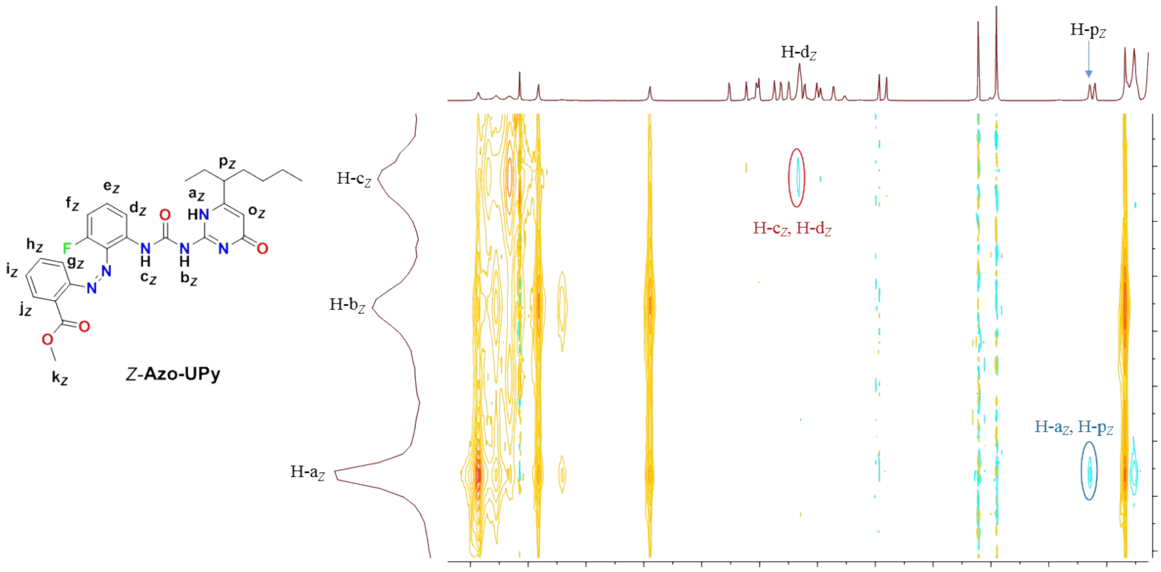


**Fig. S26.** Partial 2D NOSY-NMR spectrum (600 MHz, 40 mM, $CDCl_3$, 298K) of the solution of the $PSS_Z$ (365 nm) mixtures of **Azo-UPy**.

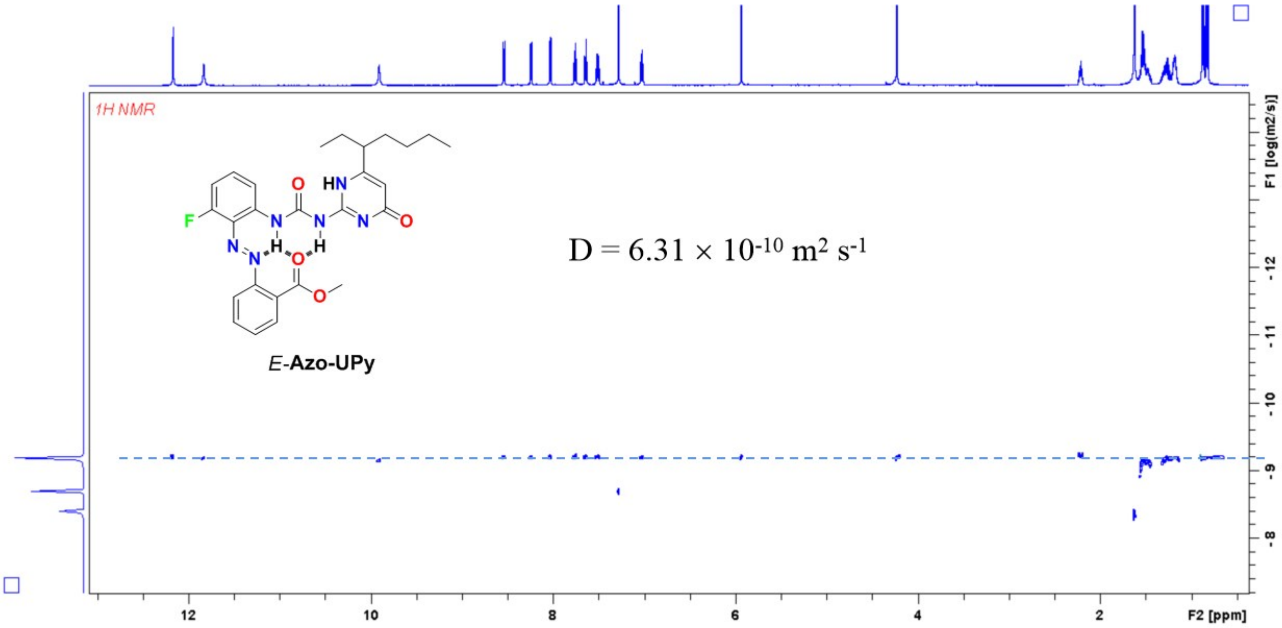


**Fig. S27.** DOSY-NMR spectrum (600 MHz, 40 mM, $CDCl_3$, 298K) of the solution of *E*-**Azo-UPy**.

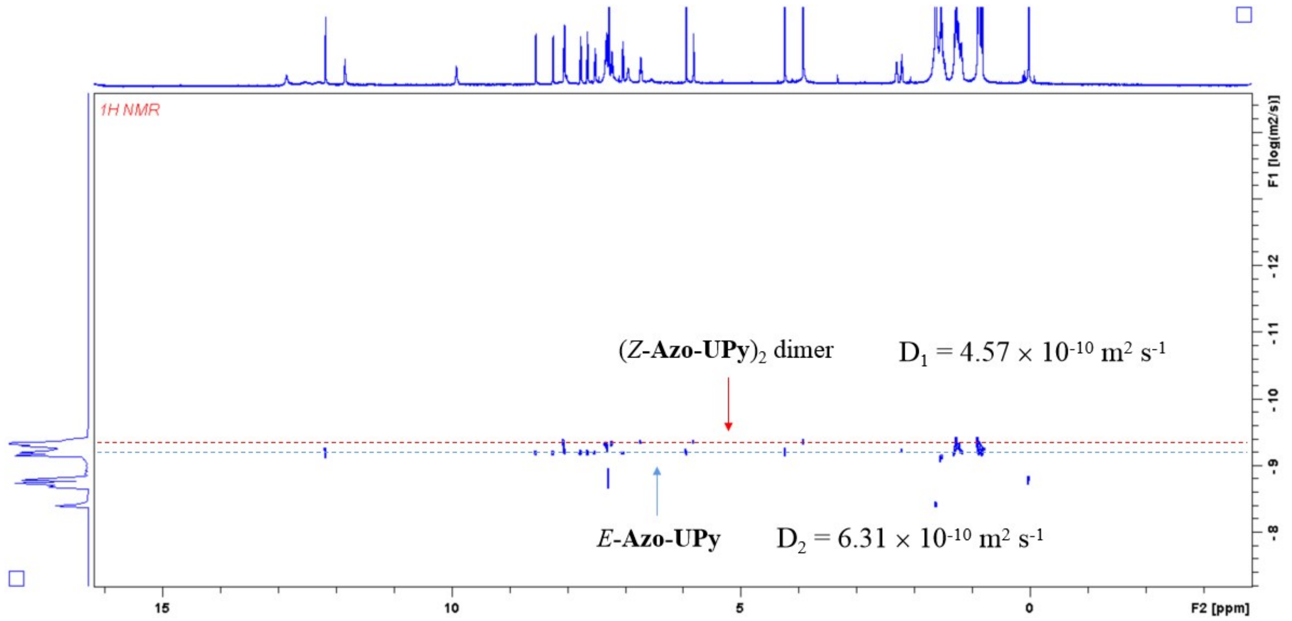


**Fig. S28.** DOSY-NMR spectrum (600 MHz, 40 mM, $CDCl_3$, 298K) of the $PSS_Z$ (365 nm) mixtures of **Azo-UPy**.

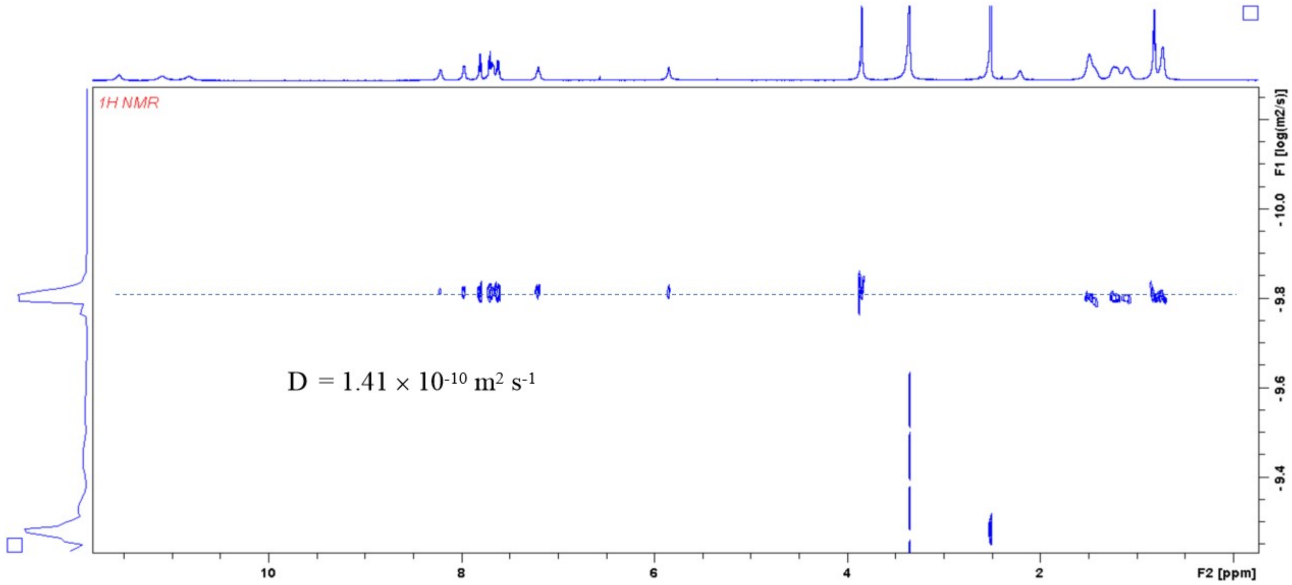


**Fig. S29.** DOSY-NMR spectrum (600 MHz, 40 mM, DMSO-$d_6$, 298K) of the solution of the *E*-**Azo-UPy**.

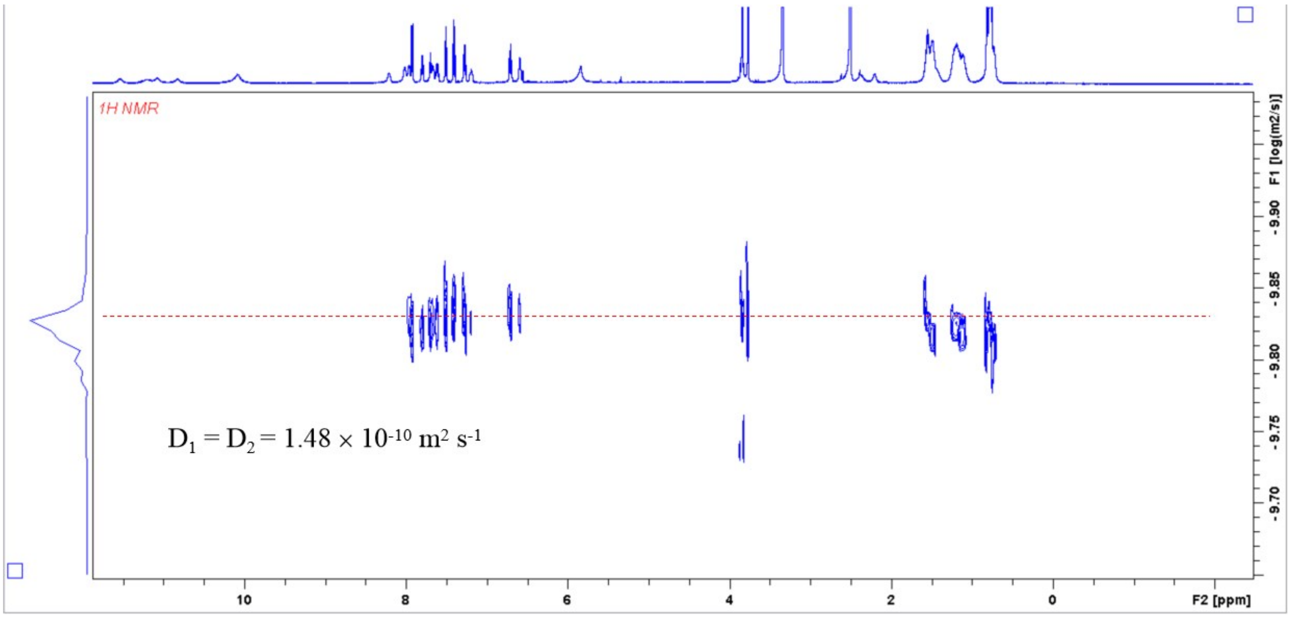


**Fig. S30.** DOSY-NMR spectrum (600 MHz, 40 mM, DMSO-$d_6$, 298K) of the solution of the $PSS_Z$ (365 nm) mixtures of **Azo-UPy**.

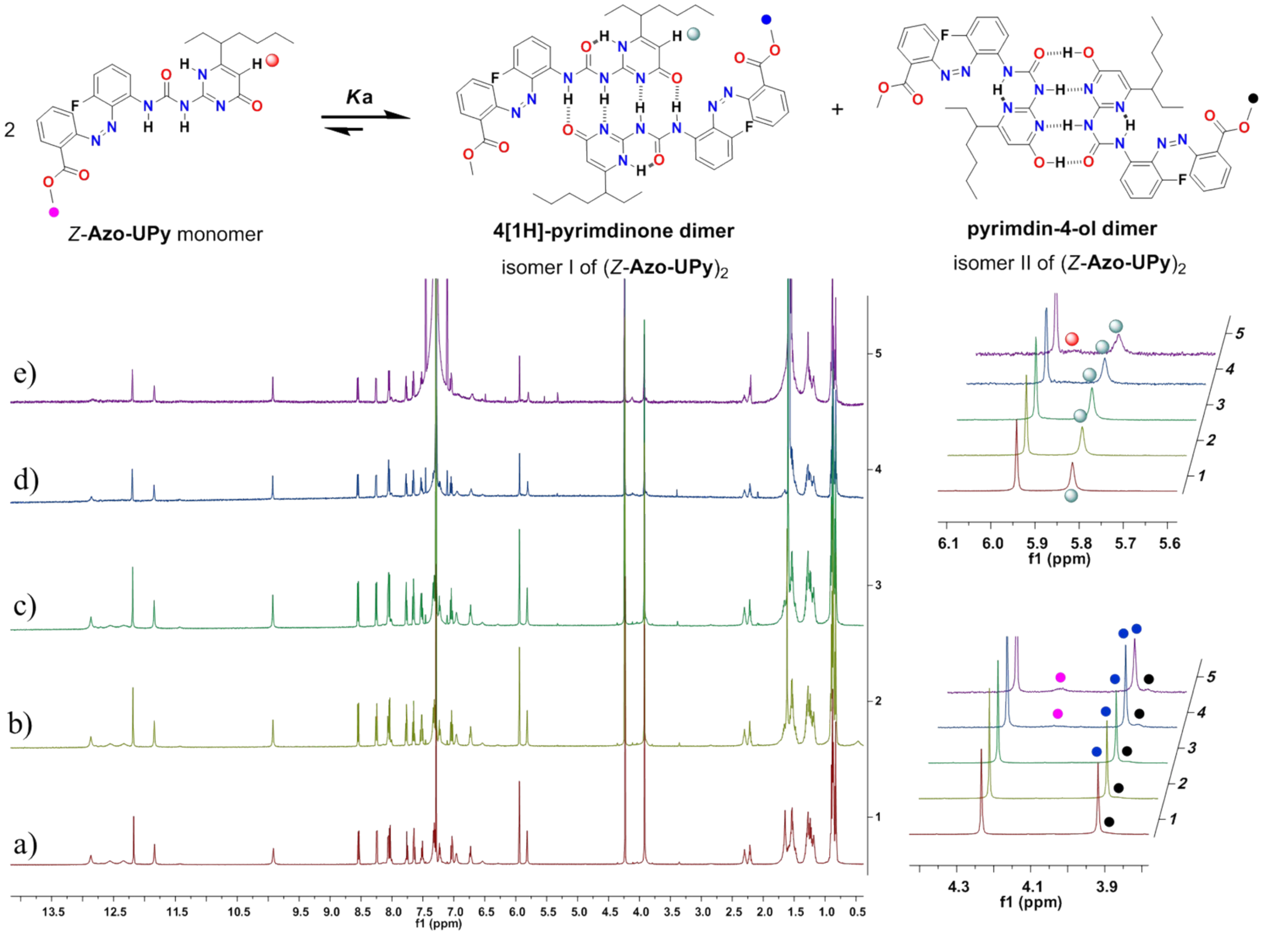


**Fig. S31.** $^1H$ NMR spectra (600 MHz, $CDCl_3$, 298K) of the solution of the $PSS_Z$ (365 nm) mixtures of **Azo-UPy** at different concentrations of a) 20 mM, b) 10 mM, c) 5.0 mM, d) 1.0 mM, and e) 0.25 mM.

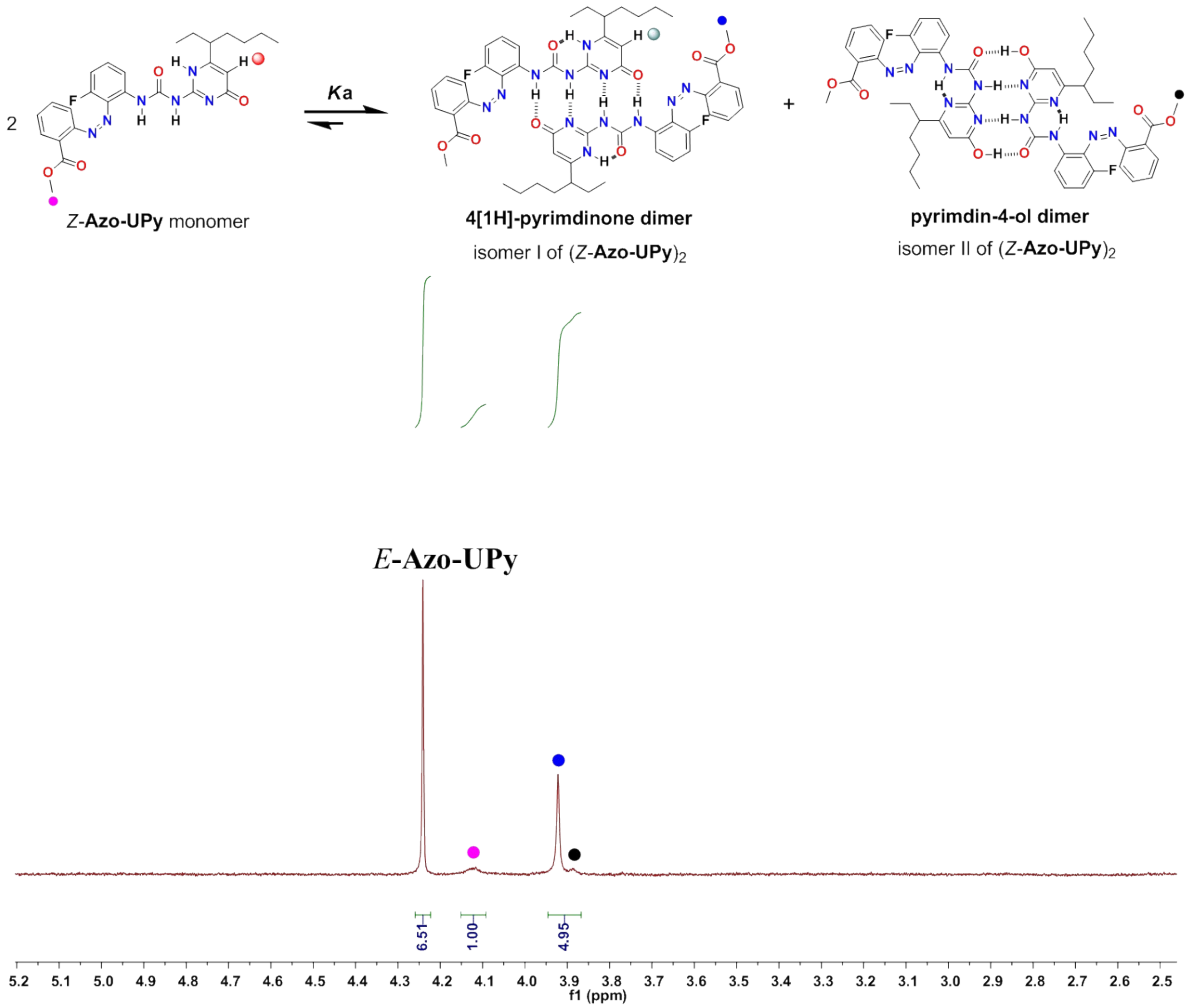


**Fig. S32.** Partial $^{1}$H NMR spectra (600 MHz, 0.25 mM, $CDCl_3$, 298K) of the solution of the $PSS_Z$ (365 nm) mixtures of **Azo-UPy**.

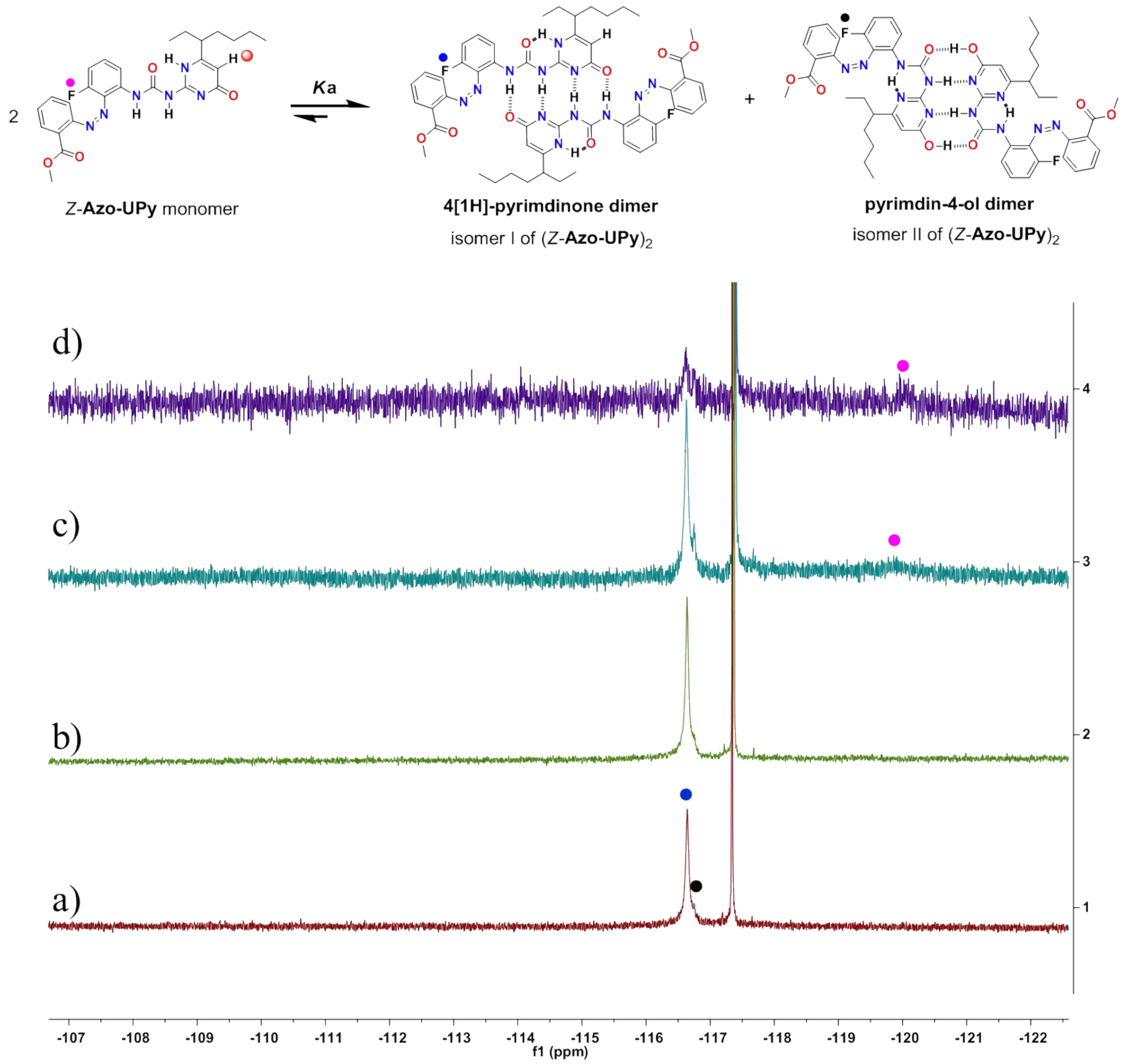


**Fig. S33.** $^{19}$F NMR spectra (564 MHz, $CDCl_3$, 298K) of the solution of the $PSS_Z$ (365 nm) mixtures of **Azo-UPy** at different concentrations of a) 20 mM, b) 10 mM, c) 1.0 mM, and d) 0.25 mM.

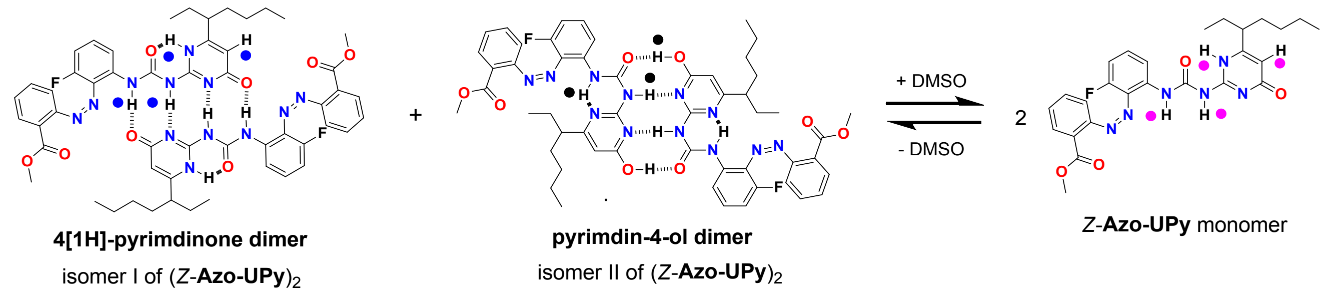


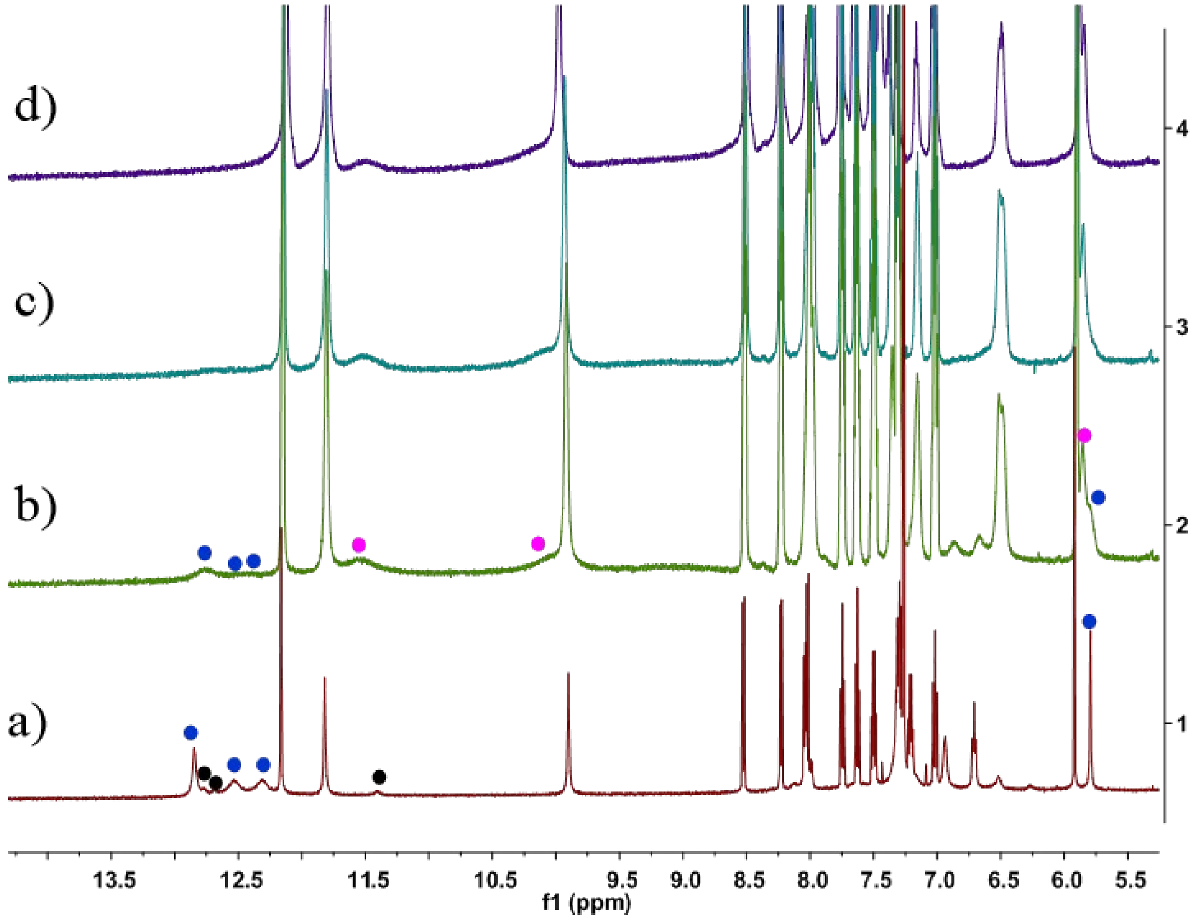


**Fig. S34.** Schematic representation of the disassociation behavior of (*Z*-**Azo-UPy**)$_2$ dimer upon the addition of strong H-bond competitive solvent of DMSO, and the $^1$H NMR spectra (600 MHz, 10 mM, 298K) of the solution of the PSS$_Z$ (365 nm) mixtures of **Azo-UPy** in a) $CDCl_3$, b) $CDCl_3$ / DMSO-$d_6$ (v/v, 39/1), c) $CDCl_3$ / DMSO-$d_6$ (v/v, 19/1), and d) $CDCl_3$ / DMSO-$d_6$ (v/v, 9/1).

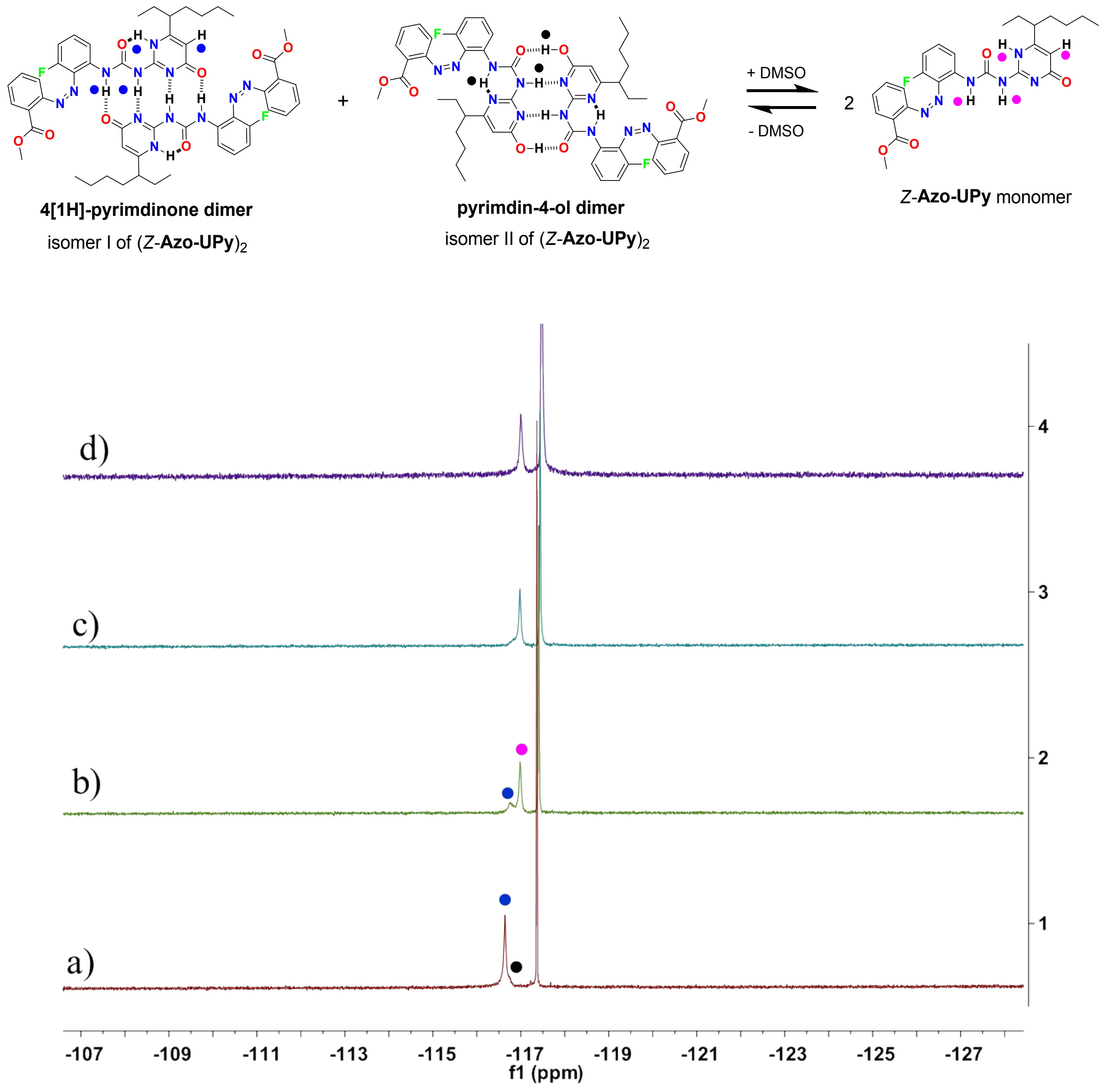


**Fig. S35.** Schematic representation of the disassociation behavior of ($Z$-**Azo-UPy**)$_2$ dimer upon the addition of strong H-bond competitive solvent of DMSO, and the $^{19}$F NMR spectra (564 MHz, 10 mM, 298K) of the solution of the PSS$_Z$ (365 nm) mixtures of **Azo-UPy** in a) $CDCl_3$, b) $CDCl_3$ / DMSO-$d_6$ (v/v, 39/1), c) $CDCl_3$ / DMSO-$d_6$ (v/v, 19/1), and d) $CDCl_3$ / DMSO-$d_6$ (v/v, 9/1).

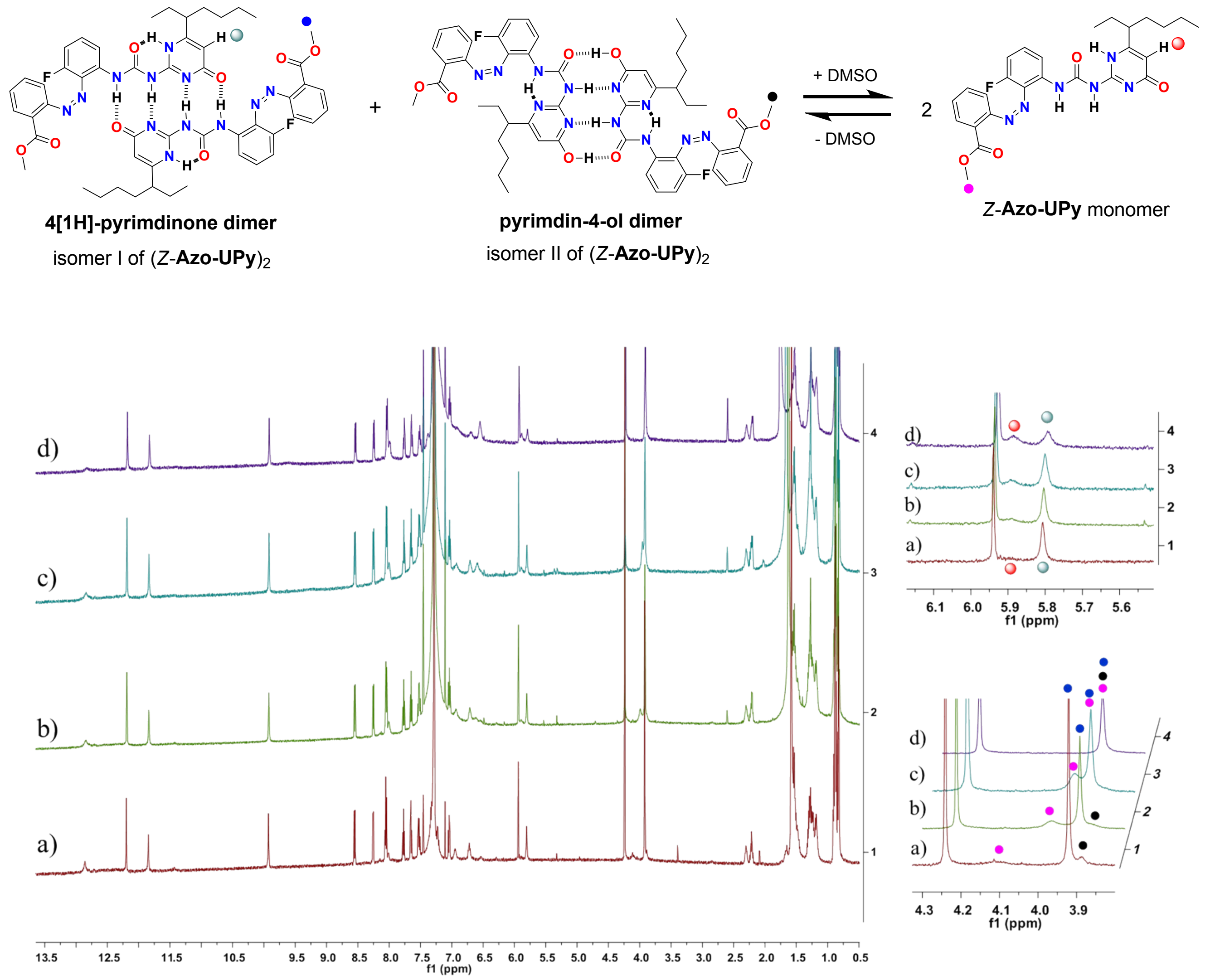


**Fig. S36.** Schematic representation of the disassociation behavior of (*Z*-**Azo-UPy**)$_2$ dimer upon the addition of strong H-bond competitive solvent of DMSO, and the $^1$H NMR spectra (600 MHz, 1.0 mM, 298K) of the solution of the PSS$_Z$ (365 nm) mixtures of **Azo-UPy** in a) $CDCl_3$, b) $CDCl_3$ / DMSO-$d_6$ (v/v, 999/1), c) $CDCl_3$ / DMSO-$d_6$ (v/v, 499/1), and d) $CDCl_3$ / DMSO-$d_6$ (v/v, 199/1).

## Section 5: Photoswitchable quadruple H-bonded hetero-dimerization of Azo-UPy.

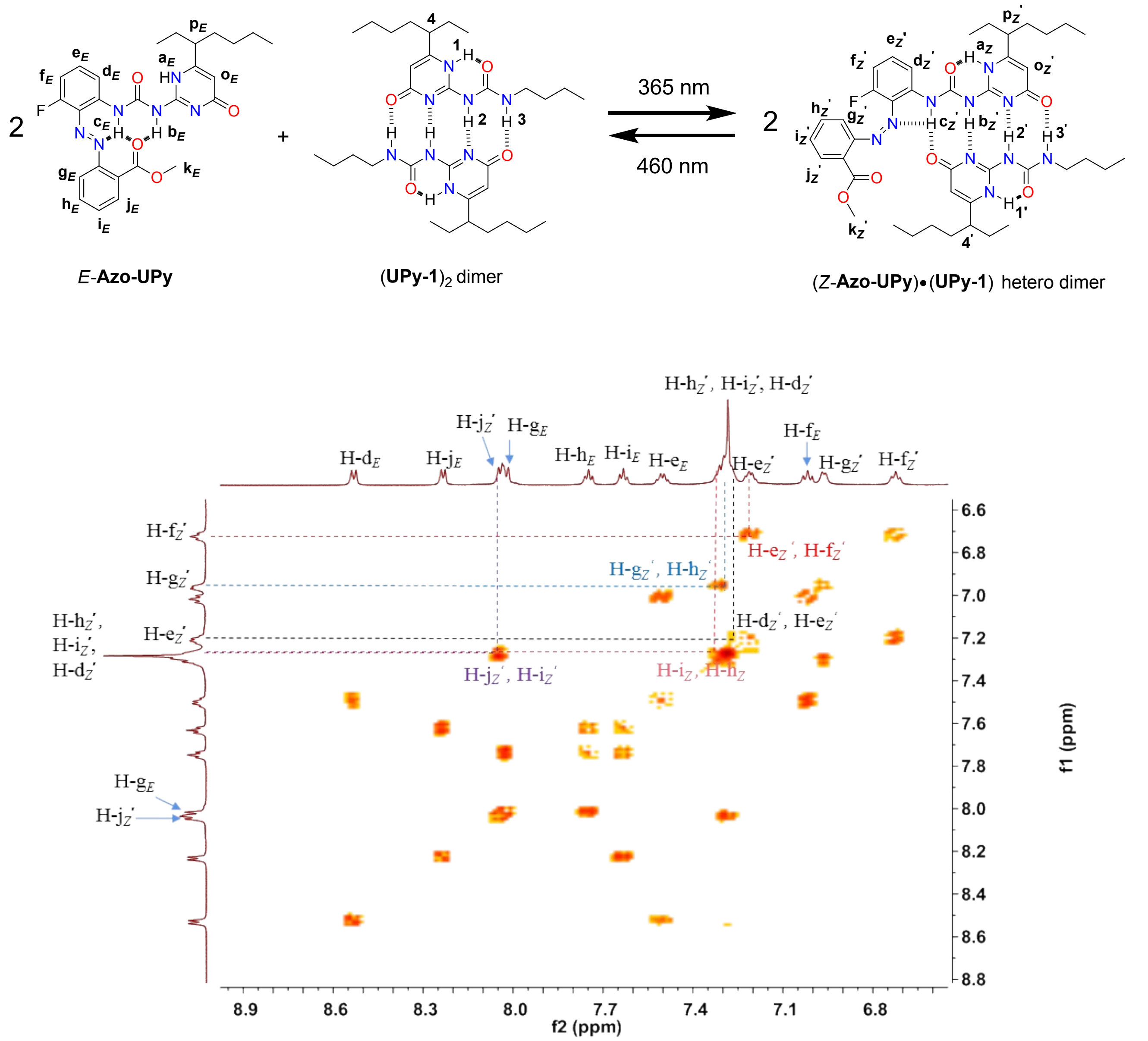


**Fig. S37.** Schematic representation of the photocontrolled formation of quadruple H-bonded hetero dimer of (*Z*-**Azo-UPy**)•(**UPy-1**), and the 2D COSY-NMR spectrum (600 MHz, $CDCl_3$, 298K) of the solution of **UPy-1** (80 mM) and the PSS$_Z$ (365 nm) mixtures of **Azo-UPy** (20 mM).

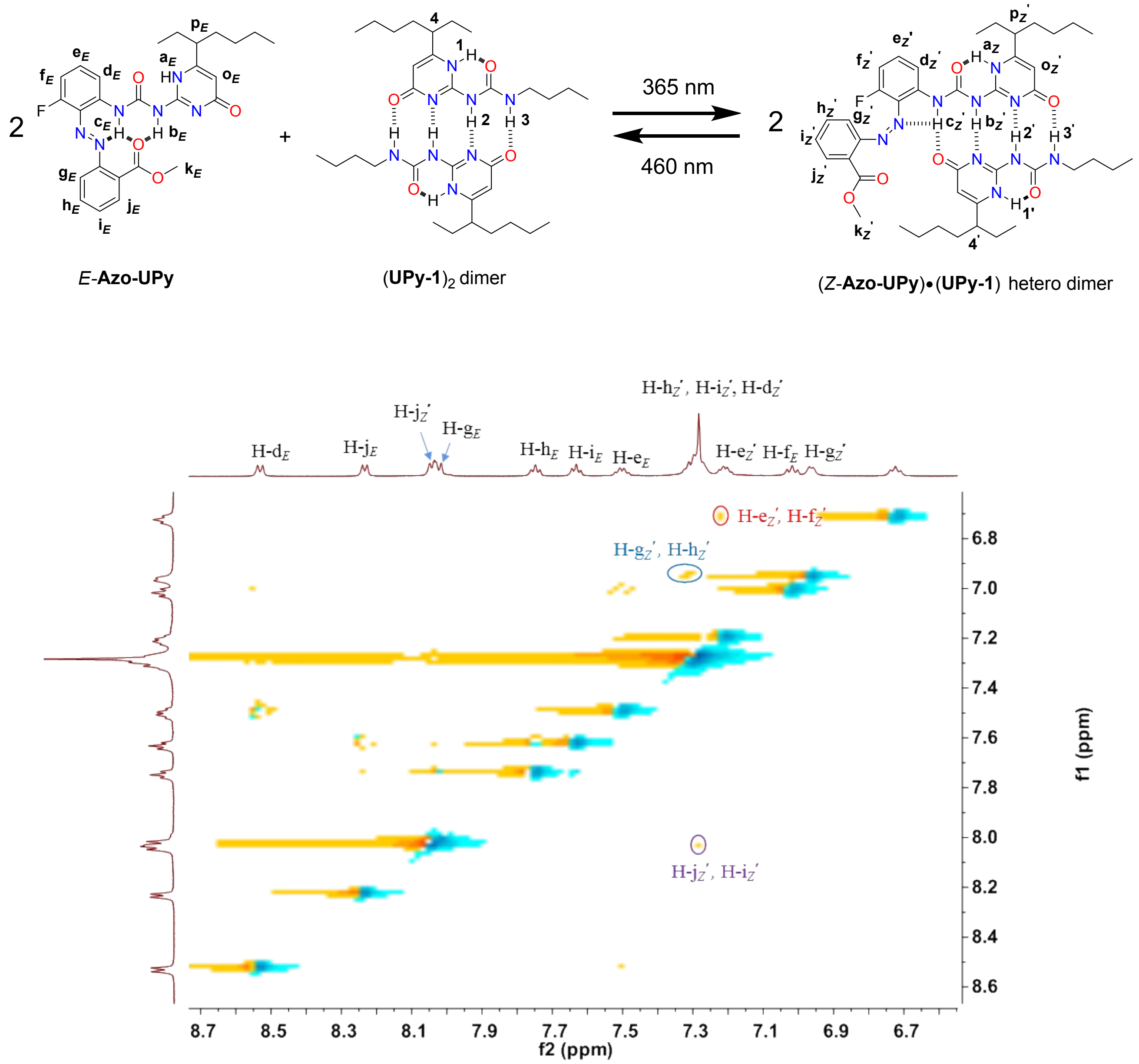


**Fig. S38.** Schematic representation of the photocontrolled formation of quadruple H-bonded hetero dimer of (*Z*-**Azo-UPy**)•(**UPy-1**), and the 2D NOESY-NMR spectrum (600 MHz, $CDCl_3$, 298K) of the solution of **UPy-1** (80 mM) and the $PSS_Z$ (365 nm) mixtures of **Azo-UPy** (20 mM).

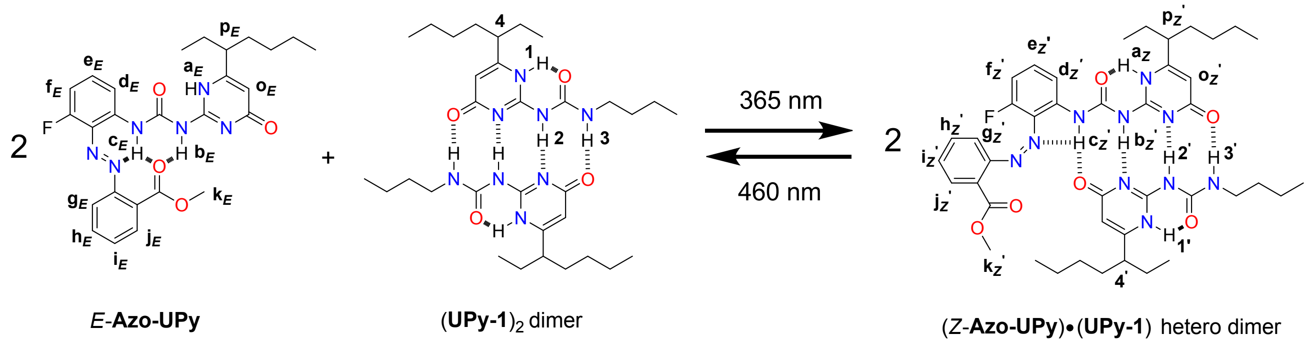


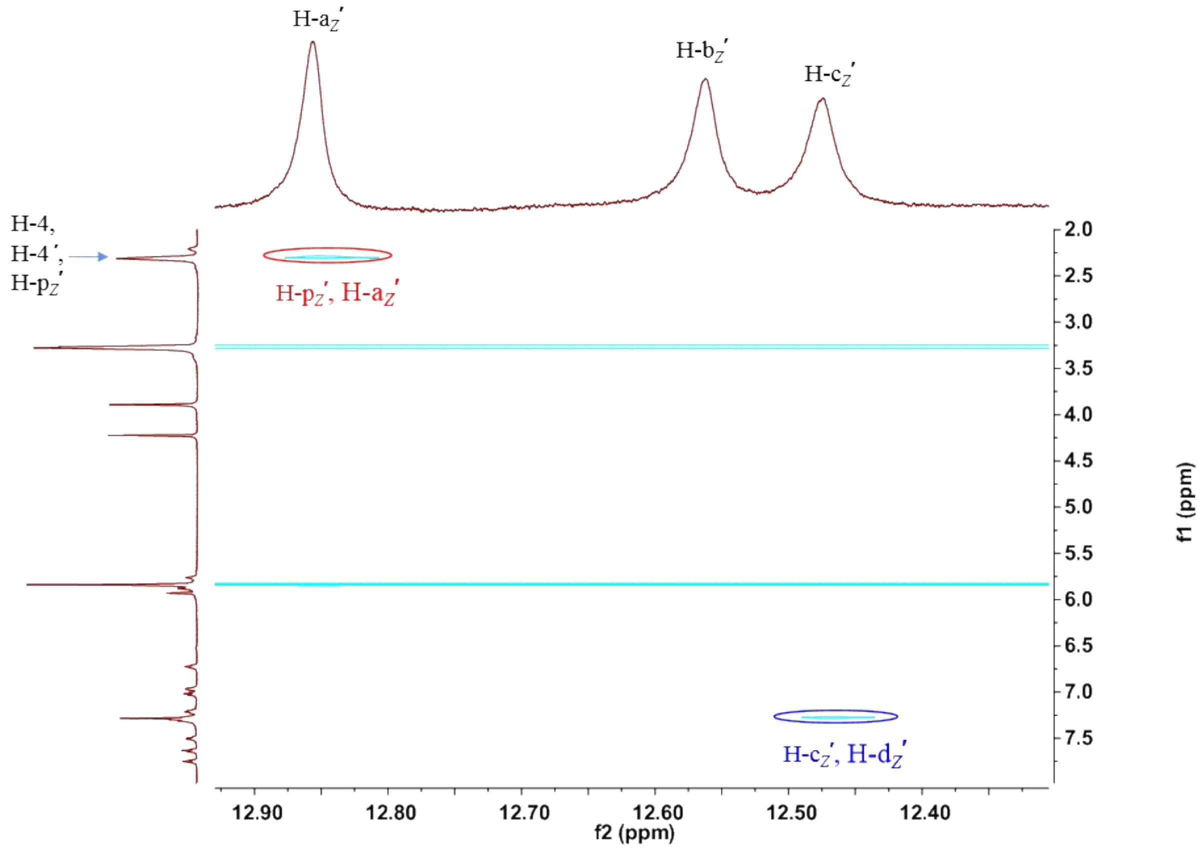


**Fig. S39.** Schematic representation of the photocontrolled formation of quadruple H-bonded hetero dimer of (*Z*-**Azo-UPy**)•(**UPy-1**), and the partial 2D NOESY-NMR spectrum (600 MHz, $CDCl_3$, 298K) of the solution of **UPy-1** (80 mM) and the $PSS_Z$ (365 nm) mixtures of **Azo-UPy** (20 mM).

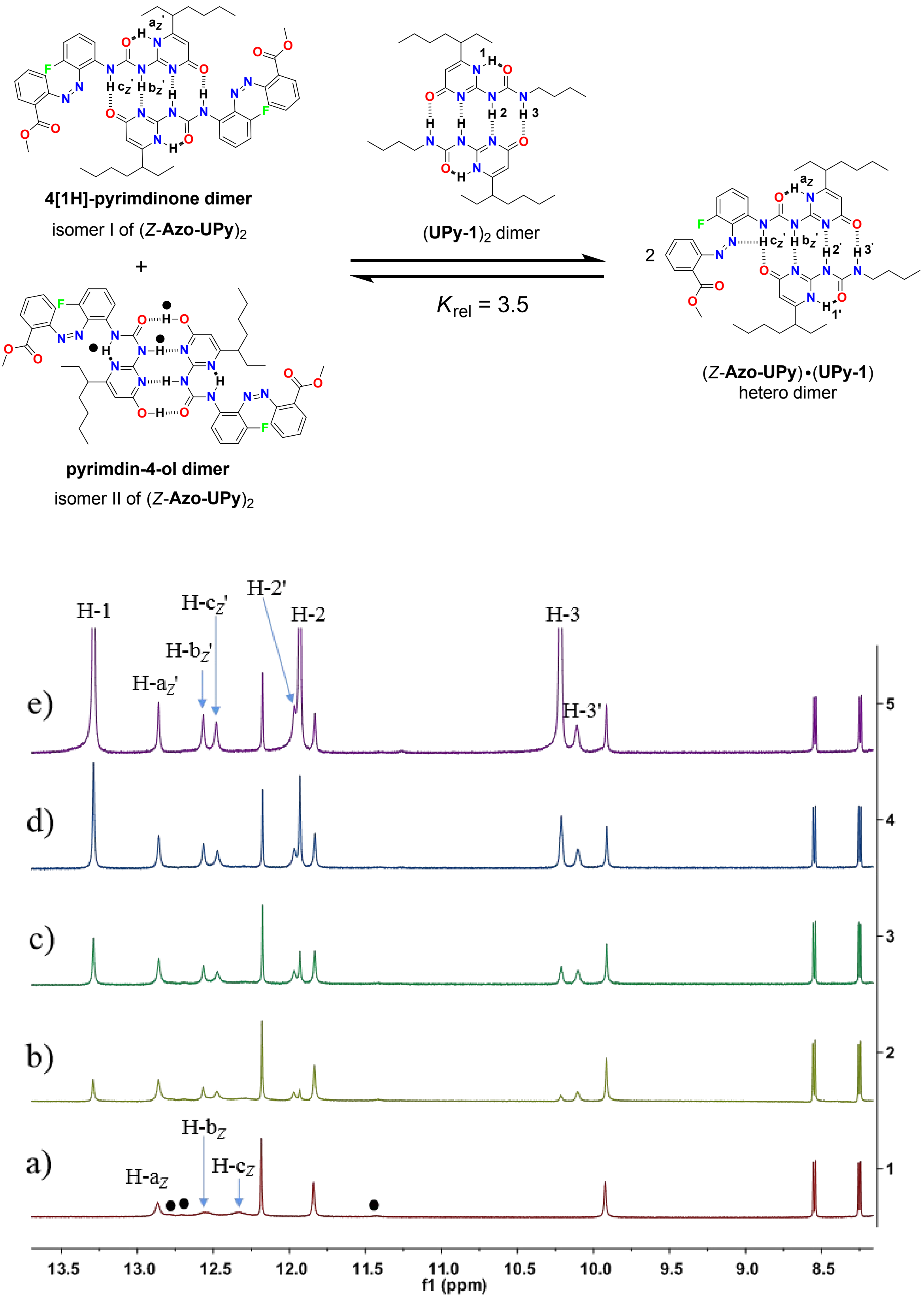


**Fig. S40.** Schematic illustration of the hetero dimerization of *Z*-**Azo-UPy** and **UPy-1**, and the $^{1}$H NMR spectra (600 MHz, $CDCl_3$, 298K) for the solution of the $PSS_Z$ (365 nm) mixtures of **Azo-UPy** (10 mM) in the presence of a) 0, b) 0.25, c) 0.50, d) 1.0, and e) 4.0 equivalent of **UPy-1**.

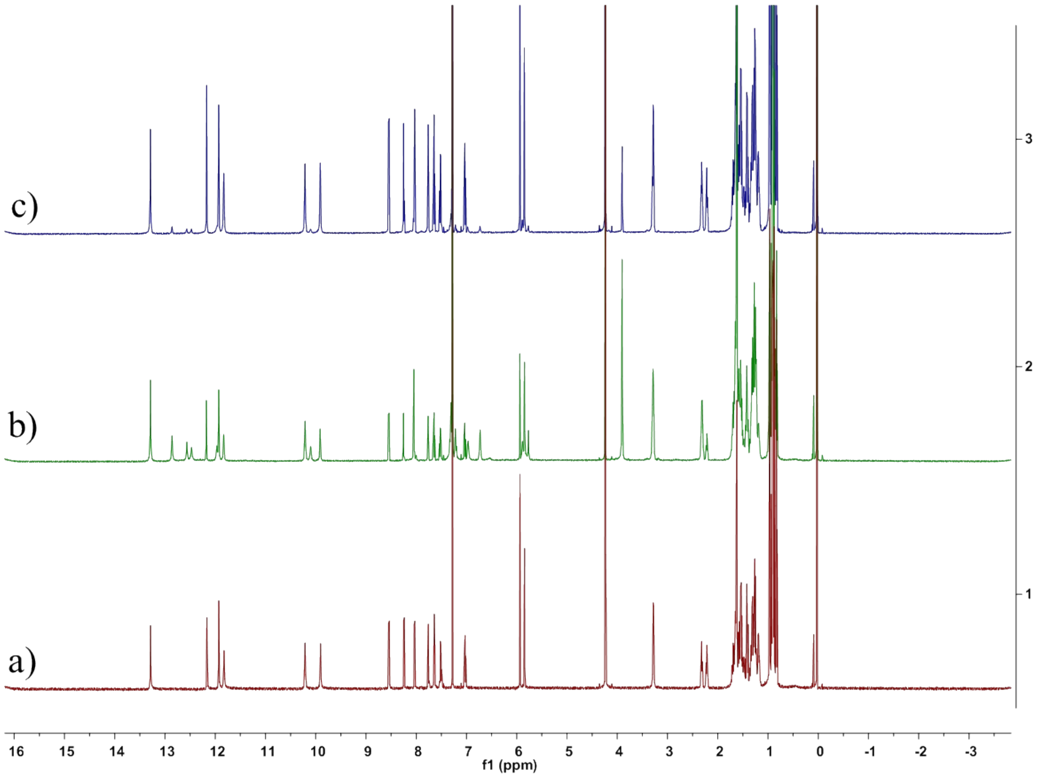


**Fig. S41.** Full $^{1}$H NMR spectra (600 MHz, $CDCl_3$, 298K) of a) the solution of *E*-**Azo-UPy** (10 mM) and **UPy-1** (10 mM) before irradiation, b) the $PSS_Z$ (365 nm) mixtures of **Azo-UPy** and **UPy-1**, and c) the $PSS_E$ (460 nm) mixtures of **Azo-UPy** and **UPy-1**.

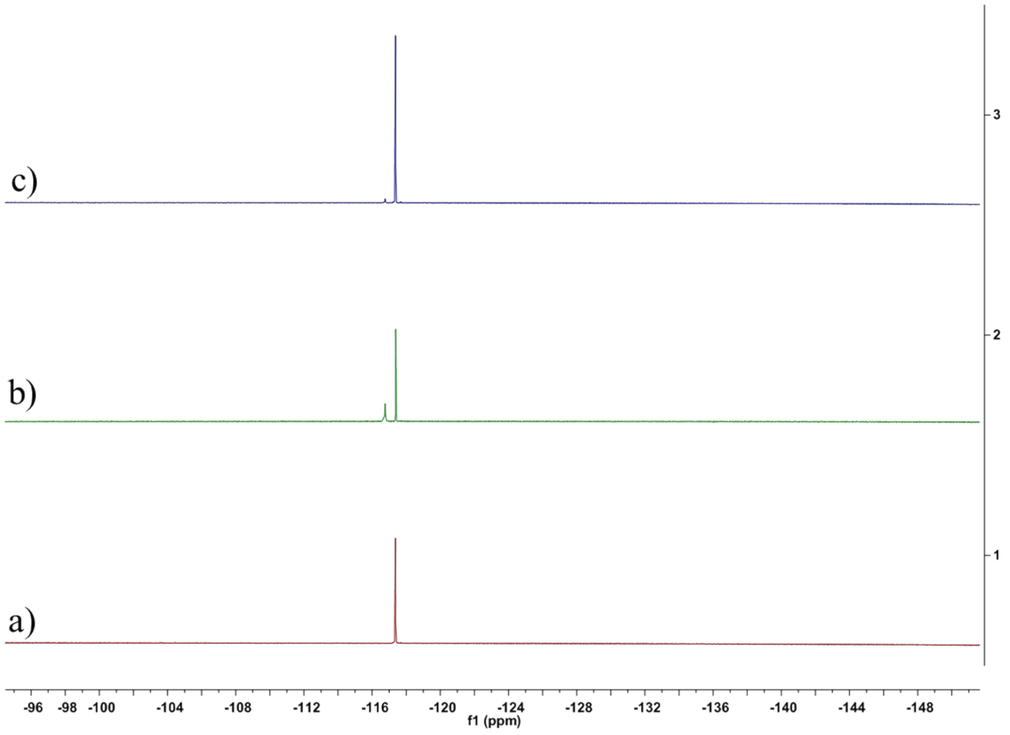


**Fig. S42.** $^{19}$F NMR spectra (564 MHz, $CDCl_3$, 298K) of a) the solution of *E*-**Azo-UPy** (10 mM) and **UPy-1** (10 mM) before irradiation, b) the $PSS_Z$ (365 nm) mixtures of **Azo-UPy** and **UPy-1**, and c) the $PSS_E$ (460 nm) mixtures of **Azo-UPy** and **UPy-1**.

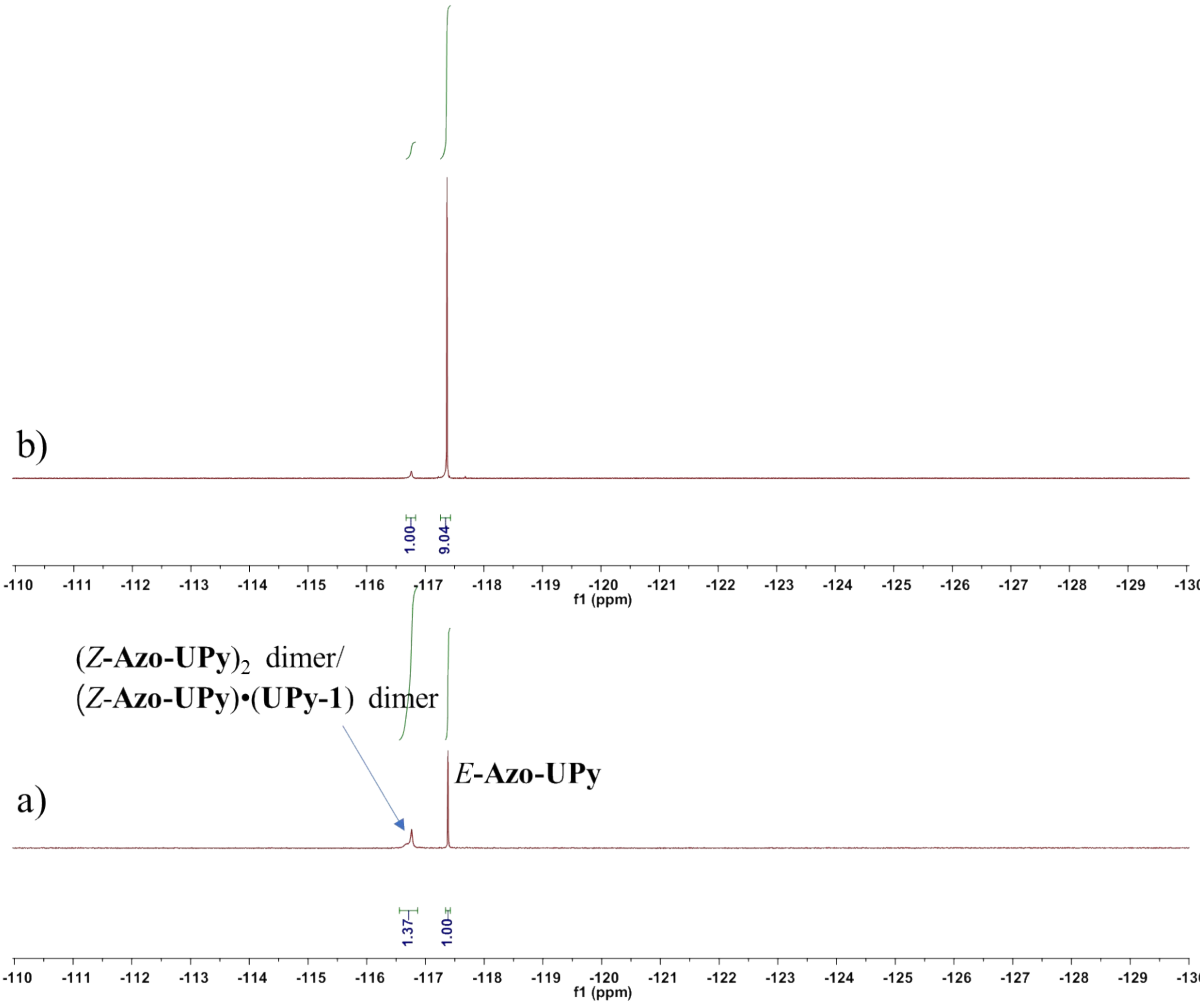


**Fig. S43.** $^{19}F$ NMR spectra (564 MHz, 10 mM, $CDCl_3$, 298K) for the solution of a) the $PSS_Z$ (365 nm) mixtures of **Azo-UPy** and he $PSS_Z$ (365 nm) mixtures of **Azo-UPy** in the presence of 1.0 equivalent of **UPy-1**.

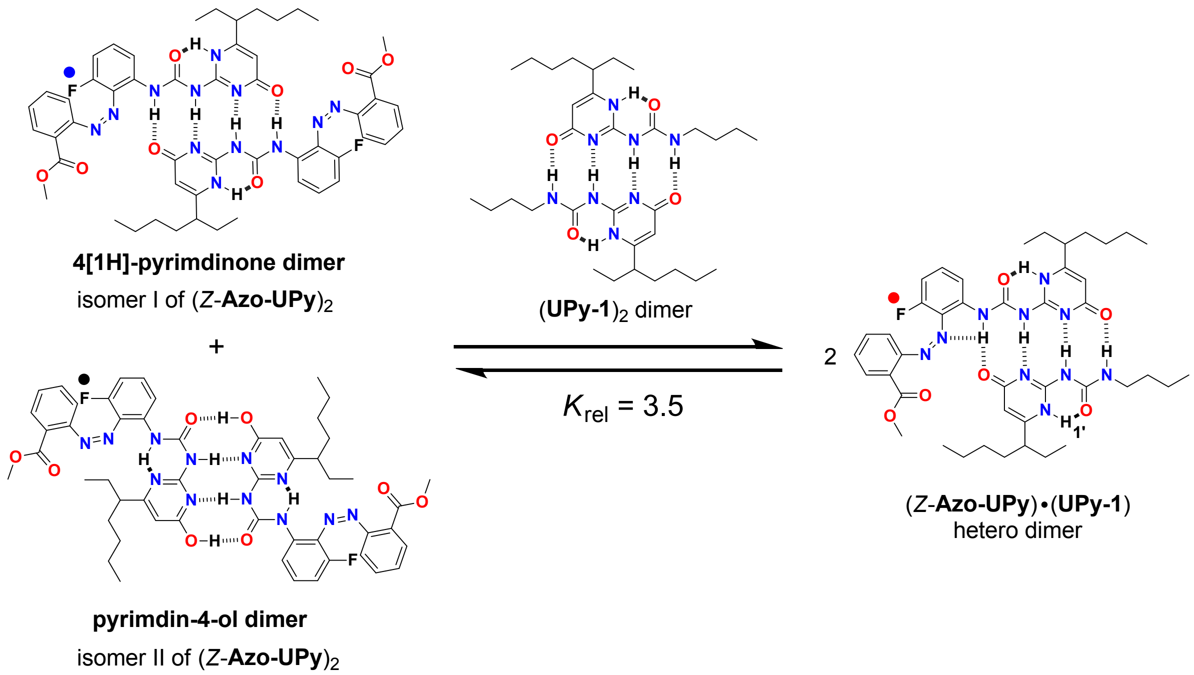


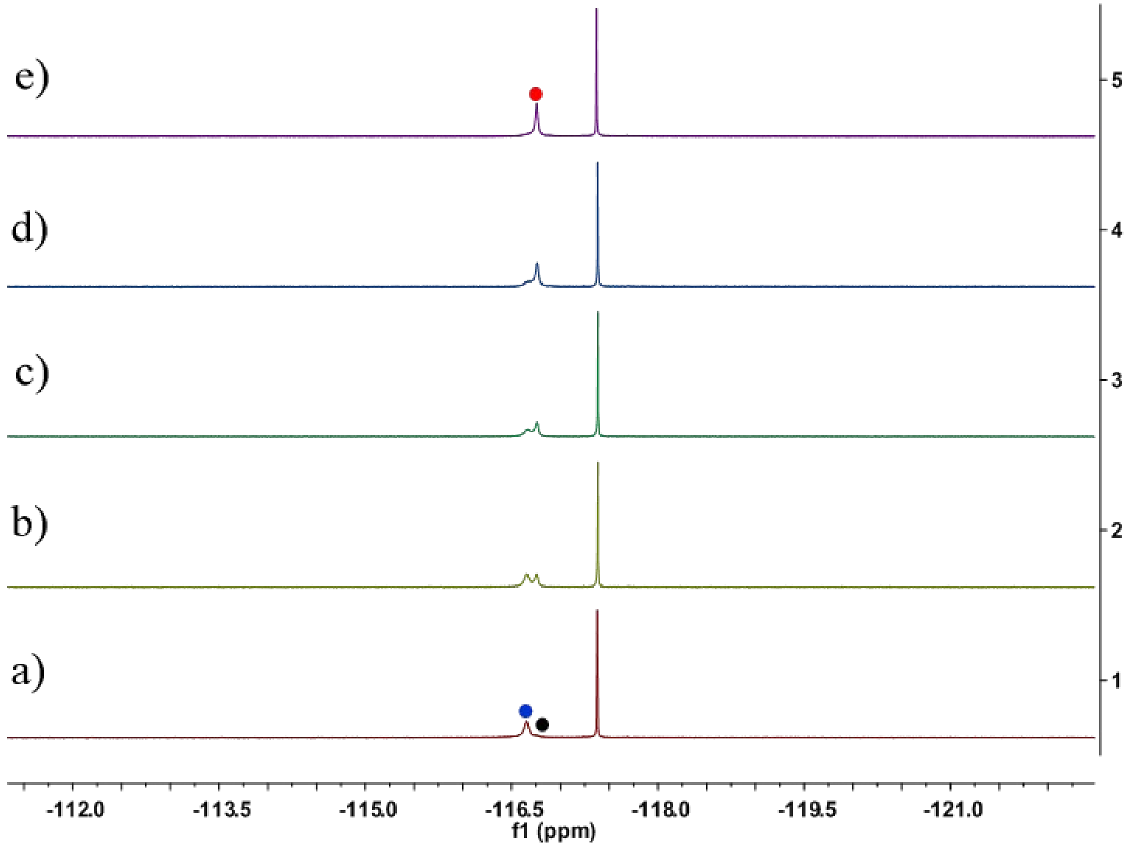


**Fig. S44.** Schematic illustration of the hetero dimerization of *Z*-**Azo-UPy** and **UPy-1**, and the $^{19}$F NMR spectra (564 MHz, 10 mM, $CDCl_3$, 298K) for the solution of the $PSS_Z$ (365 nm) mixtures of **Azo-UPy** in the presence of a) 0, b) 0.25, c) 0.50, d) 1.0, and e) 4.0 equivalent of **UPy-1**.

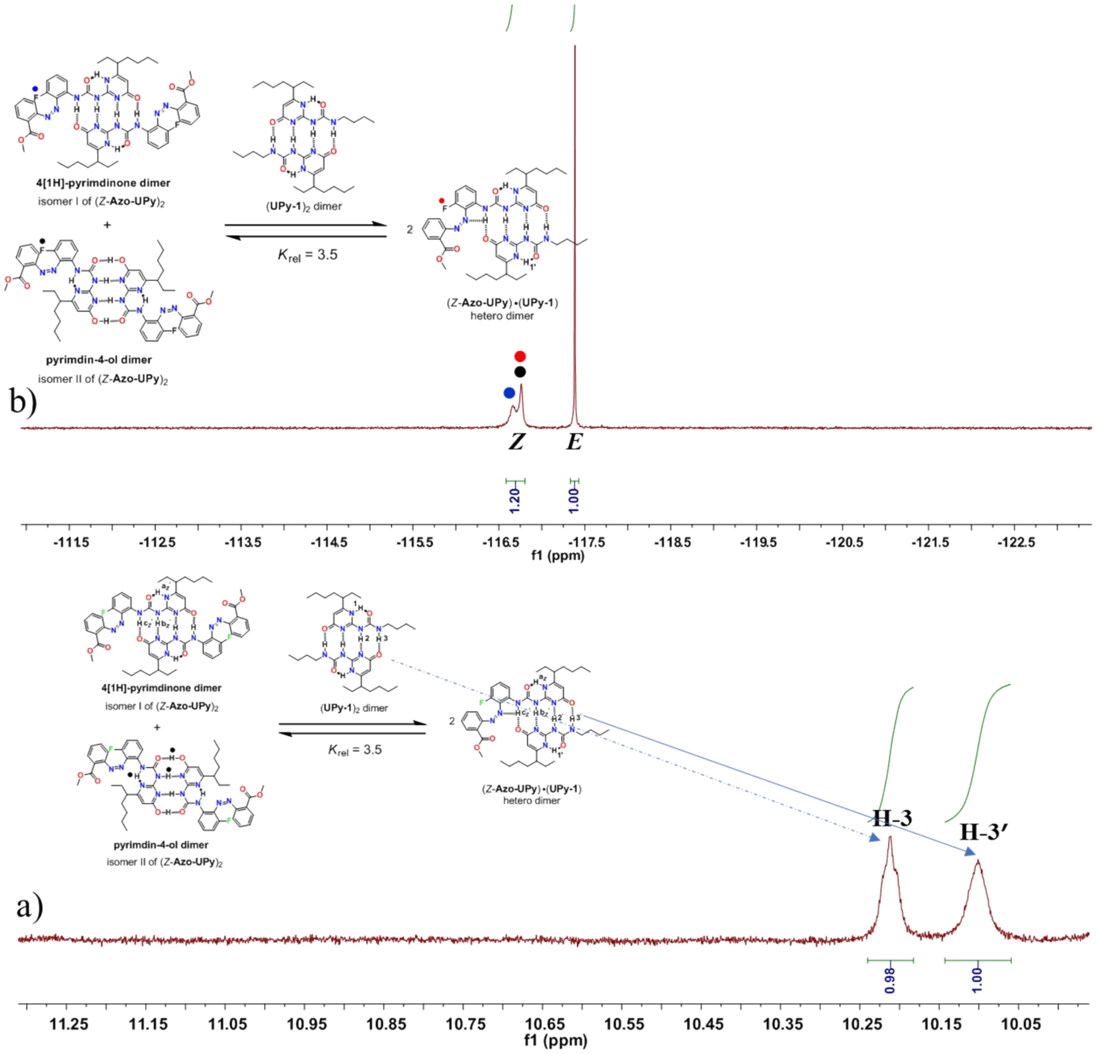


**Fig. S45.** a) Partial $^{1}H$ NMR (600 MHz, $CDCl_3$, 298K) and b) $^{19}F$ NMR (564 MHz, $CDCl_3$, 298K) spectra for the solution of the $PSS_Z$ (365 nm) mixtures of **Azo-UPy** (10 mM) in the presence of 0.50 equivalent of **UPy-1**.

The calculation for the $K_{rel}$ value based on the recorded $^{1}H$ and $^{19}F$ NMR spectra in Fig. 45:

$$C_{(Z\text{-}\mathbf{Azo\text{-}UPy})\bullet(\mathbf{UPy\text{-}1})} = 5.0\ \text{mM} \times 1/1.98 = 2.52\ \text{mM}$$

$$C_{(\mathbf{UPy\text{-}1})\bullet(\mathbf{UPy\text{-}1})} = \tfrac{1}{2}\left[C_{(\mathbf{Upy\text{-}1})} - C_{(Z\text{-}\mathbf{Azo\text{-}UPy})\bullet(\mathbf{UPy\text{-}1})}\right] = (5.0\ \text{mM} - 2.52\ \text{mM})/2 = 1.24\ \text{mM}$$

$$C_{(Z\text{-}\mathbf{Azo\text{-}UPy})} = 10\ \text{mM} \times 1.2/2.2 = 5.45\ \text{mM}$$

$$C_{(Z\text{-}\mathbf{Azo\text{-}UPy})\bullet(Z\text{-}\mathbf{Azo\text{-}UPy})} = \tfrac{1}{2}\left[C_{(Z\text{-}\mathbf{Azo\text{-}UPy})} - C_{(Z\text{-}\mathbf{Azo\text{-}UPy})\bullet(\mathbf{UPy\text{-}1})}\right] = 0.5\times(5.45\ \text{mM} - 2.52\ \text{mM}) = 1.47\ \text{mM}$$

$$K_{rel} = C^2_{(Z\text{-}\mathbf{Azo\text{-}UPy})\bullet(\mathbf{UPy\text{-}1})} / \left[C_{(Z\text{-}\mathbf{Azo\text{-}UPy})\bullet(Z\text{-}\mathbf{Azo\text{-}UPy})} \times C_{(\mathbf{UPy\text{-}1})\bullet(\mathbf{UPy\text{-}1})}\right] = (2.52\ \text{mM})^2 / (1.47\ \text{mM} \times 1.24\ \text{mM}) = 3.48$$

## Section 6: Application of Azo-UPy for photocontrollable macro-/molecular self-assembly

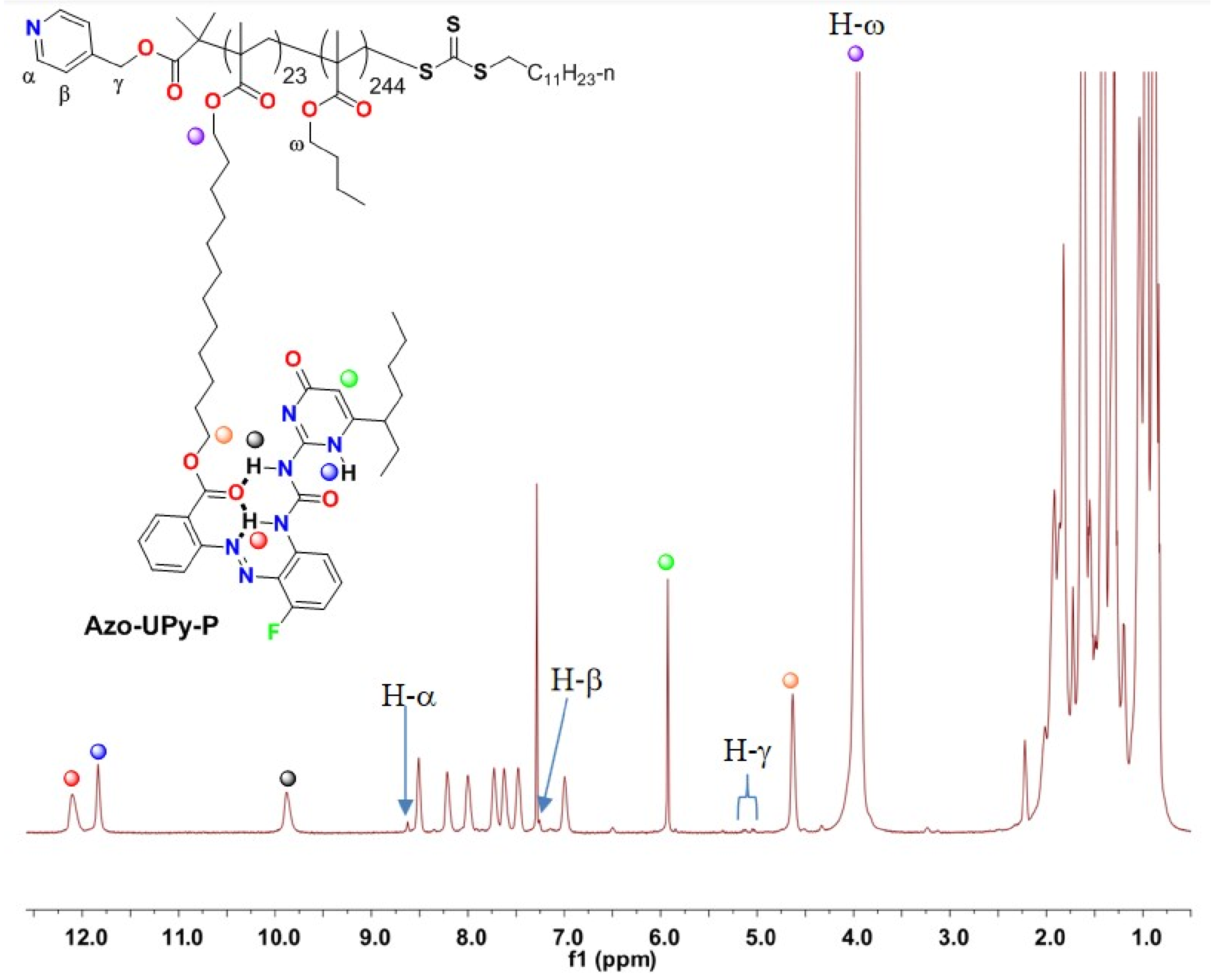


**Fig. S46.** $^{1}$H NMR spectrum (600 MHz, 40 mg / mL, $CDCl_3$, 298K) of the solution of polymer **Azo-UPy-P** in the pristine state.

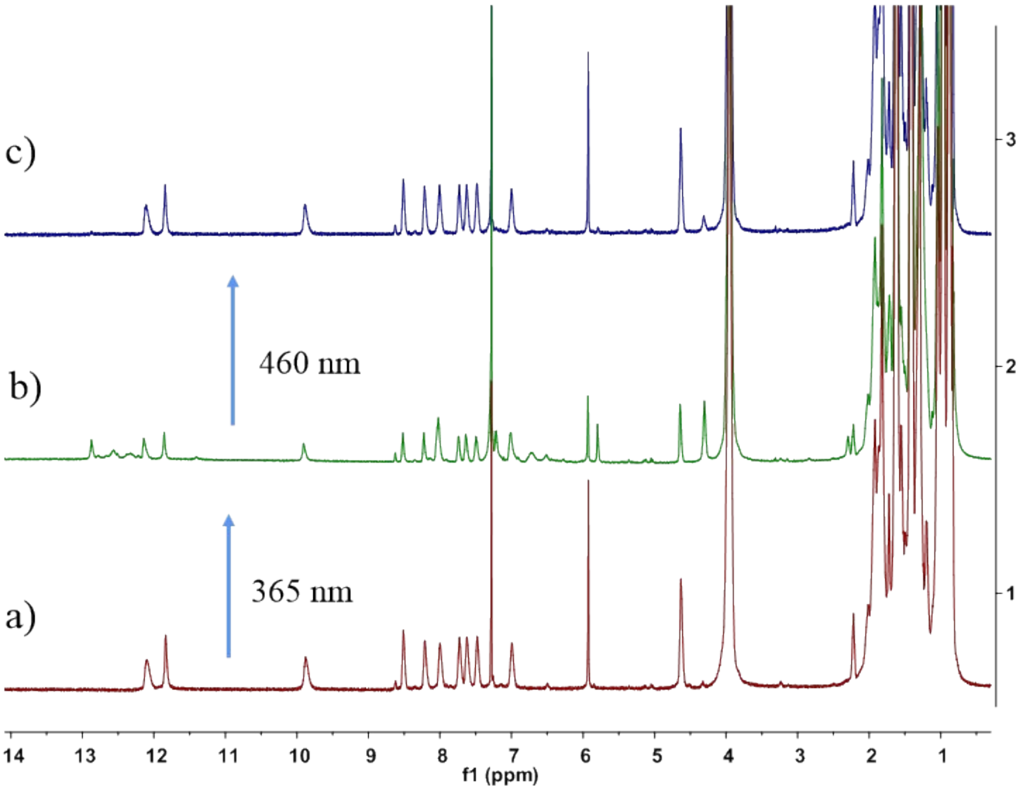


**Fig. S47.** $^{1}$H NMR spectra (600 MHz, 40 mg / mL, $CDCl_3$, 298K) of the solution of a) polymer **Azo-UPy-P** in the pristine state, b) the $PSS_Z$ (365 nm) mixtures of polymer **Azo-UPy-P**, and c) the $PSS_E$ (460 nm) mixtures of polymer **Azo-UPy-P**.

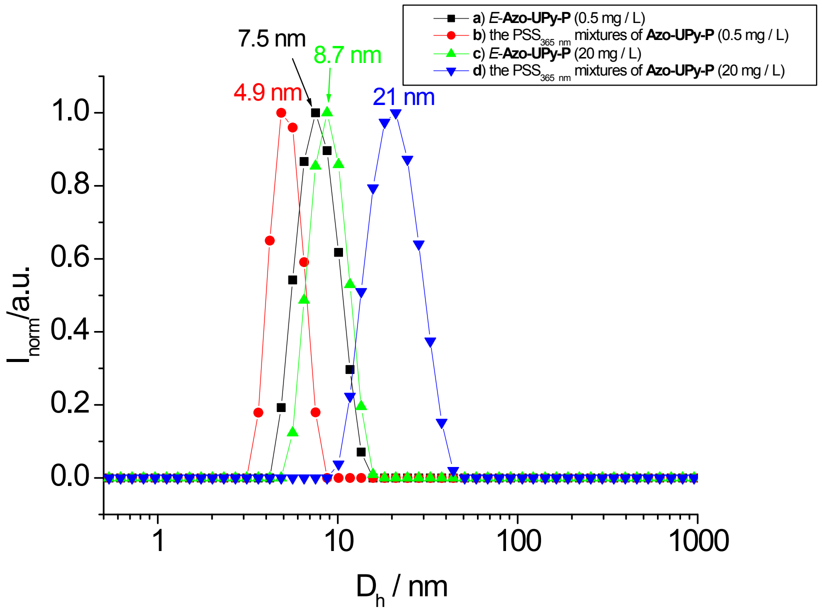


**Fig. S48.** DLS profiles of polymer *E*-**Azp-UPy-P** (0.5 mg / mL) (black), the $PSS_Z$(365 nm) mixtures of polymer *E*-**Azp-UPy-P** (0.5 mg / mL) (red), polymer *E*-**Azp-UPy-P** (20 mg / mL) (green) and the $PSS_Z$(365 nm) mixtures of polymer *E*-**Azp-UPy-P** (20 mg / mL) (blue) in $CHCl_3$.

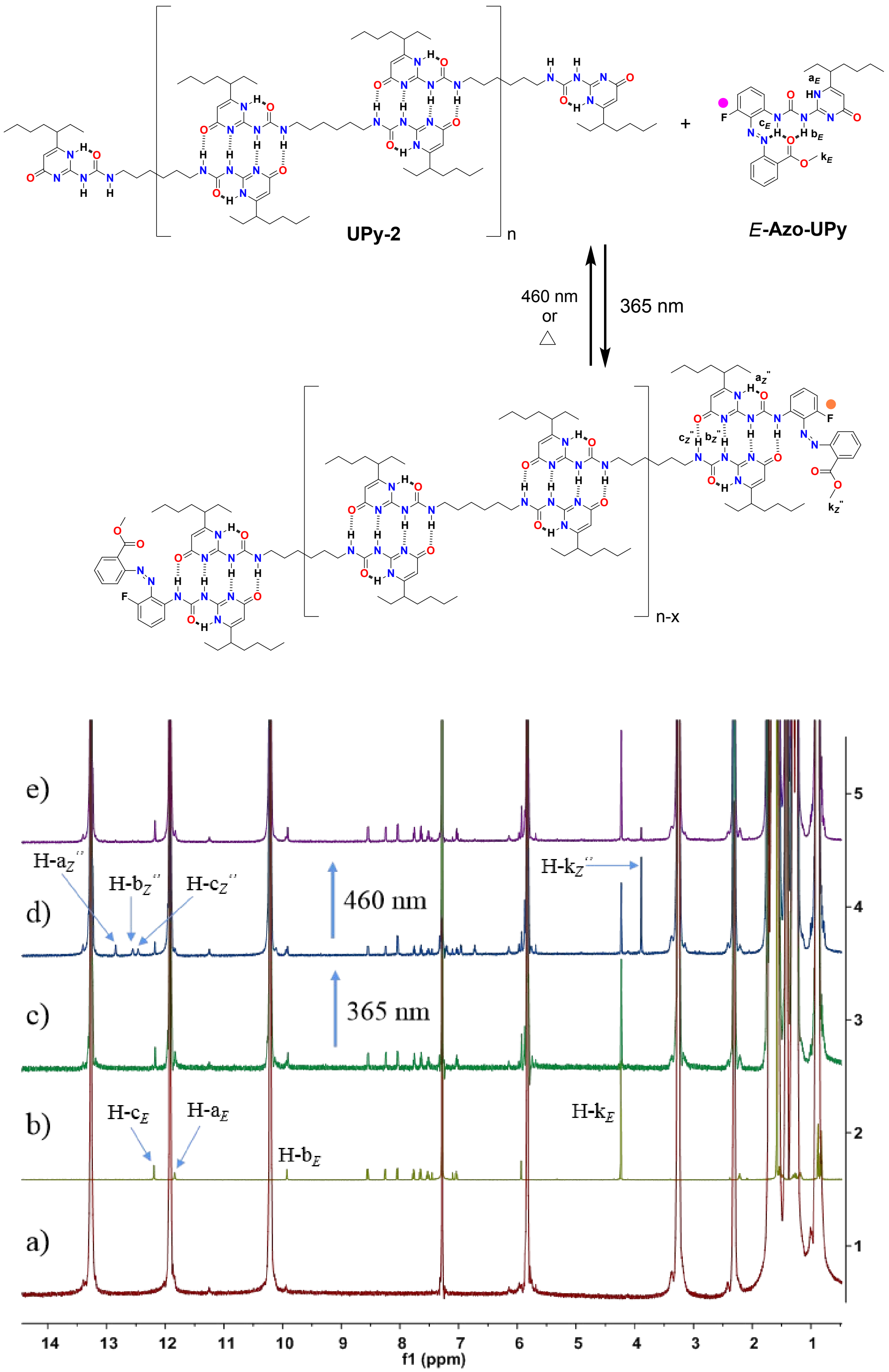


**Fig. S49.** Schematic illustration of the photoregulated supramolecular polymerization, and the $^{1}H$ NMR spectra (600 MHz, $CDCl_3$, 298K) of the solution of a) **UPy-2** (40 mM), b) *E*-**Azo-UPy** (1.0 mM), c) **UPy-2** (40 mM) and *E*-**Azo-UPy** (1.0 mM), d) the $PSS_Z$ (365 nm) mixtures of **Azo-UPy** (1.0 mM) and **UPy-2** (40 mM), and e) the $PSS_E$ (460 nm) mixtures of **Azo-UPy** (1.0 mM) and **UPy-2** (40 mM).

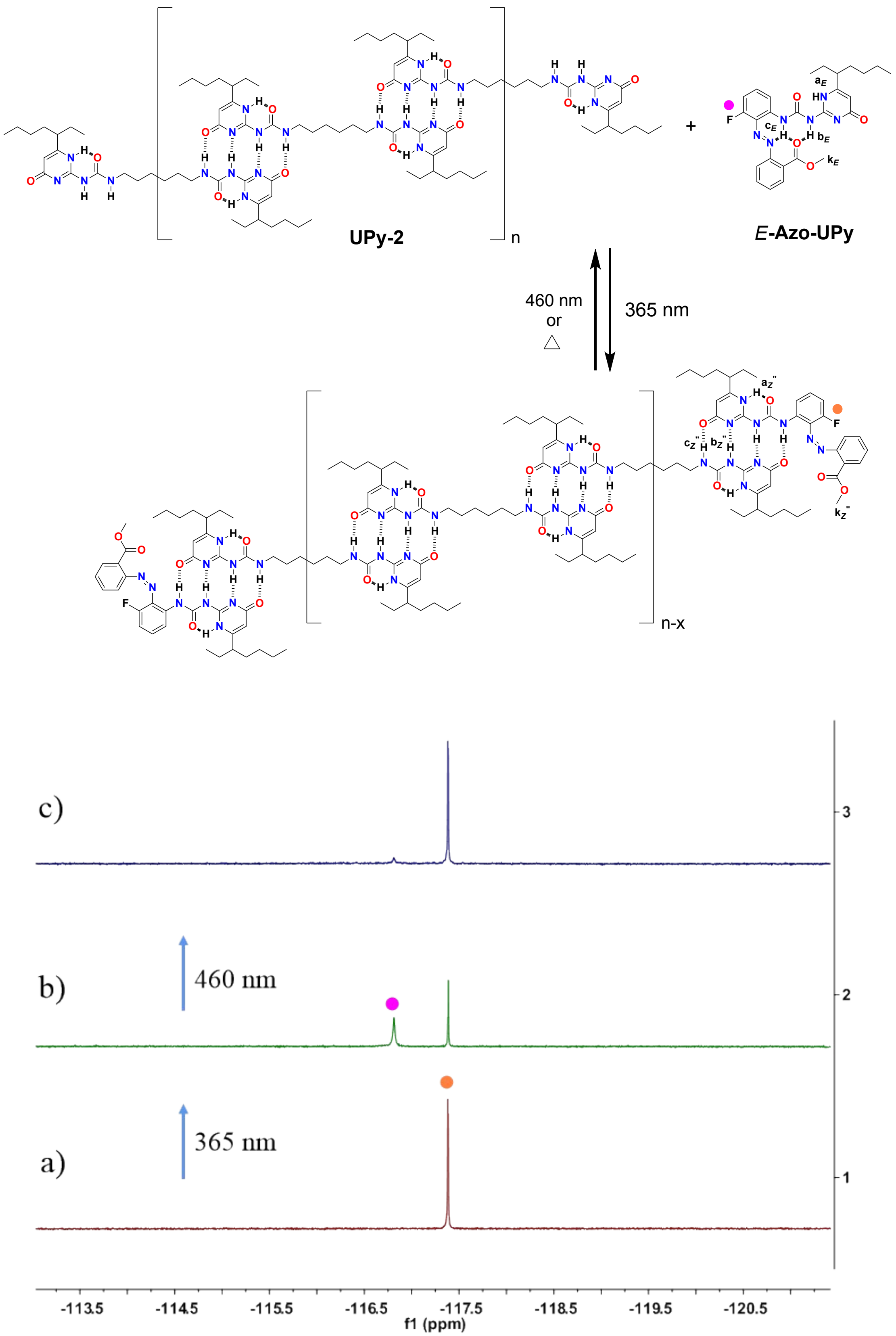


**Fig. S50.** Schematic illustration of the photoregulated supramolecular polymerization, and the $^{19}$F NMR spectra (564 MHz, CDCl3, 298K) of the solution of a) **UPy-2** (40 mM) and *E*-**Azo-UPy** (1.0 mM), b) the PSS$_Z$ (365 nm) mixtures of **Azo-UPy** (1.0 mM) and **UPy-2** (40 mM), and c) the PSS$_E$ (460 nm) mixtures of **Azo-UPy** (1.0 mM) and **UPy-2** (40 mM).

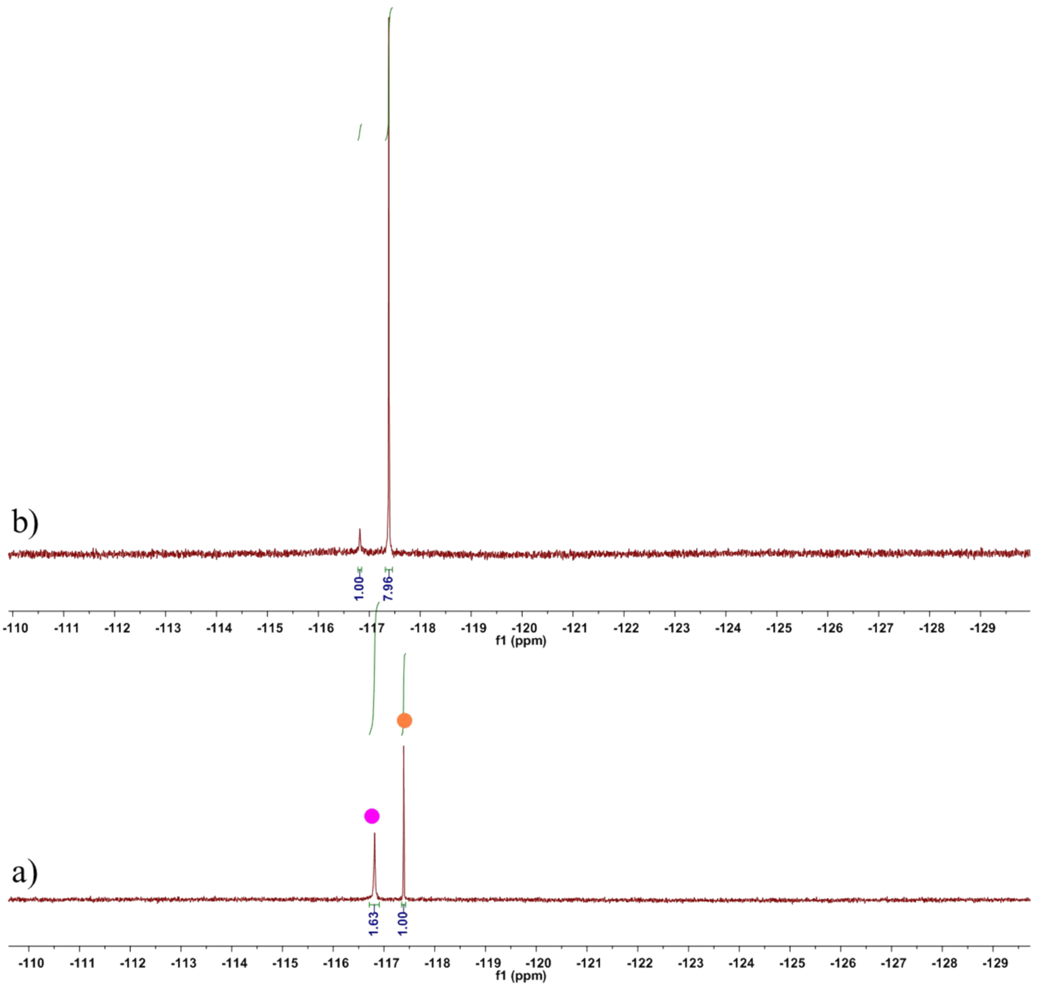


**Fig. S51.** $^{19}$F NMR spectra (564 MHz, $CDCl_3$, 298K) of the solution of a)) the $PSS_Z$ (365 nm) mixtures of **Azo-UPy** (1.0 mM) and **UPy-2** (40 mM), and b) the $PSS_E$ (460 nm) mixtures of **Azo-UPy** (1.0 mM) and **UPy-2** (40 mM).

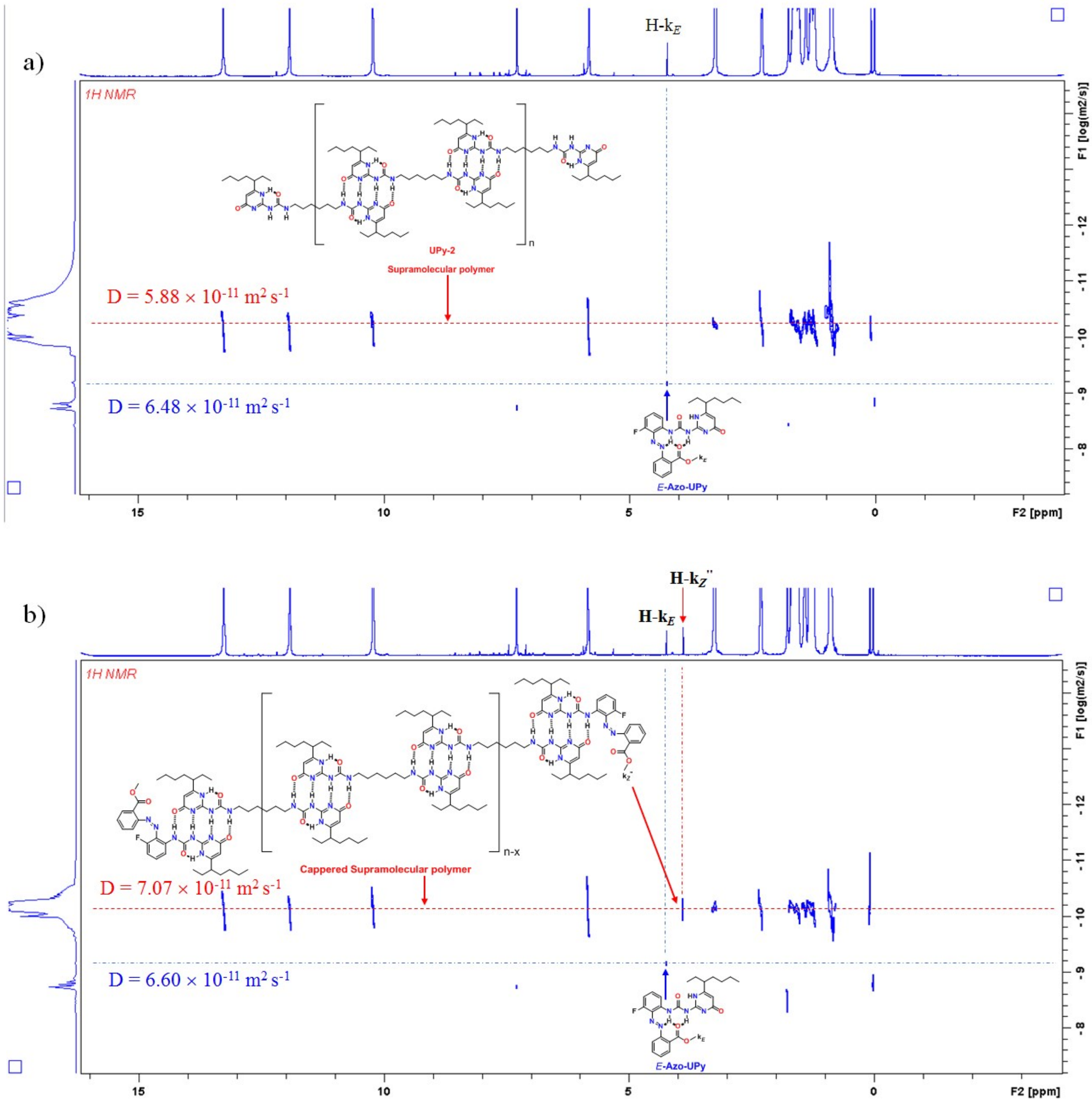


**Fig. S52.** DOSY-NMR spectra (600 MHz, 40 mM, $CDCl_3$, 298K) of a) **UPy-2** (40 mM) and *E*-**Azo-UPy** (1.0 mM), and b) the $PSS_Z$ (365 nm) mixtures of **Azo-UPy** (1.0 mM) and **UPy-2** (40 mM).

## Section 7: $^{1}H$ and $^{13}C$ NMR spectra for new compounds

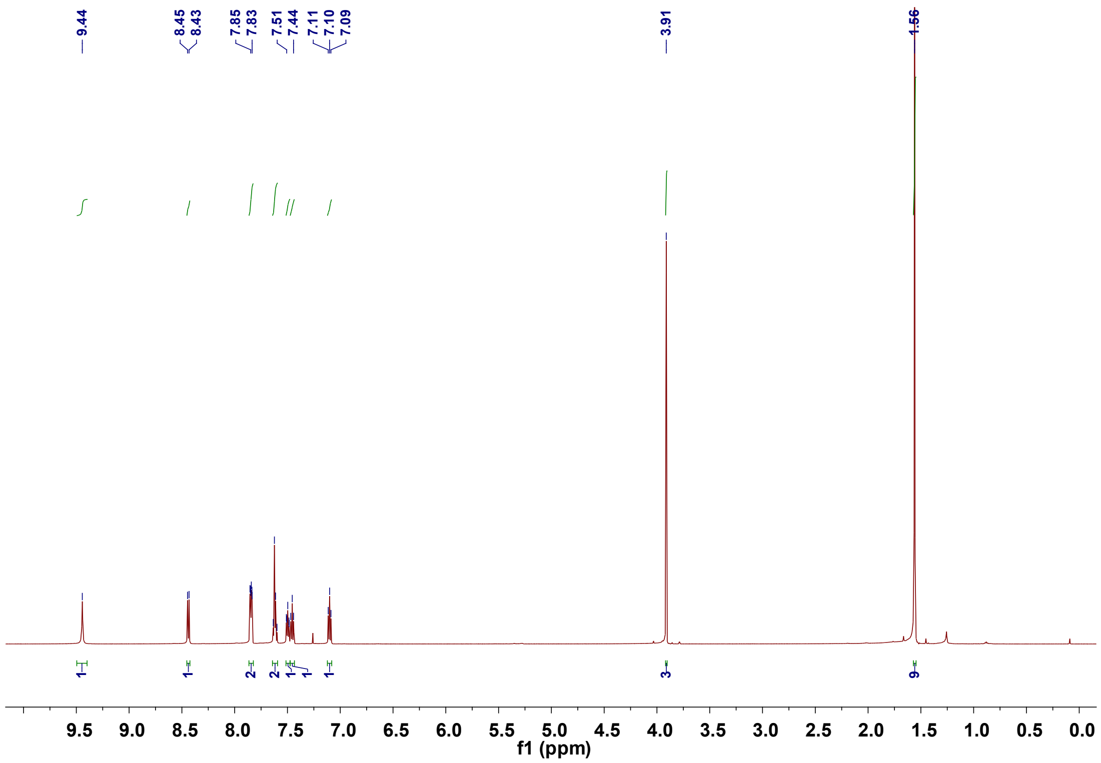


$^{1}H$ NMR spectrum (600 MHz, $CDCl_3$, 298 K) of compound **1a**.

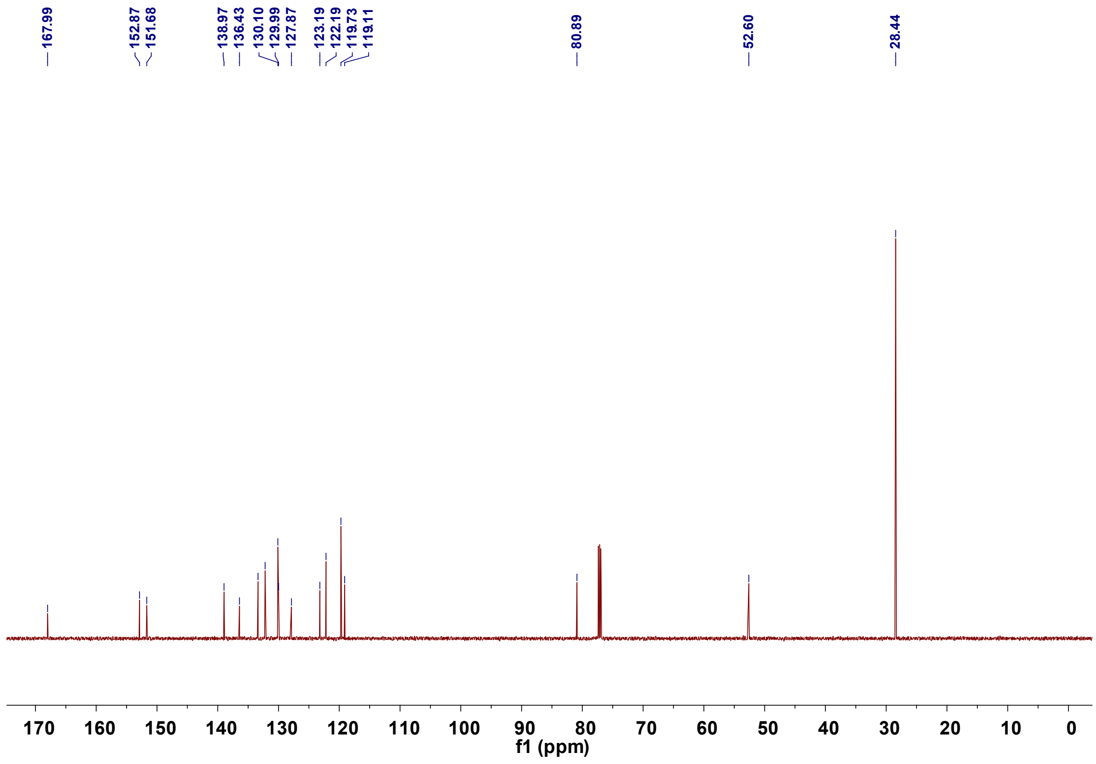

$^{13}$C NMR spectrum (150 MHz, CDCl$_3$, 298 K) of compound **1a**.

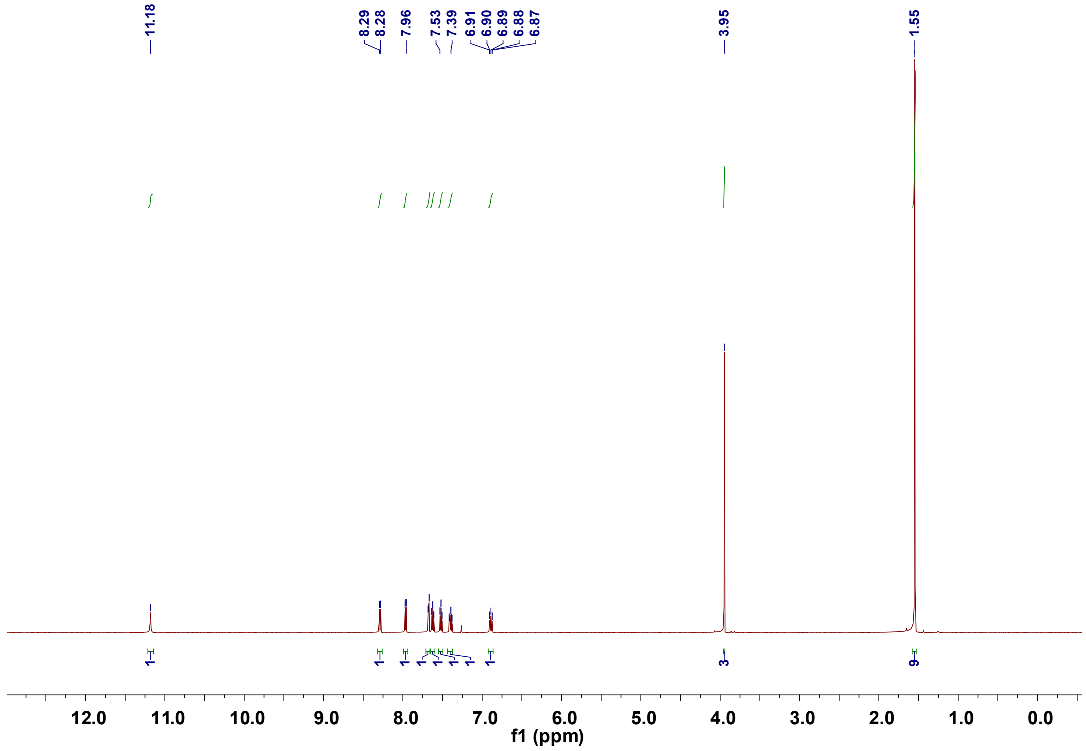


$^{1}$H NMR spectrum (600 MHz, CDCl$_3$, 298 K) of compound **1b**.

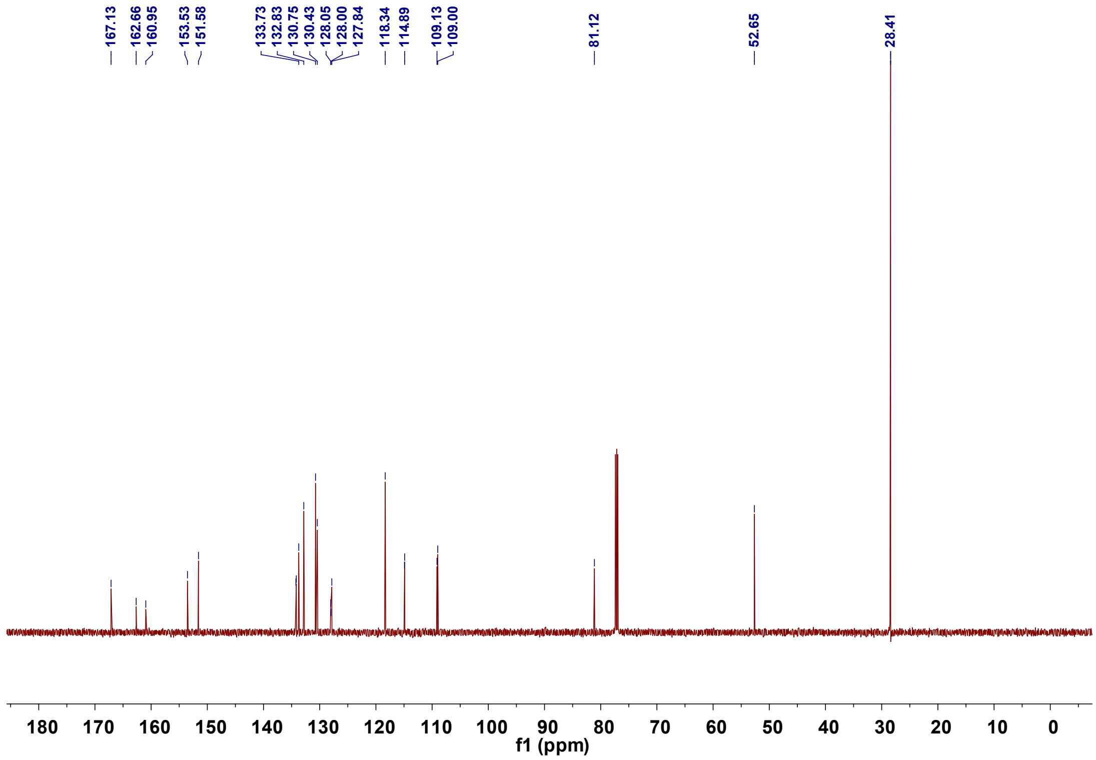


$^{13}$C NMR spectrum (150 MHz, CDCl$_3$, 298 K) of compound **1b**.

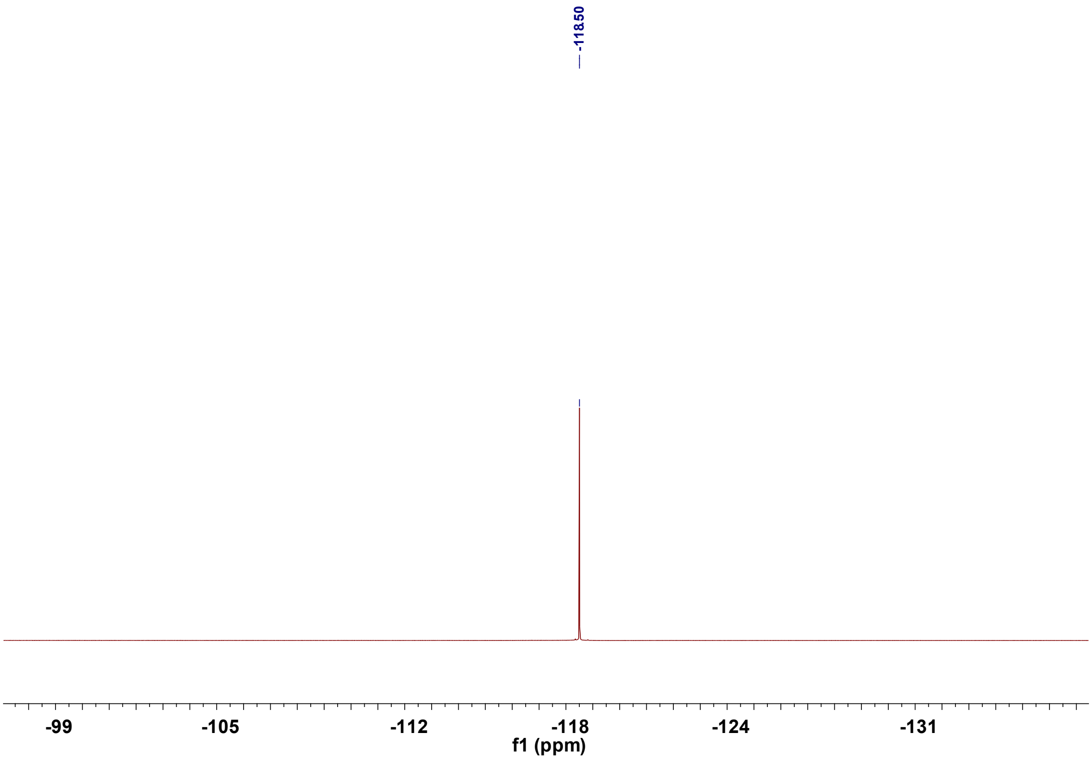


$^{19}F$ NMR spectrum (564 MHz, $CDCl_3$, 298 K) of compound **1b**.

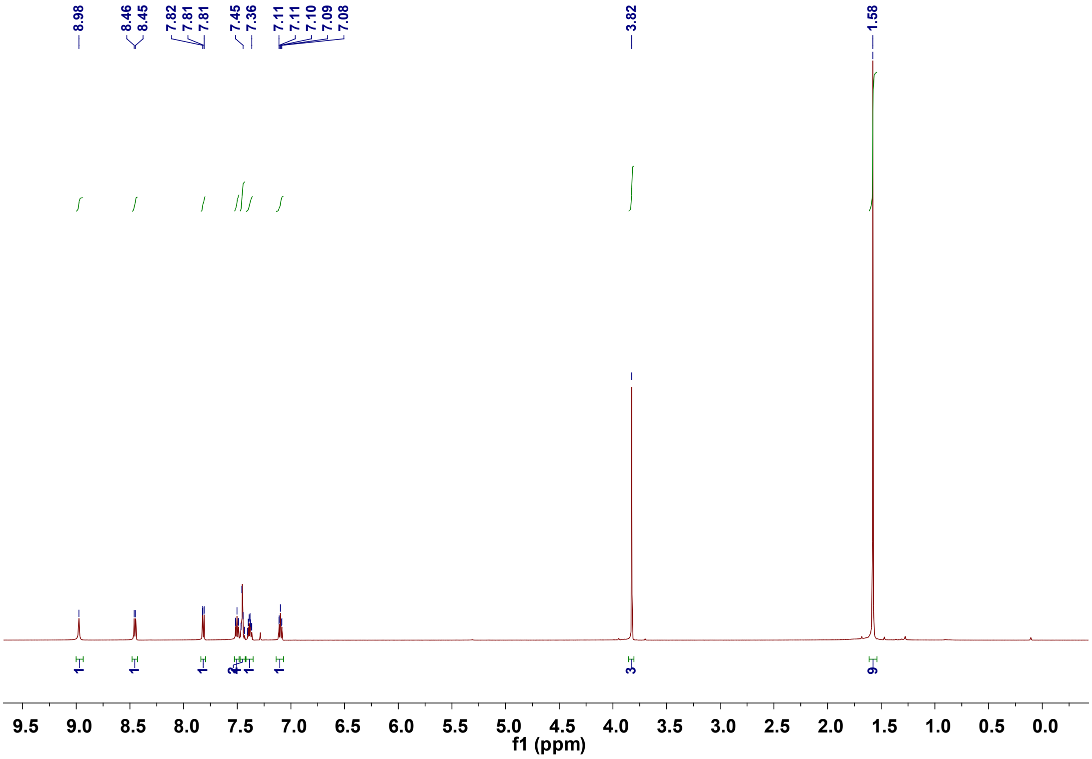


$^{1}H$ NMR spectrum (600 MHz, $CDCl_3$, 298 K) of compound **1c**.

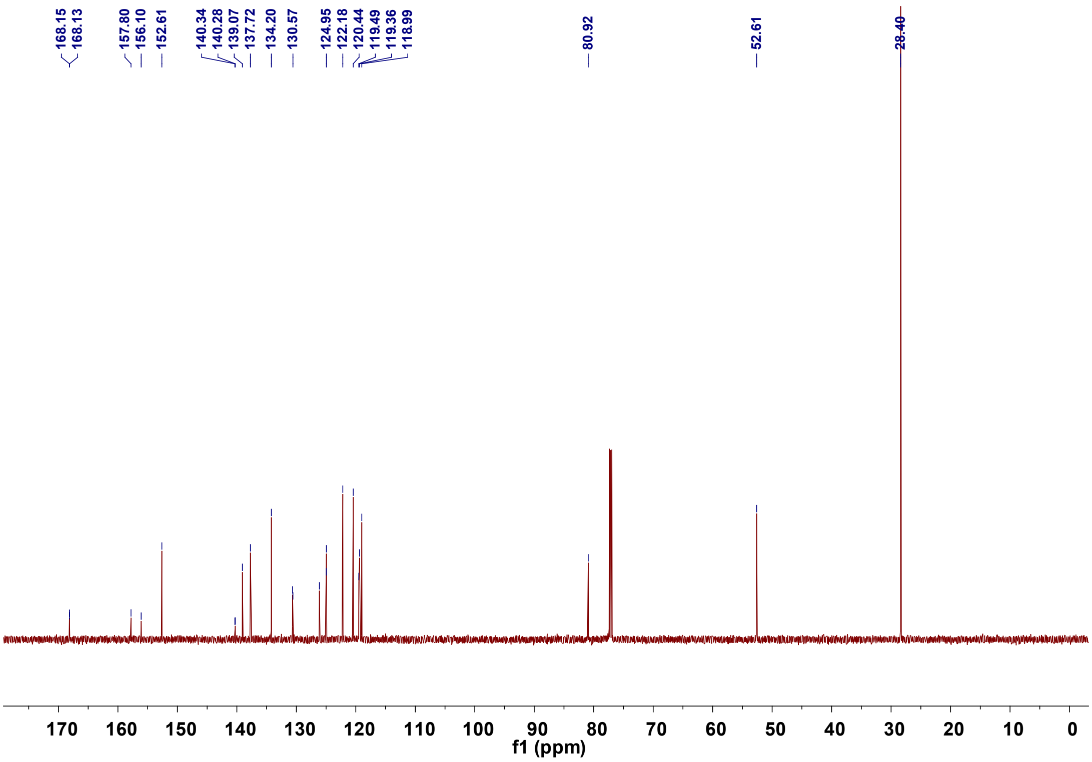


$^{13}C$ NMR spectrum (150 MHz, $CDCl_3$, 298 K) of compound **1c**.

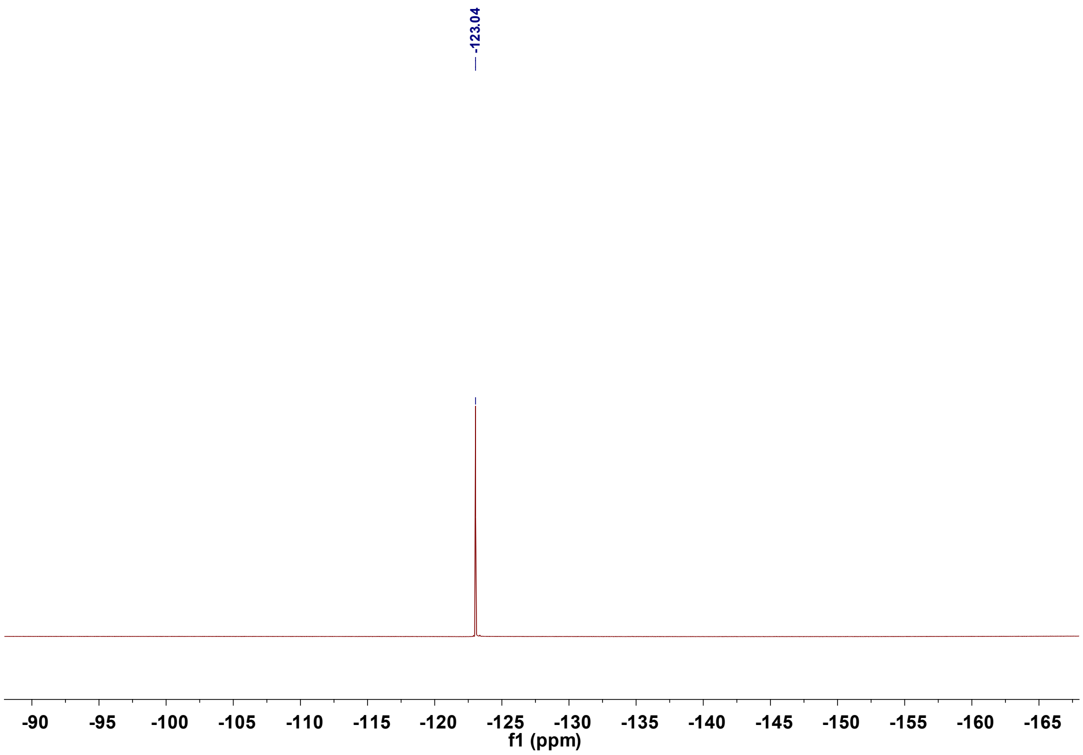


$^{19}F$ NMR spectrum (564 MHz, $CDCl_3$, 298 K) of compound **1c**.

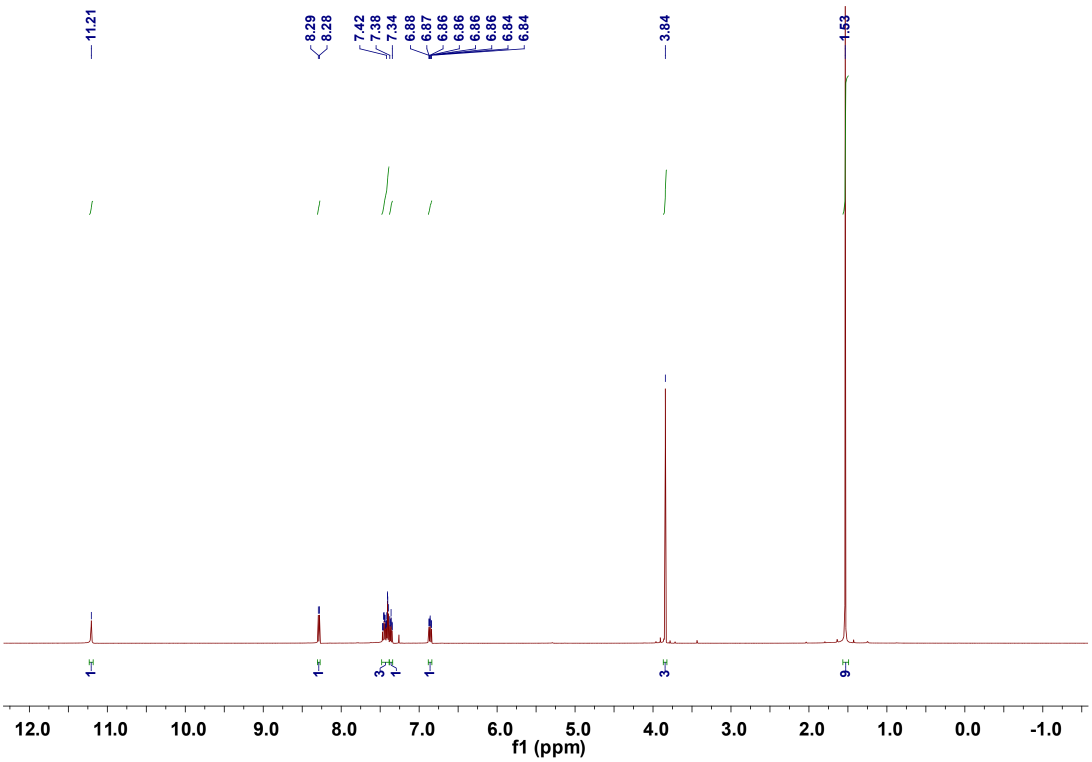


$^{1}$H NMR spectrum (600 MHz, $CDCl_3$, 298 K) of compound **1d**.

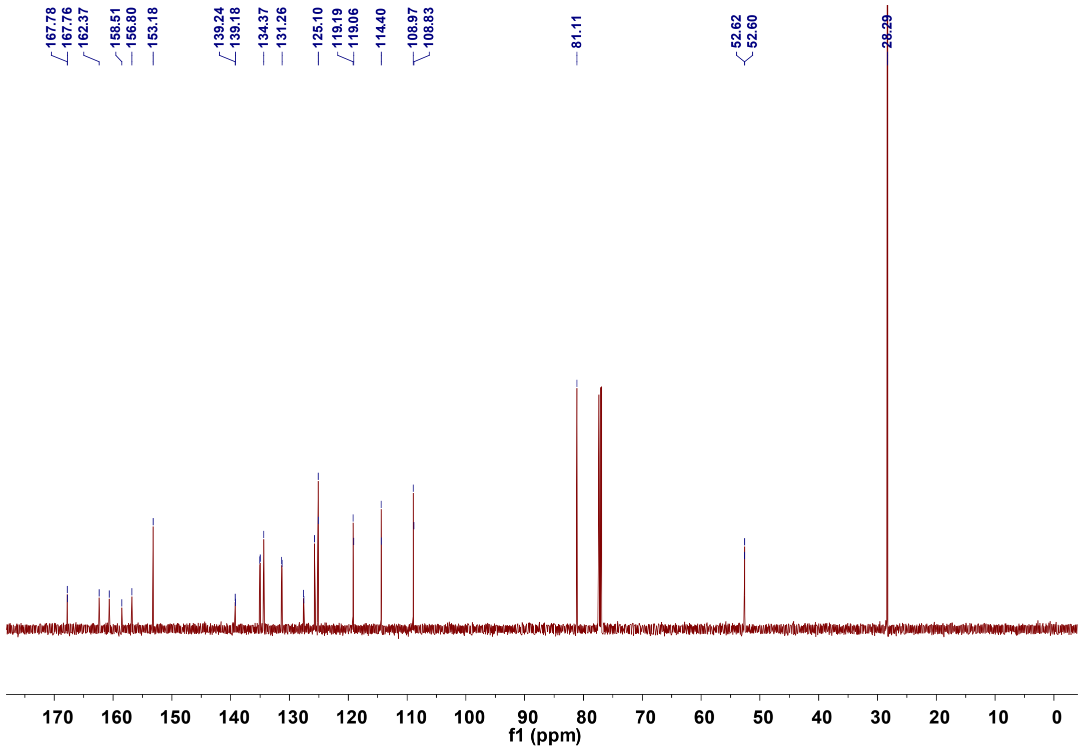


$^{13}$C NMR spectrum (150 MHz, $CDCl_3$, 298 K) of compound **1d**.

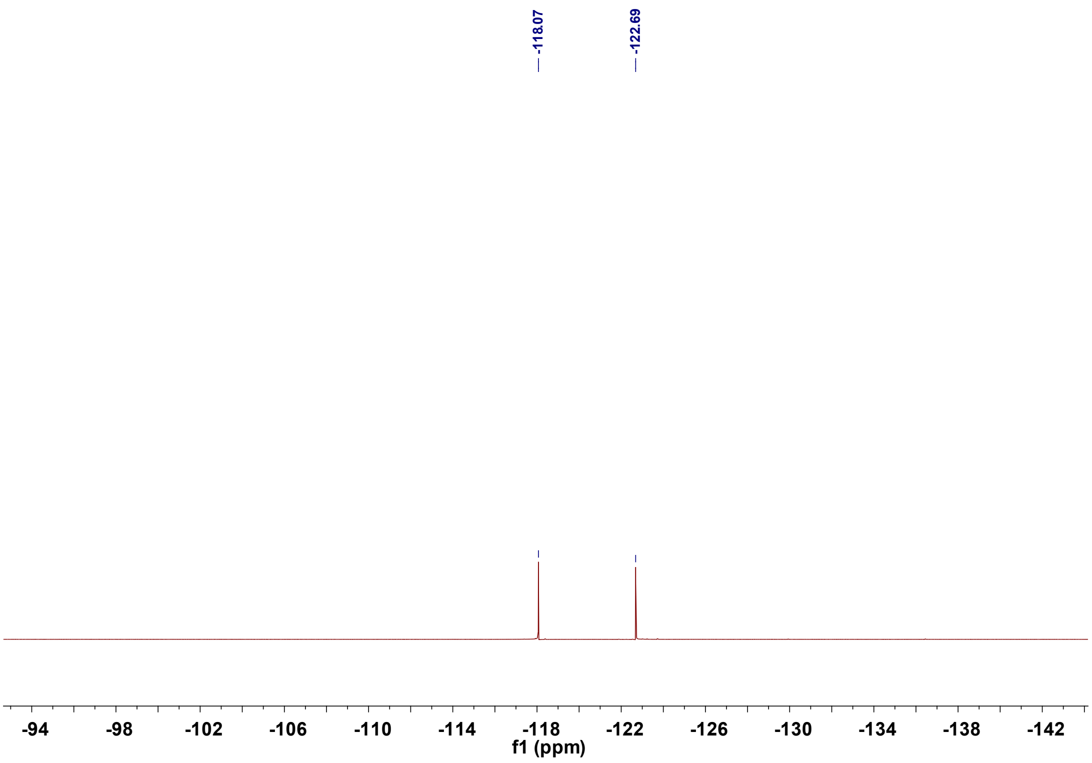


$^{19}$F NMR spectrum (564 MHz, $CDCl_3$, 298 K) of compound **1d**.

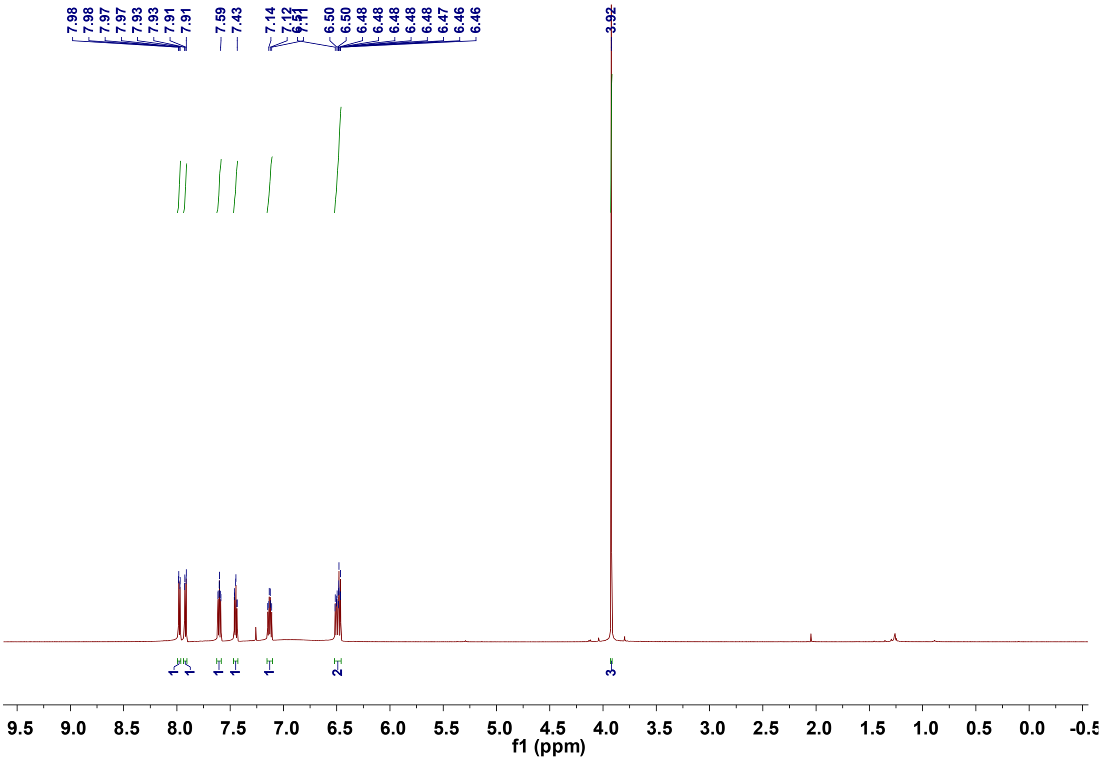


$^{1}$H NMR spectrum (600 MHz, $CDCl_3$, 298 K) of compound **7**.

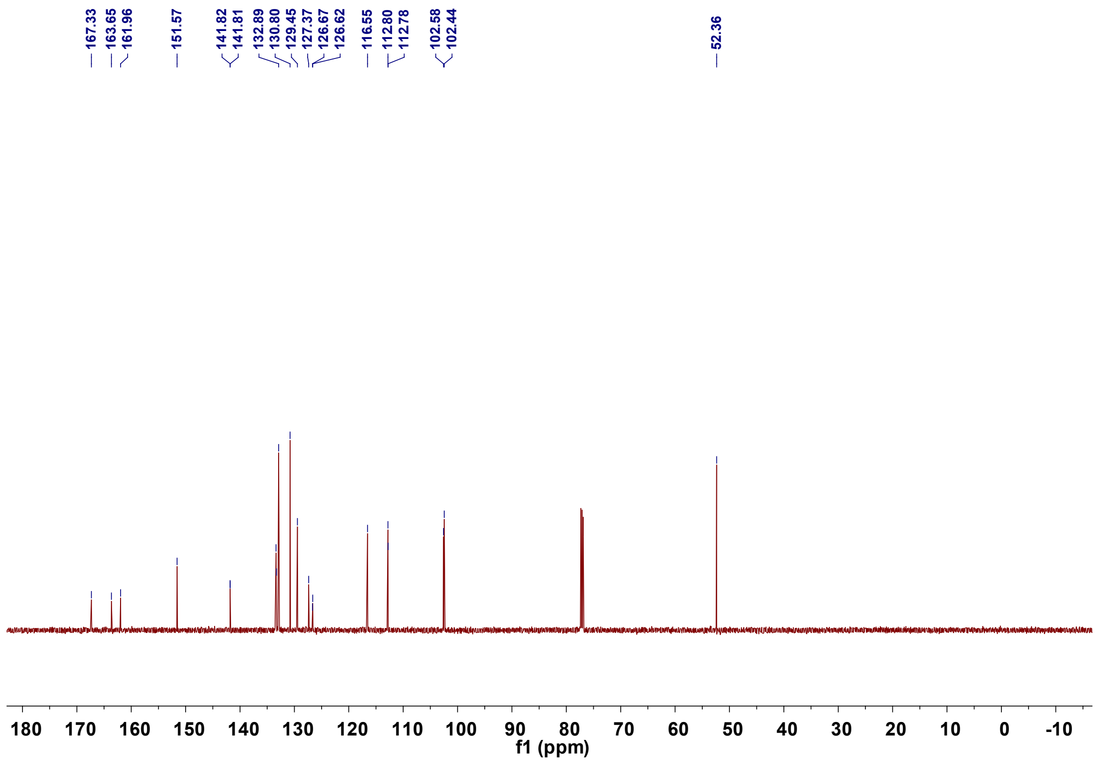


$^{13}$C NMR spectrum (150 MHz, $CDCl_3$, 298 K) of compound **7**.

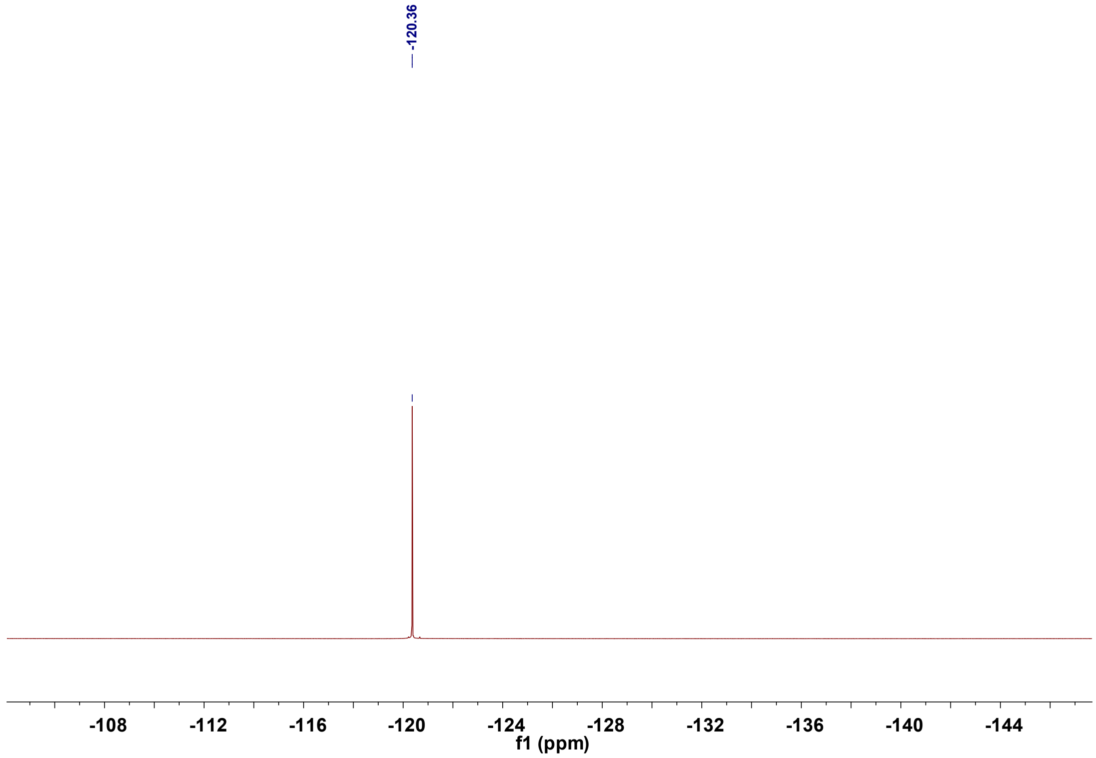


$^{19}$F NMR spectrum (564 MHz, $CDCl_3$, 298 K) of compound **7**.

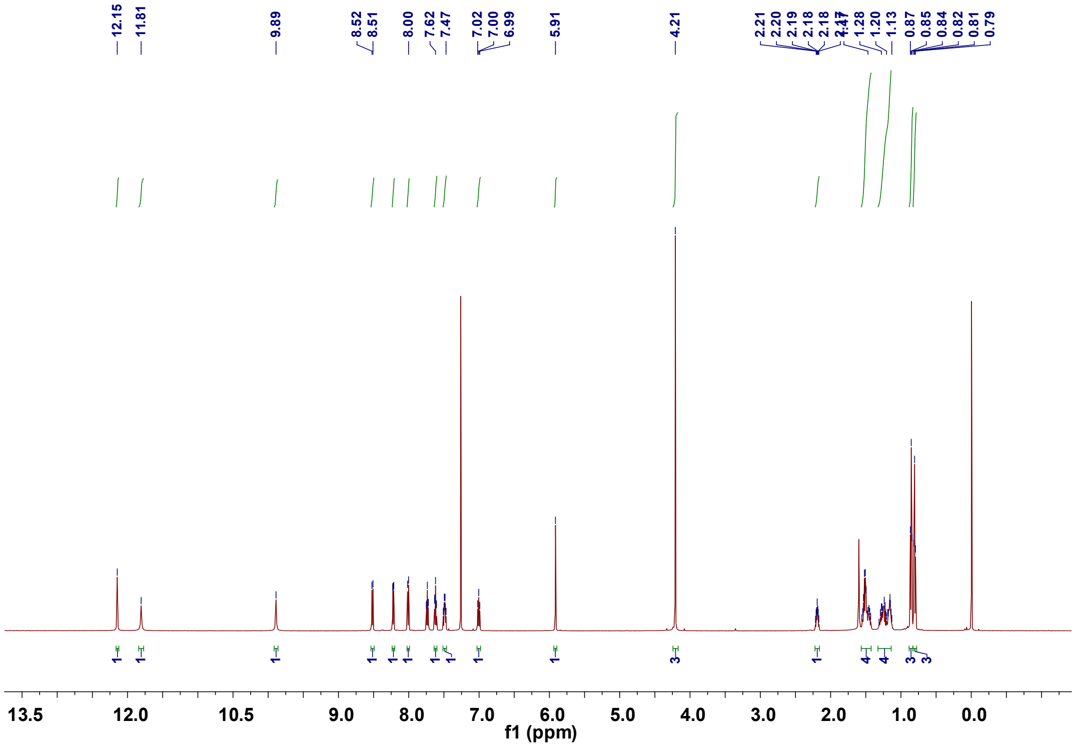


$^{1}$H NMR spectrum (600 MHz, $CDCl_3$, 298 K) of **Azo-UPy**.

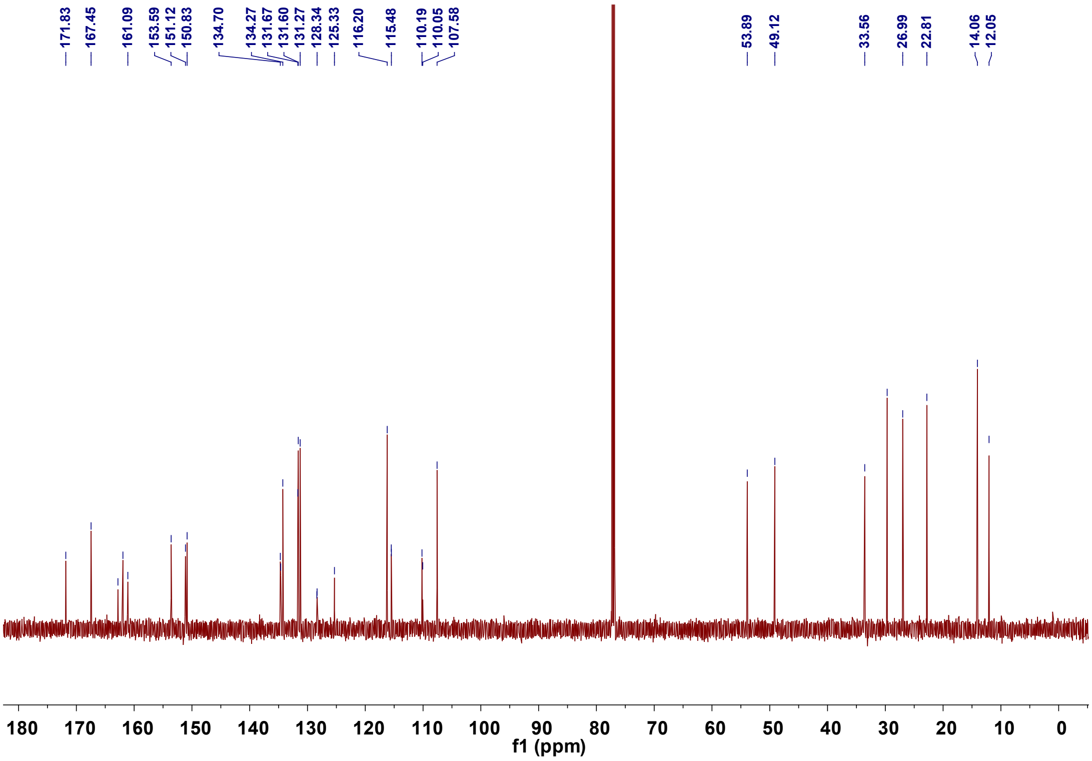


$^{13}$C NMR spectrum (150 MHz, $CDCl_3$, 298 K) of **Azo-UPy**.

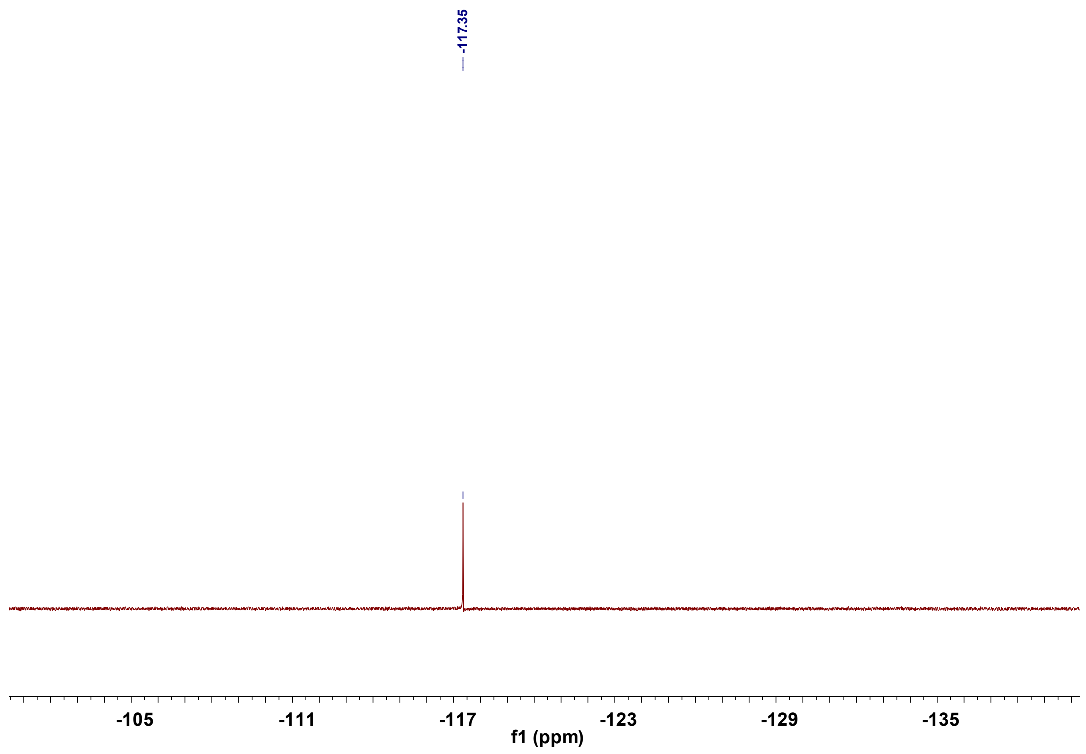

$^{19}$F NMR spectrum (564 MHz, $CDCl_3$, 298 K) of **Azo-UPy**.

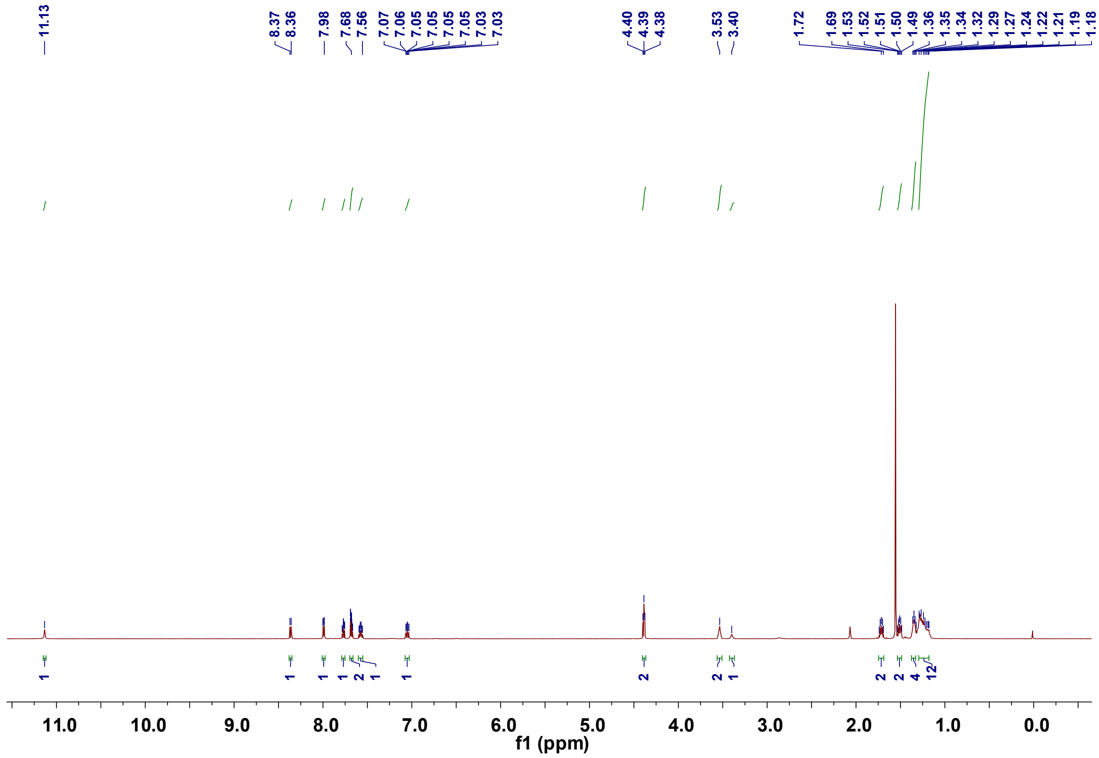

$^{1}$H NMR spectrum (600 MHz, $CD_3COD_3$, 298 K) of compound **9**.

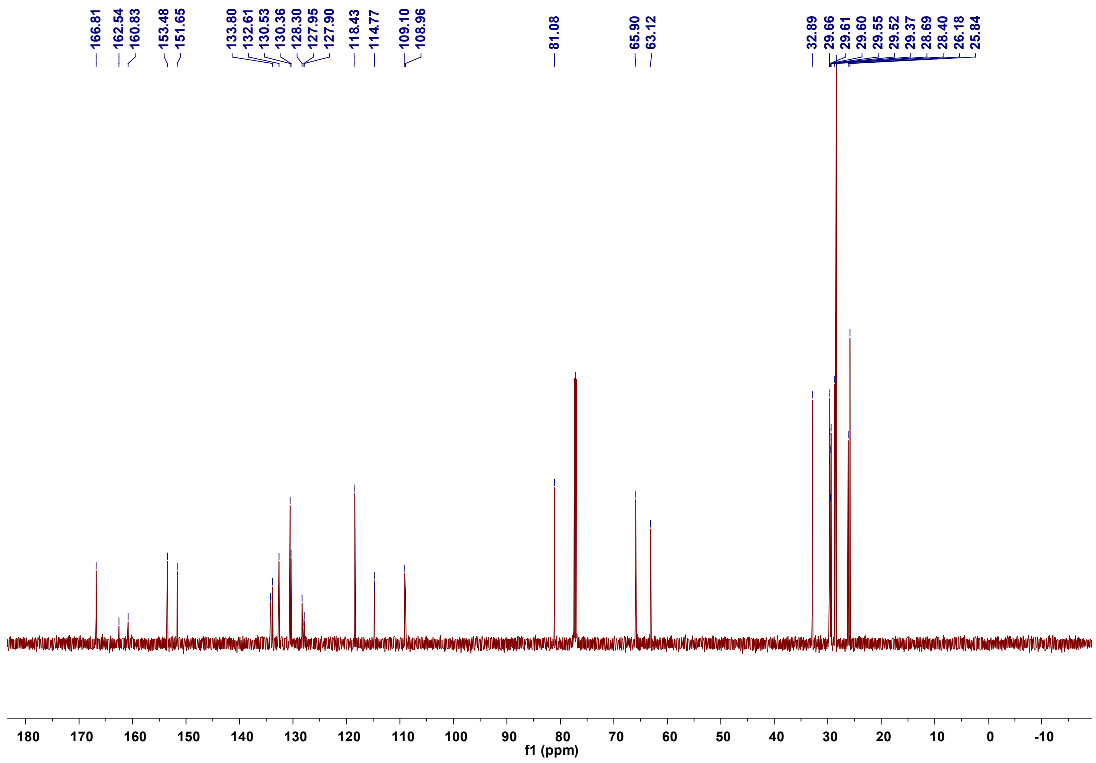


$^{13}C$ NMR spectrum (150 MHz, $CDCl_3$, 298 K) of compound **9**.

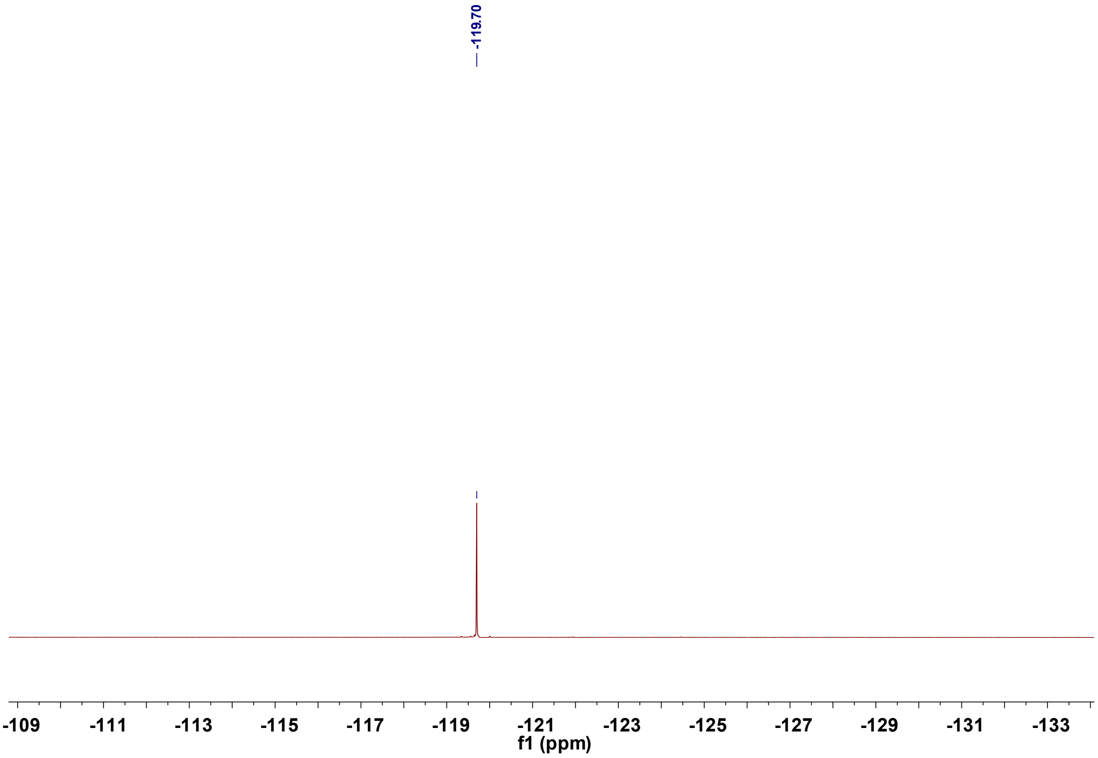


$^{19}F$ NMR spectrum (564 MHz, $CD_3COCD_3$, 298 K) of compound **9**.

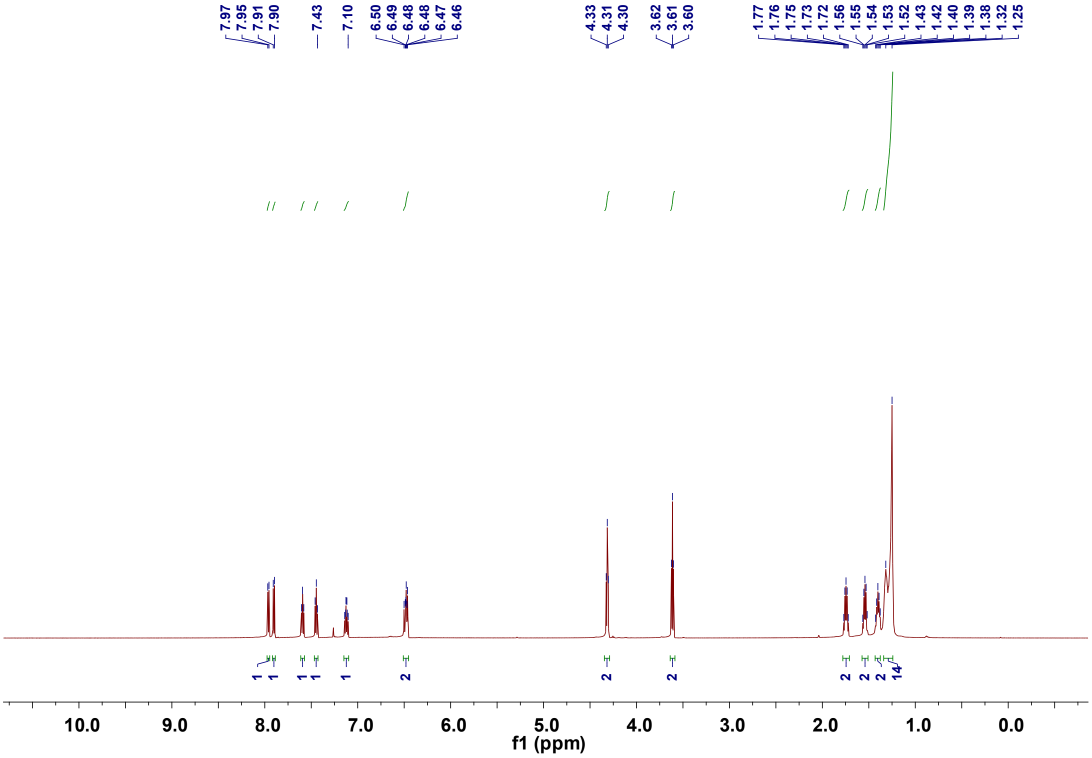


$^1$H NMR spectrum (600 MHz, $CDCl_3$, 298 K) of compound **10**.

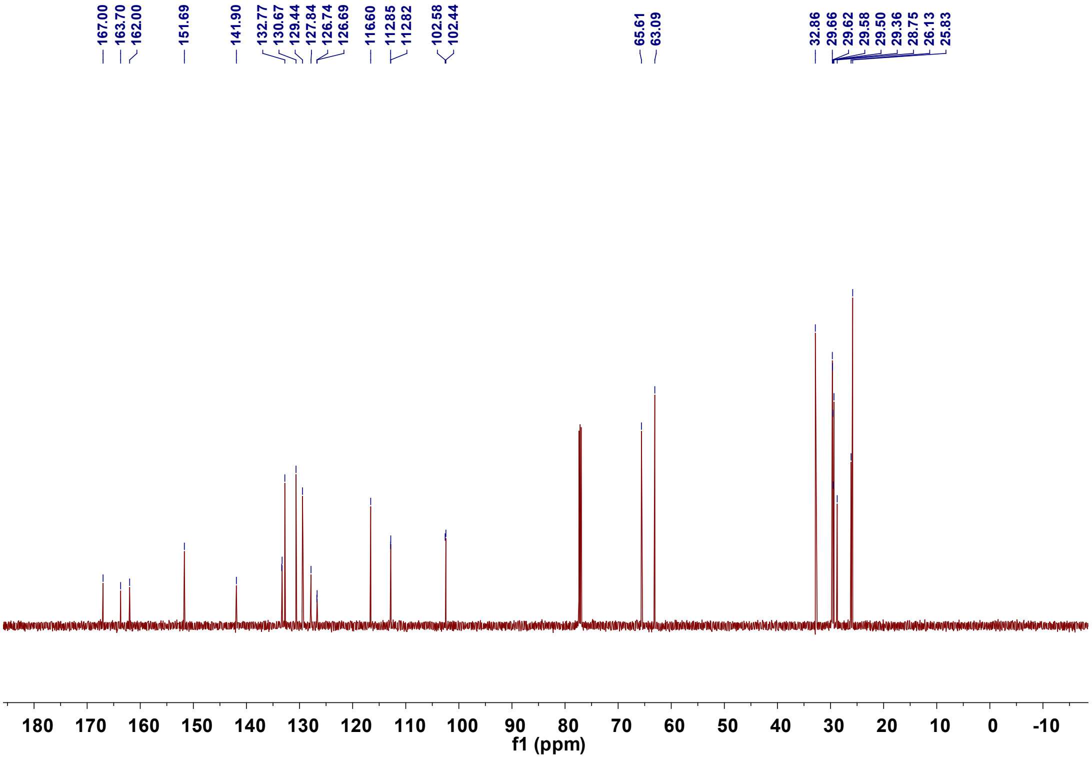


$^{13}$C NMR spectrum (150 MHz, $CDCl_3$, 298 K) of compound **10**.

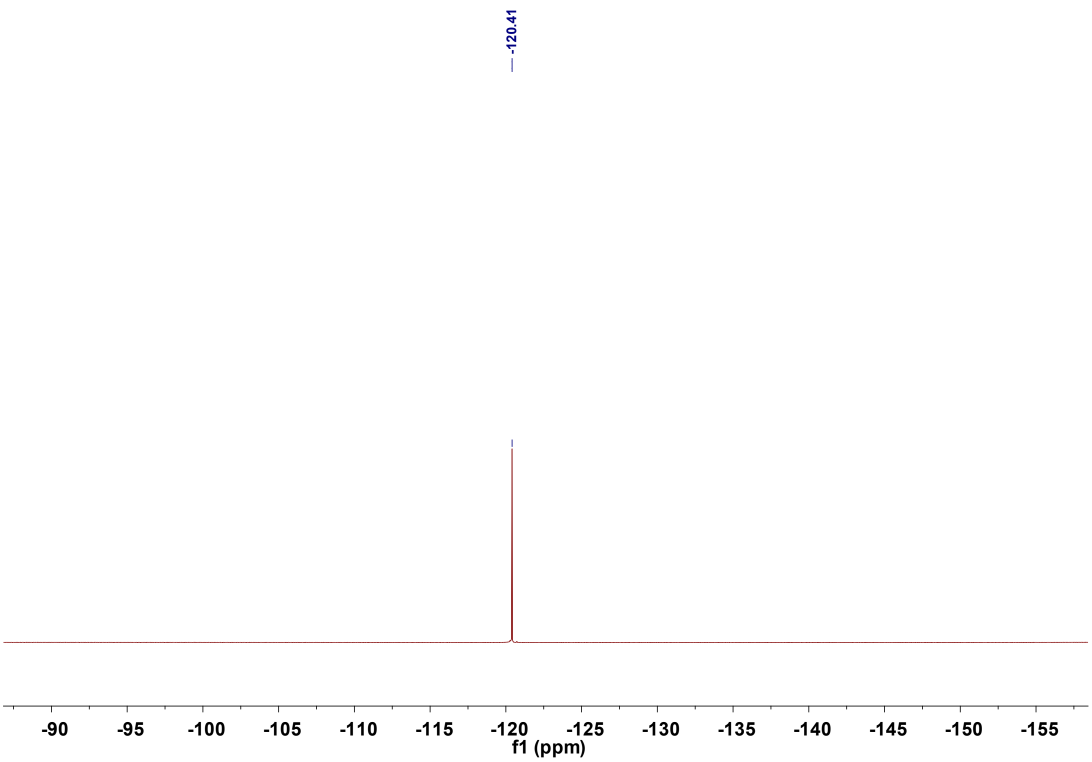


$^{19}$F NMR spectrum (564 MHz, $CDCl_3$, 298 K) of compound **10**.

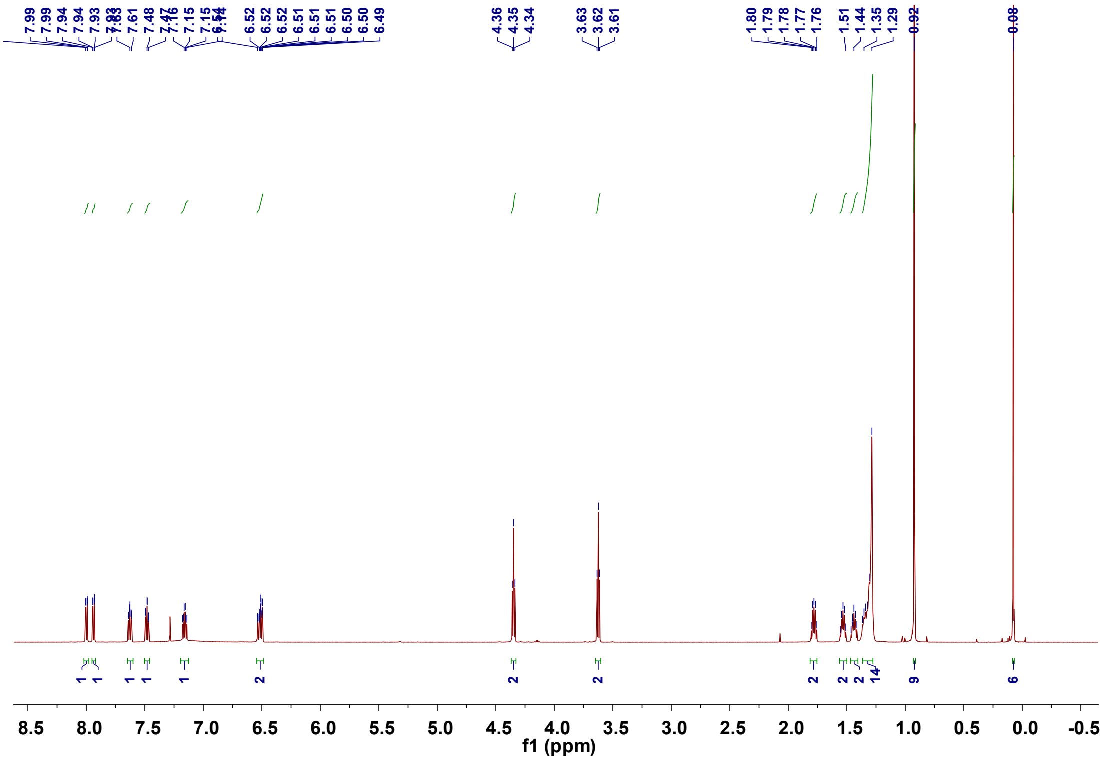


$^{1}$H NMR spectrum (600 MHz, $CDCl_3$, 298 K) of compound **11**.

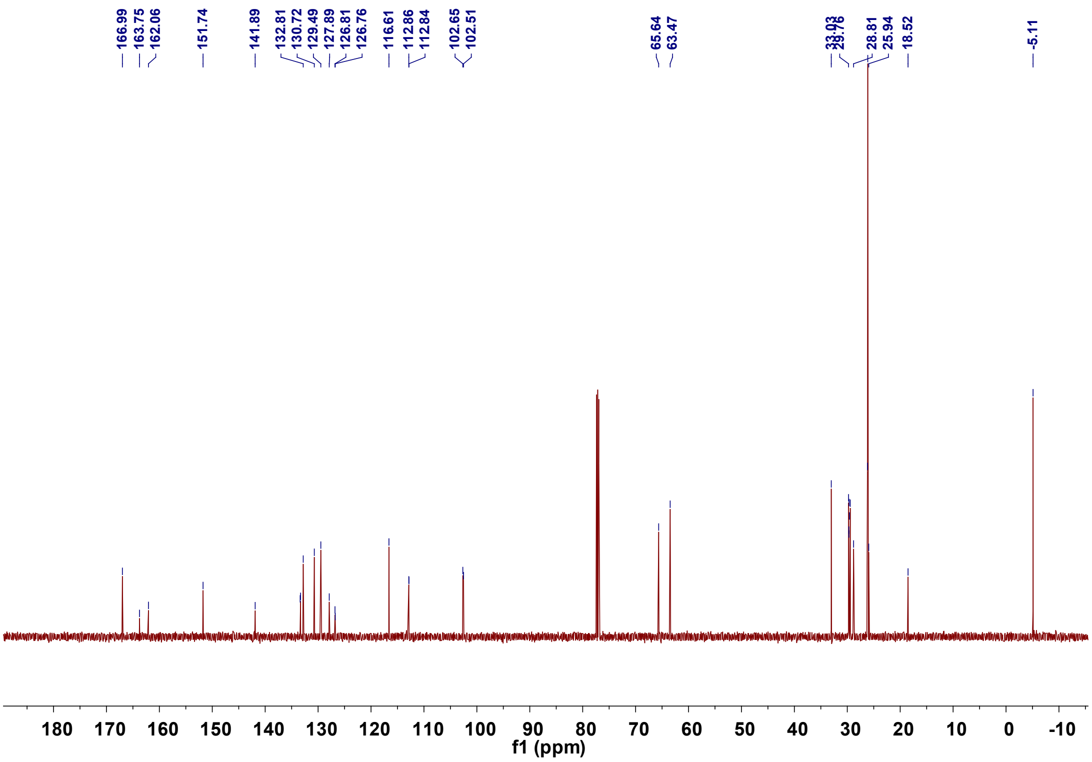


$^{13}C$ NMR spectrum (150 MHz, $CDCl_3$, 298 K) of compound **11**.

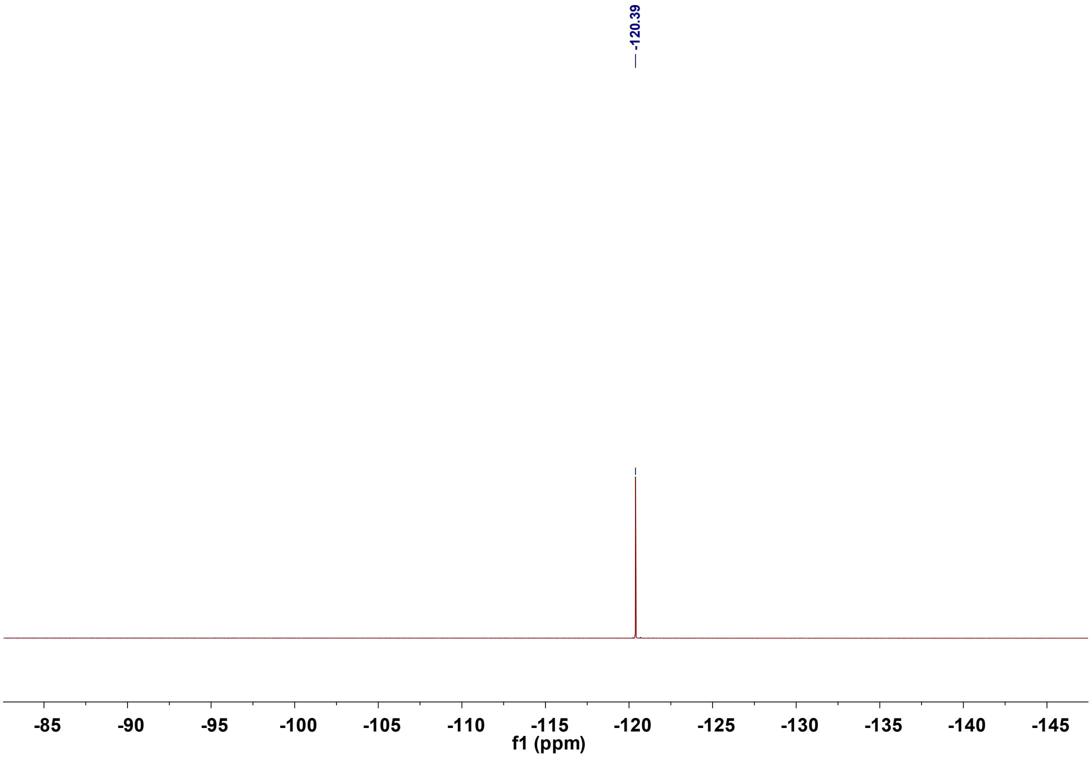


$^{19}F$ NMR spectrum (564 MHz, $CDCl_3$, 298 K) of compound **11**.

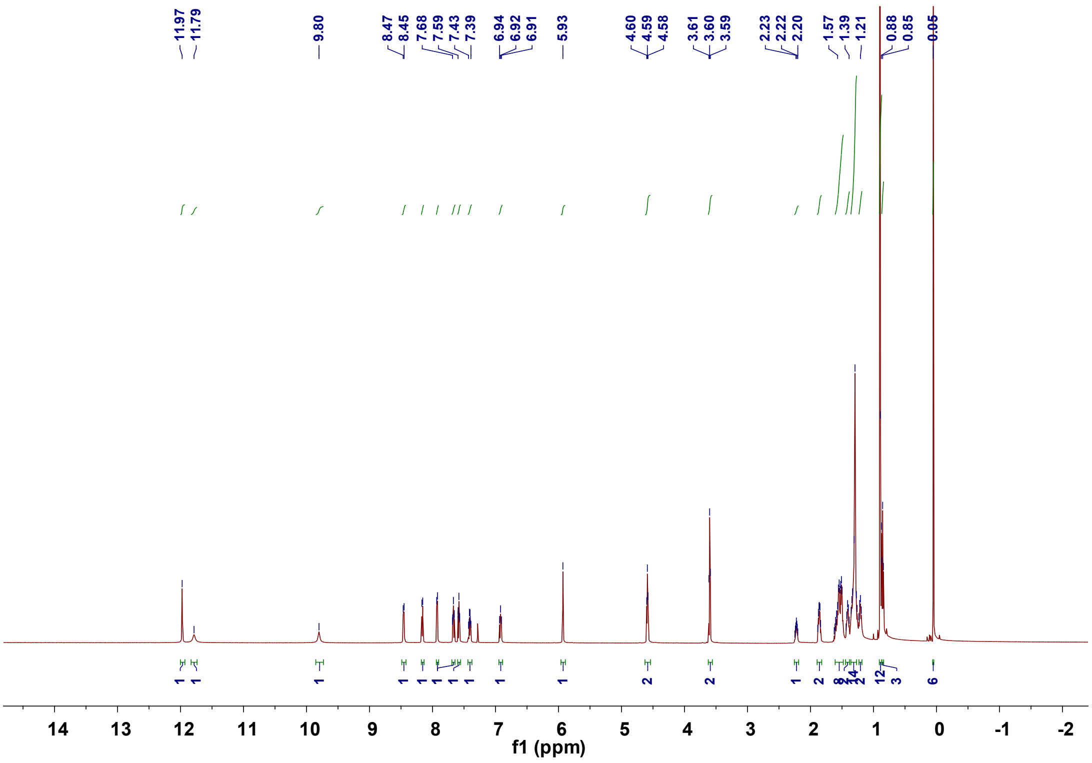

$^{1}$H NMR spectrum (600 MHz, $CDCl_3$, 298 K) of compound **12**.

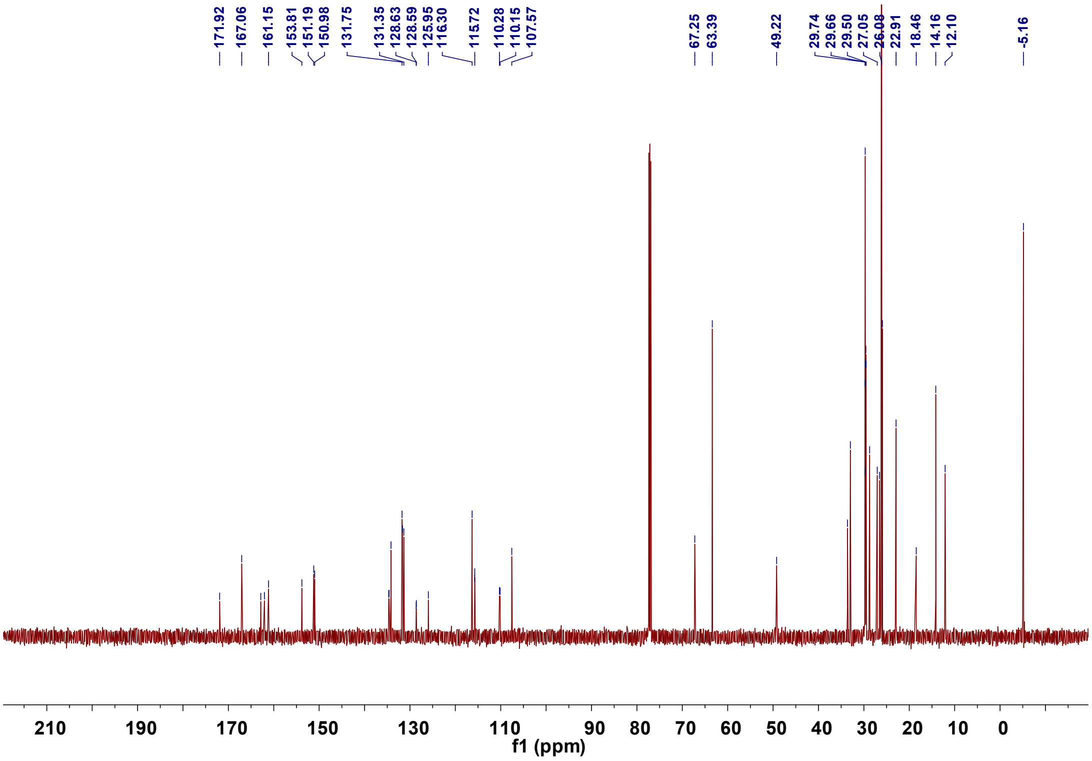

$^{13}$C NMR spectrum (150 MHz, $CDCl_3$, 298 K) of compound **12**.

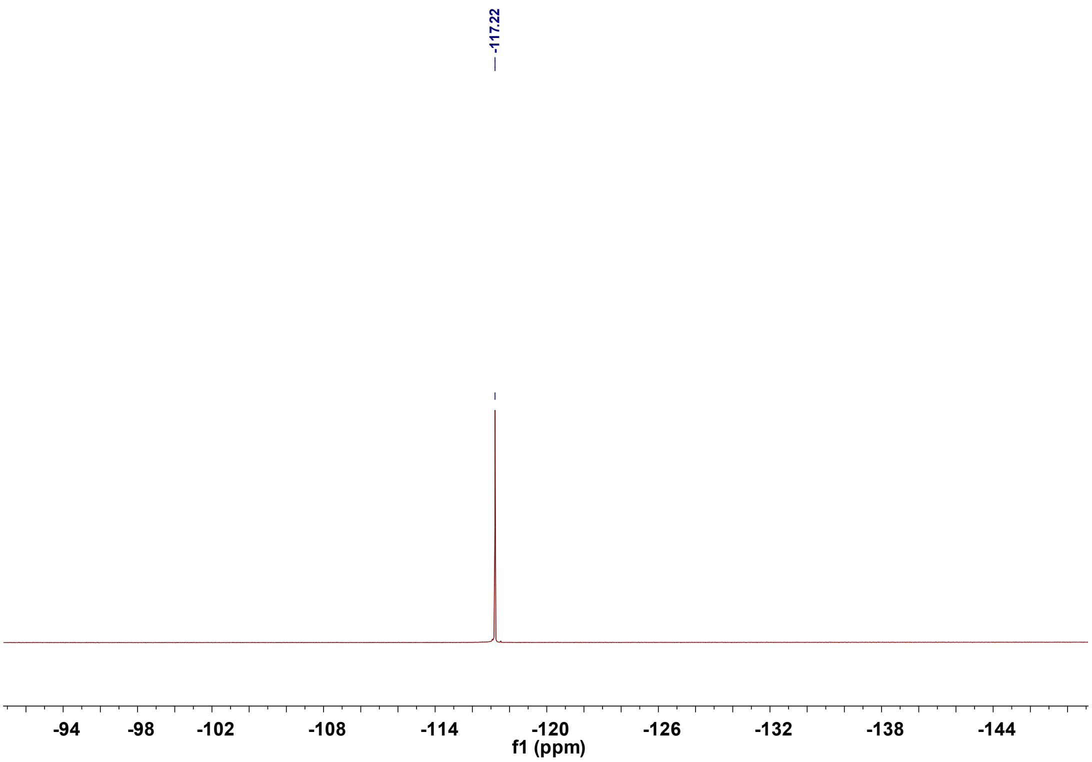


$^{19}F$ NMR spectrum (564 MHz, $CDCl_3$, 298 K) of compound **12**.

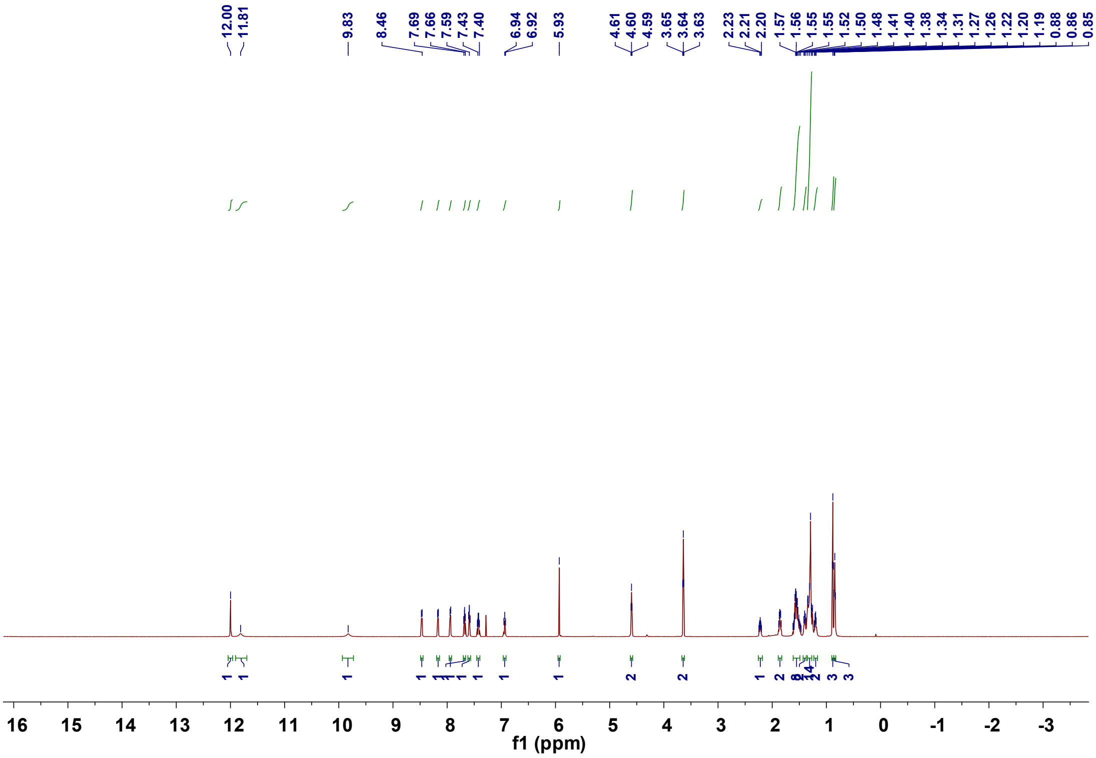


$^{1}H$ NMR spectrum (600 MHz, $CDCl_3$, 298 K) of compound **13**.

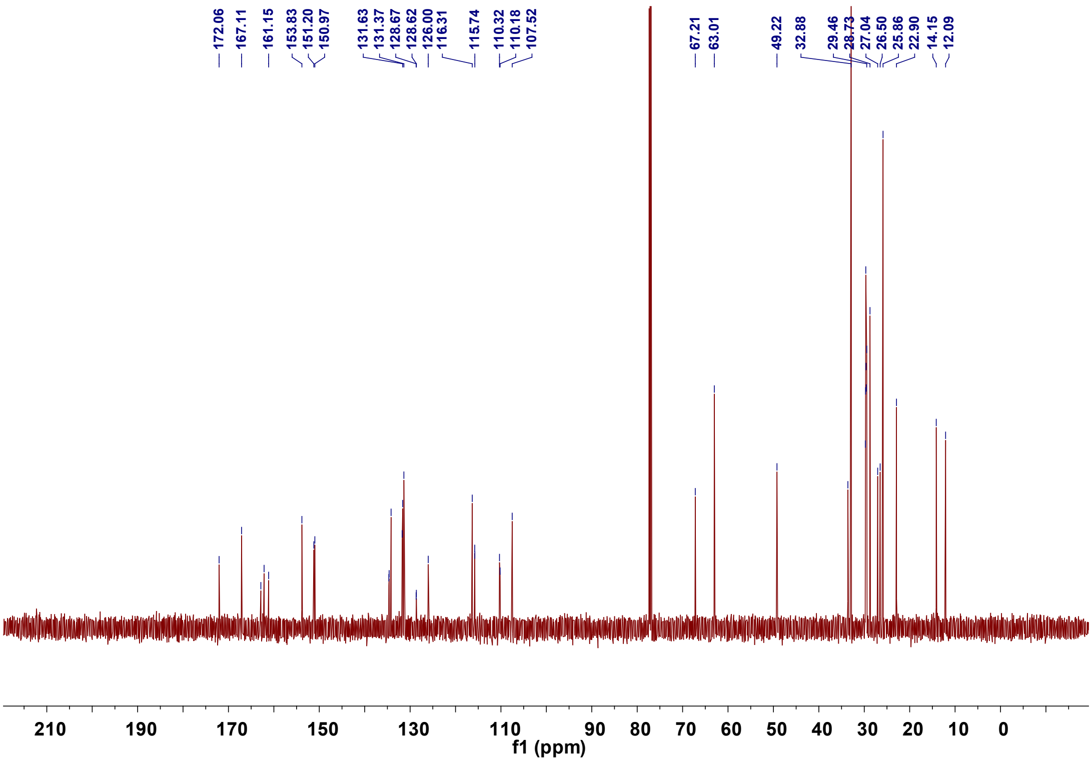


$^{13}$C NMR spectrum (150 MHz, $CDCl_3$, 298 K) of compound **13**.

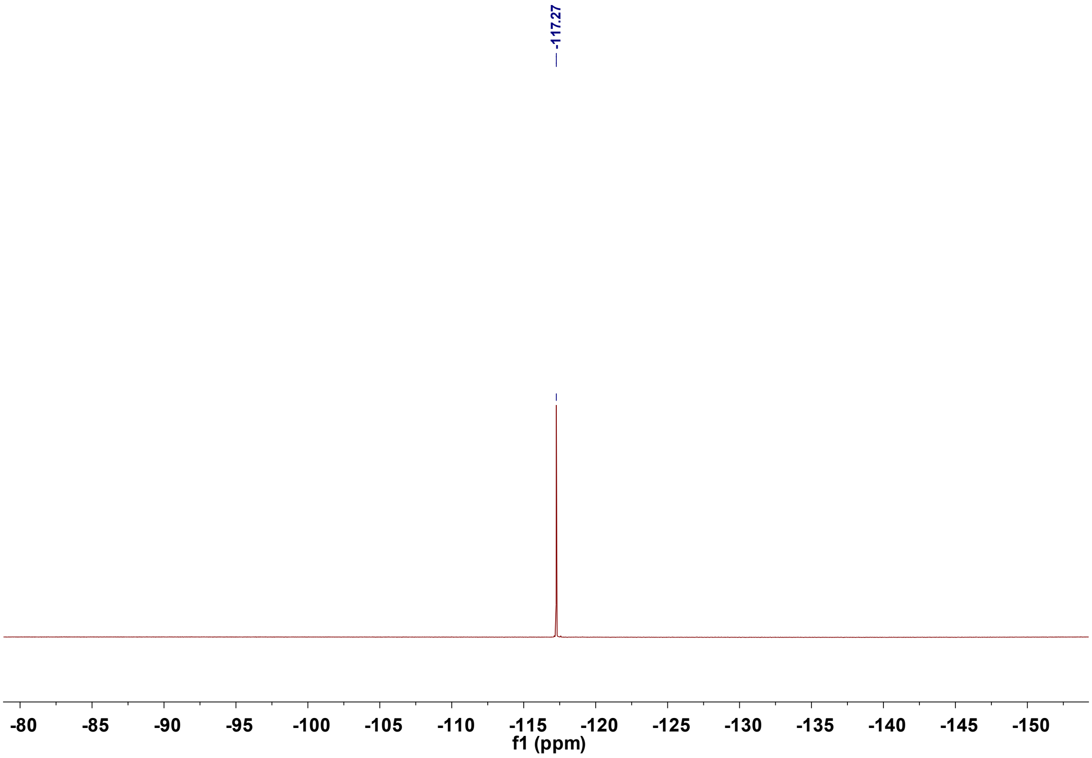


$^{19}$F NMR spectrum (564 MHz, $CDCl_3$, 298 K) of compound **13**.

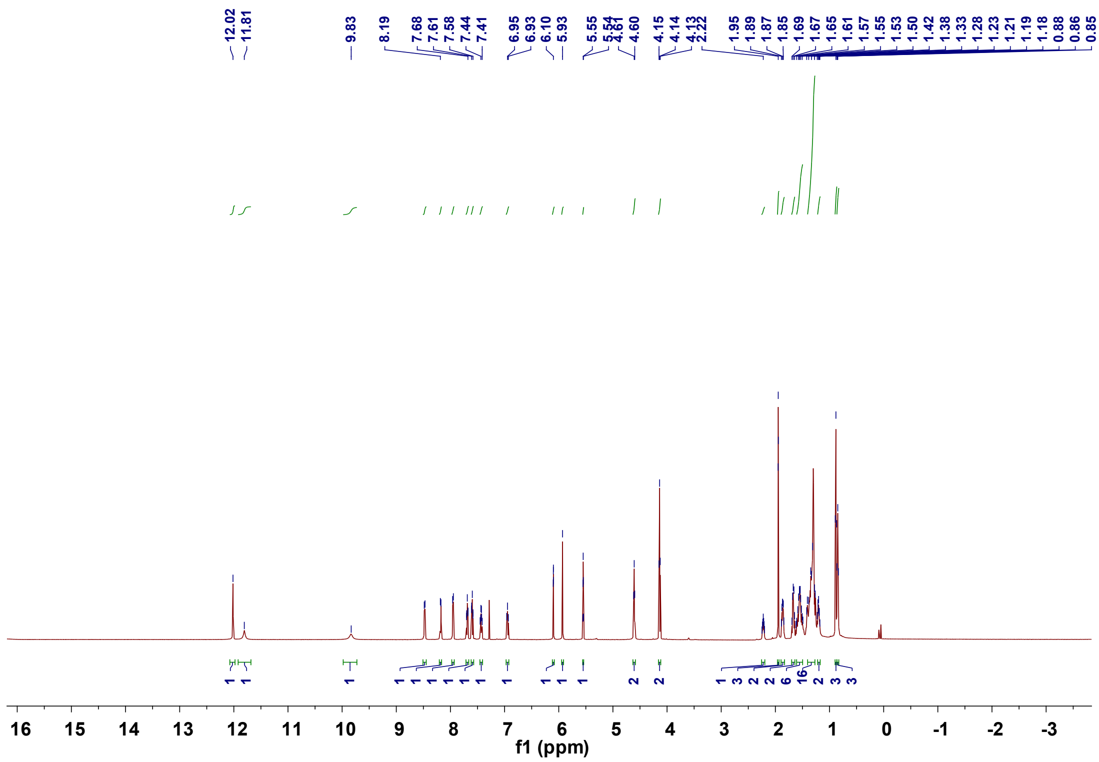

$^{1}$H NMR spectrum (600 MHz, $CDCl_3$, 298 K) of compound **15**.

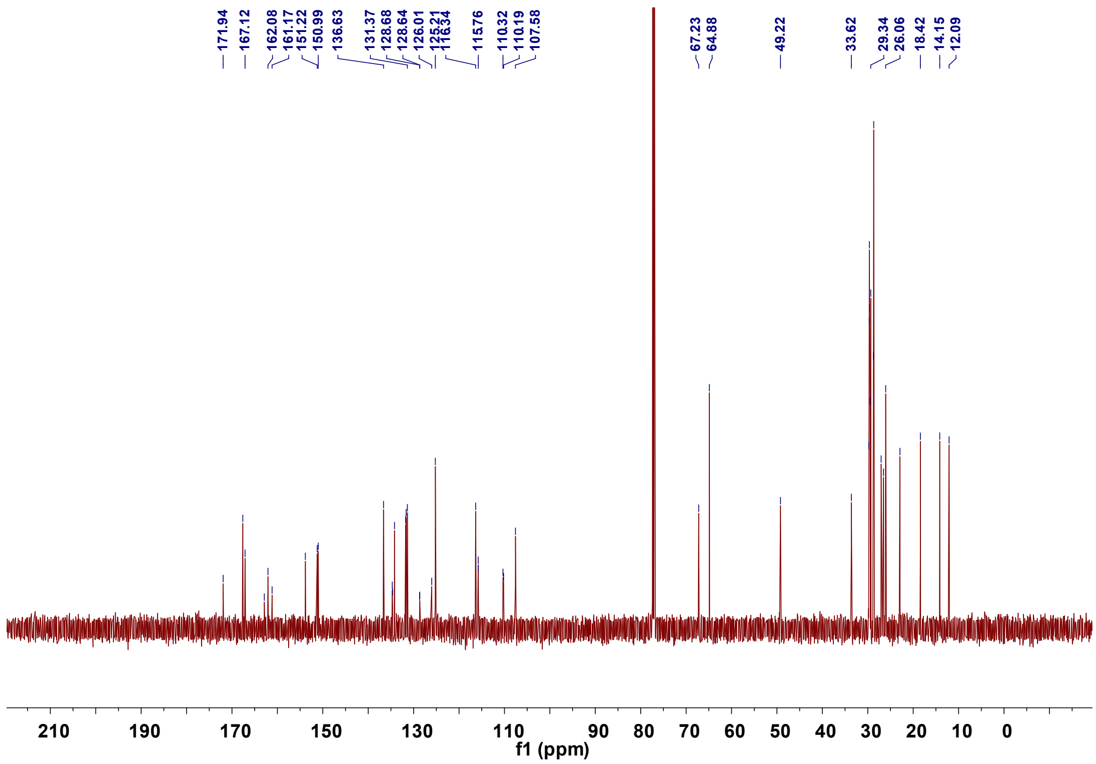

$^{13}$C NMR spectrum (150 MHz, $CDCl_3$, 298 K) of compound **15**.

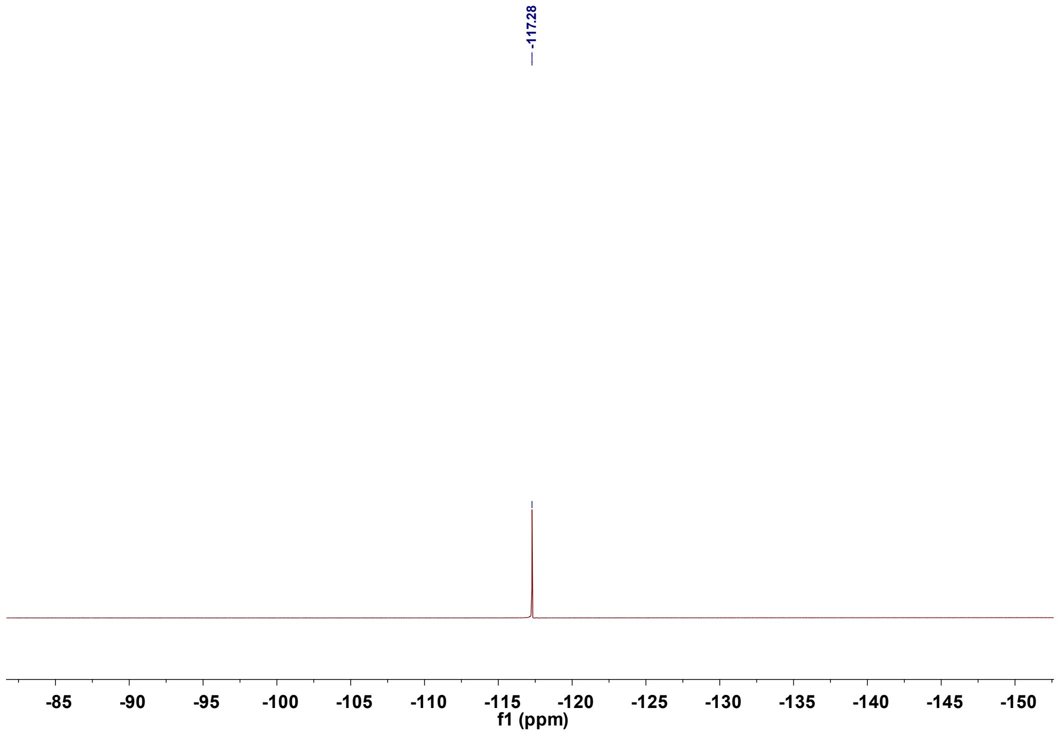


$^{19}F$ NMR spectrum (564 MHz, $CDCl_3$, 298 K) of compound **15**.

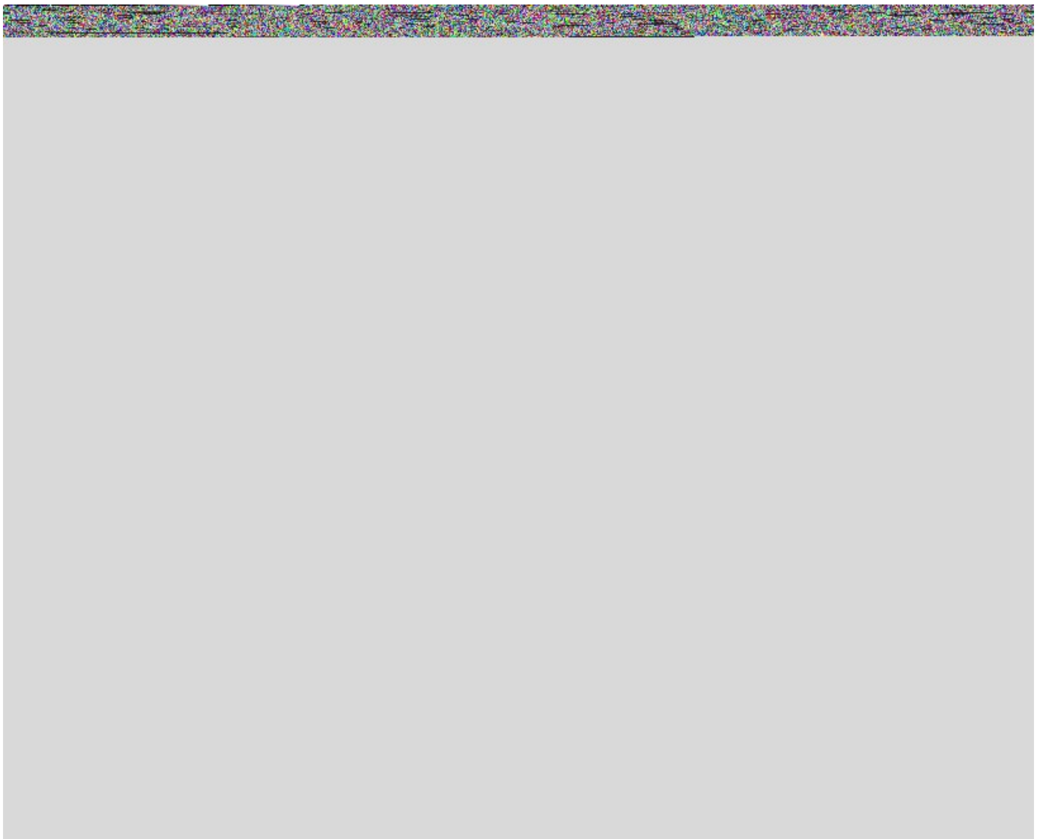

$^{1}H$ NMR spectrum (600 MHz, $CDCl_3$, 298 K) of **Azo-UPy-P**.

**Section 8: GPC trace of the polymer Azo-UPy-P**

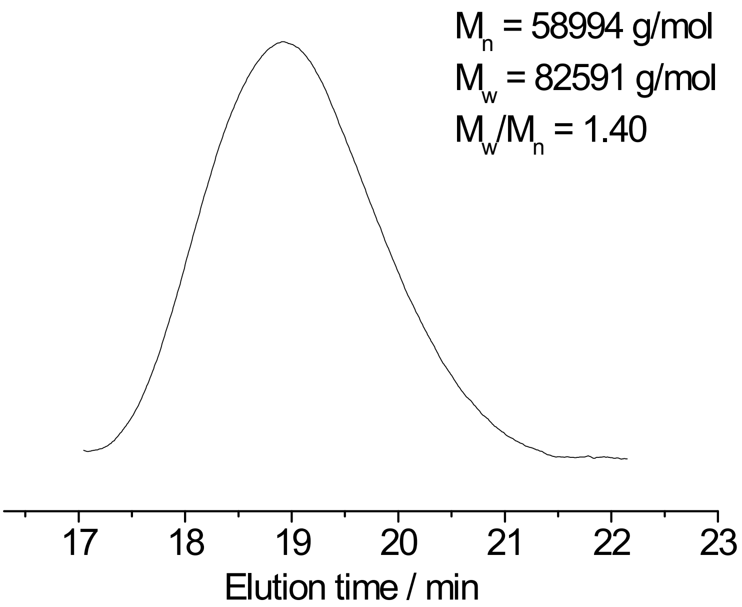


GPC trace of the polymer **Azo-UPy-P.**

## References


1. V. Chandrasekaran, E. Johannes, H. Kobarg, F. D. Sönnichsen and T. K. Lindhorst, *ChemistryOpen*, 2014, **3**, 99-108.

2. S. V. Shinde and P. Talukdar, *Chem. Commun.*, 2018, **54**, 10351-10354.

3. C. Mo, Z. Zhang, C. P. Guise, X. Li, J. Luo, Z. Tu, Y. Xu, A. V. Patterson, J. B. Smaill, X. Ren, X. Lu and K. Ding, *ACS Med. Chem. Lett.*, 2017, **8**, 543-548.

4. Z. Cai, S. Li, Y. Gao, L. Fu and G. Li, *Chem. Commun.*, 2018, **54**, 12766-12769.

5. H. M. Keizer, J. J. González, M. Segura, P. Prados, R. P. Sijbesma, E. W. Meijer and J. de Mendoza, *Chem.-Eur. J.*, 2005, **11**, 4602-4608.

6. Y.-F. Yin, M.-Y. Yun, L. Wu, H.-Y. Duan, X.-M. Jiang, T.-G. Zhan, J. Cui, L.-J. Liu and K.-D. Zhang, *Angew. Chem., Int. Ed.*, 2019, **58**, 12705-12710.
